# Deep phenotyping of a modified diabetic cardiomyopathy mouse model which reflects clinical disease progression

**DOI:** 10.1186/s13098-025-01913-3

**Published:** 2025-08-14

**Authors:** Narainrit Karuna, Lauren Kerrigan, Kevin Edgar, Oisin Cappa, David Simpson, Claire Tonry, David J. Grieve, Chris J. Watson

**Affiliations:** 1 Wellcome-Wolfson Institute for Experimental Medicine, Queen’s University Belfast, Belfast, UK; 2 Department of Pharmaceutical Care, Faculty of Pharmacy, Chiang Mai University, Chiang Mai, Thailand

**Keywords:** Diabetic cardiomyopathy, Metabolic syndrome, Diastolic dysfunction, Heart failure, Cardiac fibrosis

## Abstract

Diabetic cardiomyopathy (DbCM) is a progressive disease and common complication of metabolic diabetes. It is characterised by onset of cardiac structural and functional impairments and can lead to direct development of clinical heart failure (HF) or predispose to hypertensive/ischaemic stress. DbCM is a complex disease which involves several metabolic and pathogenic factors. We characterised an established high-fat diet/streptozotocin (HFD/STZ)-induced DbCM model incorporating typical features of human disease to determine its suitability for preclinical evaluation of novel therapeutics prior to advancement to human trials. Male C57BL/6J mice were randomised to HFD and single-dose STZ (100 mg/kg) or control diet (CD) and vehicle. HFD/STZ mice developed type 2 diabetes mellitus (T2DM), reflected by high fasting blood glucose and HbA1c levels, reduced β-cell function, and increased insulin resistance without systolic blood pressure alteration. Furthermore, HFD/STZ mice displayed progressive diastolic dysfunction, evidenced by decreased MV E/A ratio, together with elevated chronic left ventricular (LV) filling pressure parameters, measured by left atrial (LA) area and LA volume, compared to controls, in parallel with LV hypertrophy and fibrosis. Monocyte trafficking into diabetic hearts was identified by single-nuclei RNA sequencing analysis, which revealed an interferon-α response in DbCM mice, whilst plasma proteomics confirmed the involvement of inflammatory processes with elevated plasma C-reactive protein in DbCM progression. Taken together, our HFD/STZ-induced DbCM model exhibits a unique DbCM pre-clinical phenotype reflecting a "triple-hit" of human DbCM features comprising (1) T2DM with insulin resistance, (2) progressive diastolic dysfunction and LV remodelling, and (3) metabolic inflammation. This improved HFD/STZ-induced DbCM model supports clinically relevant research on DbCM progression from early stages to cardiac dysfunction and remodelling as the basis for translational investigation.

**Supplementary Information:**

The online version contains supplementary material available at 10.1186/s13098-025-01913-3.

## Introduction

Diabetic cardiomyopathy (DbCM) describes diabetes mellitus (DM)-associated changes in cardiac structure and function without other confounding cardiovascular factors, such as coronary artery disease (CAD) or hypertension [1]. Heart failure (HF) is classified into stages A to D, with DbCM categorised as asymptomatic stage B HF with a high prevalence of up to 67% in the community [2, 3]. Progression from stage B HF to symptomatic stage C HF is associated with a fivefold increase in mortality risk in both sexes [4]. Theoretically, early therapeutic intervention may attenuate or prevent the development of overt symptomatic HF in individuals with stage B HF. Many patients with DM show direct detrimental impacts on the myocardium, resulting in the progression of pathophysiologic processes, including left ventricular (LV) hypertrophy, diastolic and systolic dysfunction, and inflammation [5, 6]. Pre-emptive screening and identification of high-risk patients in the natural history of disease prior to the development of symptomatic HF would support paradigm shifts in clinical management. Such proactive approaches would represent practical and effective strategies to tackle the subclinical progression of DbCM prior to the clinical HF onset.

However, several questions about the uniqueness of DbCM and its transition from subclinical to symptomatic HF stages remain. Pre-clinical models support improved understanding of mechanistic aspects of diabetic complications, including DbCM; however, current pre-clinical models may not capture all human features of DbCM progression [7, 8]. Therefore, improved pre-clinical models that recapitulate early to late DbCM progression are needed to facilitate clinically relevant knowledge of disease mechanisms and enhanced therapeutic development. In support of this goal, we aimed to establish and characterise the high-fat diet/streptozotocin (HFD/STZ)-induced DbCM model through a combination of long-term HFD feeding and single-dose STZ (100 mg/kg i.p.) to promote progressive myocardial remodelling. Single-nuclei RNA sequencing of LV tissue and plasma proteomics analysis were performed to decipher the signature of DbCM progression in this model in relation to typical clinical presentation and assess its suitability to support impactful translational research.

## Materials and methods

### Animal study

Male C57BL/6J mice (Charles River UK) at eight weeks of age were fed with either HFD or control diet (CD) (n = 10 each) for two months prior to a single intraperitoneal injection of STZ (100 mg/kg) or sodium citrate vehicle and maintenance on HFD or CD for a further four months. Mice were housed in 12/12 h light/dark cycle with ad libitum access to food and water, with weekly weighing and regular welfare checks. Cardiac structure and function were assessed by monthly echocardiography, whilst systolic blood pressure was measured using tail-cuff plethysmography. After six months of study, mice were sacrificed by cervical dislocation. All study procedures complied with the United Kingdom Home Office Regulations and ASPA NI 1986 Regulations. This study was approved by the Animal Welfare and Ethical Review Body at Queen’s University Belfast.

### Echocardiography

Mice were induced with 5% isoflurane in O_2_, and anaesthesia maintained with 2% isoflurane throughout the procedure. All echocardiographic imaging was performed using a Vevo 3100 system (VisualSonics Inc., Canada) with a MX400 (20–46 MHz) linear array transducer. B-and M-mode images were acquired from the standard two-D parasternal long axis for measurement of superoinferior (SI) and anteroposterior (AP) dimensions. Mediolateral (ML) dimensions were measured from the parasternal short-axis view and peak mitral flow velocities in early diastole (E) and late diastole (A) by pulsed-wave Doppler in the apical four-chamber view. Left atria (LA) volume and area measurements were also performed, according to previous studies [9, 10]. LA volume was calculated by (4π × SI × AP × ML) / (3 × 2 × 2 × 2) and normalised by body surface area, whilst LA area was measured in the apical 4-chamber view by tracing the border of the LA.

### Diabetic parameters measurement

Blood was obtained from tail veins after fasting mice for 4 h, and glucose levels were measured by a glucometer (Glucomen® Areo, Menarini Diagnostics, UK). An A1CNow kit (BHR Pharmaceuticals Ltd, UK) was used to measure blood haemoglobin A1c (HbA1c). HOMA-β (homeostasis model assessment of β-cell function) was quantified using 20 × fasting insulin (µU/ml)/fasting glucose (mmol/L) − 3.5, and HOMA-IR (homeostasis model assessment of insulin resistance) quantified using fasting insulin (µU/ml) × fasting glucose (mmol/L)/22.5. Furthermore, 1/log fasting insulin (µU/ml) + log fasting glucose (mg/dL) was used to calculate QUICKI (quantitative insulin sensitivity check index) [11, 12].

### Plasma/tissue collection and histology studies

At 6 months of study, mice were sacrificed for collection of blood and heart tissue. Plasma was obtained following centrifugation of whole blood at 2000 RCF for 10 min, whilst hearts were dissected. Approximately half of the left ventricular tissue was placed in liquid nitrogen for protein and mRNA expression analyses, whilst the remainder was fixed in 10% neutral buffered formalin overnight and stored in phosphate-buffered saline and stored at 4 °C until tissue processing. Subsequently, heart tissue samples were embedded in paraffin and cut into 5 µm cross-sections. H&E and PicroSirius red staining were performed according to standard protocols. Cardiomyocyte cross-sectional area and collagen deposition were quantified by ImageJ (NIH, USA).

### Real-time qPCR assay

Total RNA was extracted from heart tissue using TRIzol™ reagent (15596026, Invitrogen™, UK). RNA samples were converted to cDNA (4387406; Thermo Fisher Scientific, US) according to the manufacturer’s instructions. Reverse transcription products were amplified on a LightCycler 480 instrument (Roche, Switzerland) using SYBR green master mix (Roche, Switzerland). All samples were measured in triplicate, and β-2-microglobulin (B2M) was used as a reference gene for calculation of relative gene expression by the 2^−ΔΔCT^ method. Primer sequences are listed in Supplement Table S1.

### Enzyme-linked immunosorbent assay (ELISAs)

ELISAs, including NT-proBNP (NBP2-76,775, Novus Biologicals) and insulin (90,080, Crystal Chem), were performed using plasma samples according to the manufacturer’s protocol.

### Single-nuclei RNA sequencing (snRNA-seq)

A detailed description of all experimental procedures and statistical tests can be found in the Supplementary material online. Heart ventricles of CD and HFD/STZ mice (n = 3 each) were used for single nuclei RNA sequencing (snRNA-seq). Data analysis of the snRNA data was carried out using Scanpy (version 1.9.6) [13], running on Python (version 3.11) or R (version 4.3.2) based on package compatibility. Pre-processing followed the standard procedures, including removing cells with < 200 expressed genes, eliminating genes expressed in < 3 cells, and discarding cells where the proportion of mitochondrial gene count exceeded 20%. Furthermore, doublet cells and ambient RNA were handled by Scrublet [14] and DecontX [15], respectively.

Expression data were then normalised, log transformed, and genes highly variable across cells were selected. Dimensionality reduction was performed, and graph clustering was generalised using the Leiden algorithm [16]. The marker genes for different cell populations were based on previously published studies [17–20] (Supplement Figure S1) and are listed in Supplement Table S2. Differential gene expression for markers amongst cell populations was performed by Wilcoxon rank-sum, whilst differentially expressed genes between groups (HFD/STZ vs control) in each cell population were identified using the Pseudobulk approach by edgeR [21]. The false discovery rate (FDR) < 0.05 and log twofold change > 1 were considered to indicate significant alterations in gene expression.

Composition of the cell populations was investigated using the speckle package [22]. The arcsin square root transformation was applied, and cell proportions were compared between conditions. To gain insight into biological functions, a multi-contrast gene set enrichment (Mitch) analysis was performed to identify key differentially regulated pathways [23]. The Mitch analysis uses a rank-MANOVA statistical approach to identify sets of genes that exhibit joint enrichment or divergent responses across multiple contrasts (cell populations). We implemented analysis against the Molecular Signatures Database (MSigDB) [24] using hallmark gene sets of *Mus musculus*.

### Proteomics-based mass spectrometry analysis

A detailed description of all experimental procedures and statistical tests can be found in the Supplementary material online. Proteomics analysis was applied to plasma samples (CD mice and HFD/STZ mice; n = 4 each) using the Evosep One LC system (EvoSep) coupled to a timsTOF Pro mass spectrometer (Bruker, Germany) with acquisition in either data-dependent acquisition (DDA) or data-independent acquisition-parallel accumulation-serial fragmentation (DIA-PASEF) mode. Raw mass spectrometry files (.d) were inputted for data processing, and spectral libraries were generated based on DDA files using the FragPipe computational platform (version 21.1) with MSFragger (version 4.0) [25, 26] with reference to protein sequence database of *Mus musculus* (UP000000589) from UniProt (downloaded on January 22, 2024). Protein inference was then performed using DDA-based spectral libraries in DIA-NN (version 1.81) [27], with the same settings as the previous DIA-PASEF workflow [28]; the software output was filtered at precursor FDR < 5%. Data processing was carried out, beginning with log 2 transformation of the data, with filtering of any proteins observed in < 50% of samples, resulting in 201 unique proteins. The dataset was normalised by Cyclic Loess from the limma package [29], and missing values were imputed using random draws from a manually defined left-shifted Gaussian distribution (shift = 1.8, scale = 0.3). Differentially expressed proteins were identified by application of a t-test, and the Benjamini and Hochberg (BH) method was used for adjusted P.

### Statistical analysis

All data are presented as mean ± SD unless otherwise stated. Normality assumptions of the data distribution were tested using the Shapiro–Wilk test. Analyses were performed with GraphPad Prism 10 software (GraphPad, USA). Unpaired two-tailed Student’s t-tests or Mann–Whitney U test was used to determine the statistical difference between the two groups as appropriate. Moreover, R programme (version 4.3.2) and Python (version 3.11) with relevant packages were used for snRNA-seq and proteomics data analysis. For all statistical comparisons, P-value < 0.05 was considered as statistically significant. We performed a power analysis for snRNA-seq experiment using the scPower website (https://scpower.helmholtz-muenchen.de) [30], setting the number of samples to 3, the cell type frequency to 0.25, the detection power at least 0.80, resulted in a requirement of at least 5500 cells/nuclei per sample to have sufficient power for differential expression analysis (Supplement Figure S2A). An a priori power analysis was performed using the pwr package (https://github.com/heliosdrm/pwr) in R to estimate the sample size required to detect differential protein expression. We assumed a log₂ fold change of 1 and SD of 0.4 (Cohen’s d = 2.5), with a two-sided significance level of 0.05. Power estimates across sample sizes from 2 to 6 per group (Supplementary Figure S2B) indicated that n = 4 per group yields > 80% power, supporting the chosen design for detecting significant difference.

## Results

### HFD/STZ-induced DbCM model develops weight gain, diabetes, and insulin resistance.

**Fig. 1:**
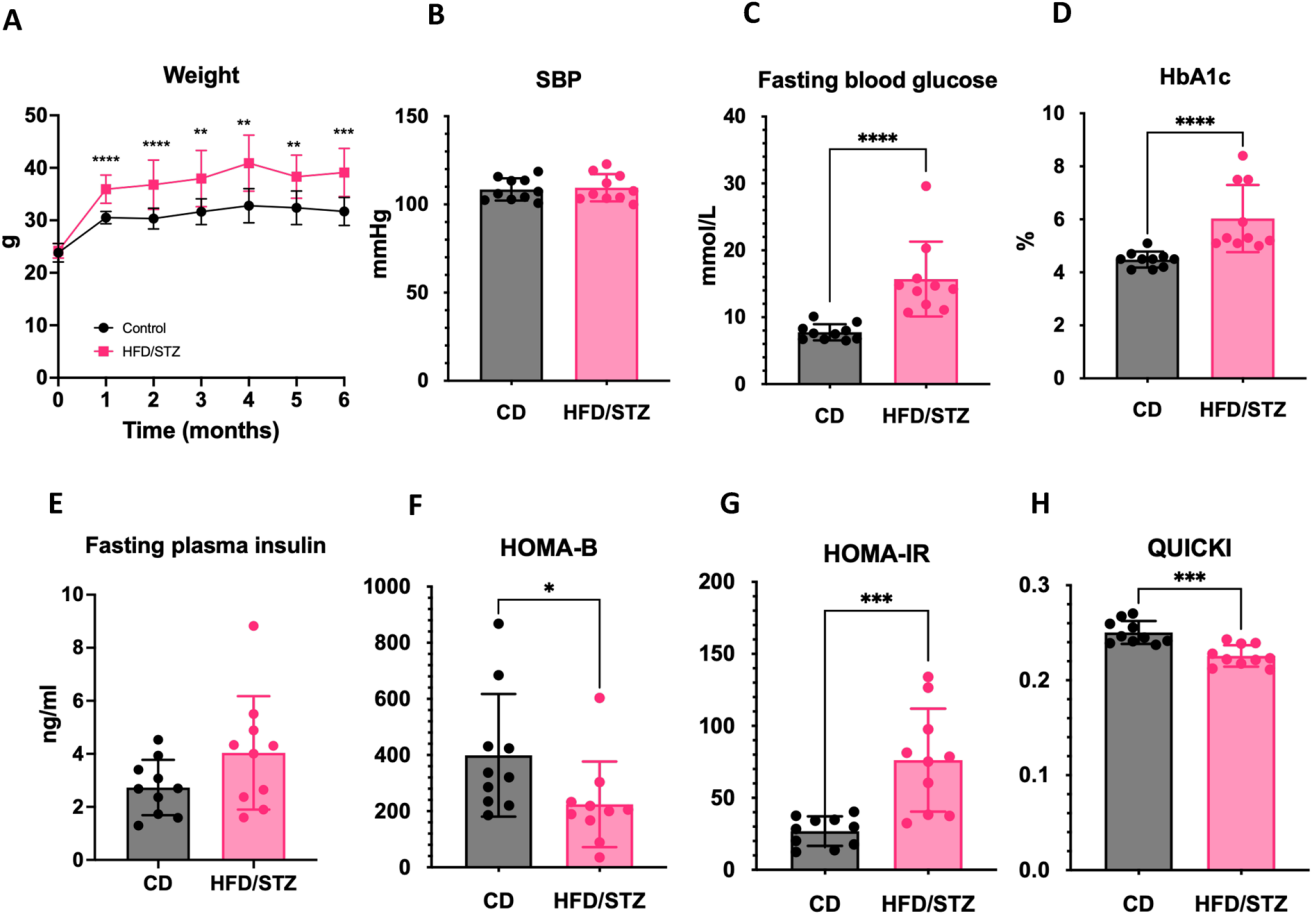
Characterisation of diabetic cardiomyopathy (DbCM) mouse model induced through combination of long-term high fat diet and single dose streptozotocin. **A** Time course of absolute body weight (in grams) of CD mice and HFD/STZ mice. **B** Systolic blood pressure, **C**, **D** Fasting blood glucose and HbA1c levels, **E** Fasting plasma insulin, and **F**–**H** HOMA-beta, HOMA-IR, and QUICKI at 6 months of study. *CD* control diet, *HFD/STZ* high fat diet/streptozotocin, *HOMA-beta* homeostatic model assessment of beta cells, *HOMA-IR* homeostatic model assessment of insulin resistance, *QUICKI* quantitative insulin sensitivity check index. Data presented as mean ± SD. * P-value < 0.05, **P-value < 0.01, ***P-value < 0.001, ****P-value < 0.0001

The HFD/STZ-induced DbCM model was induced by HFD for six months with single STZ injection (100 mg/kg) at two months of study. HFD/STZ mice significantly increased body weight after one month of diet compared to controls (CD mice), which was maintained until the end of the study (all P-values < 0.05; Fig. 1A). There was no difference in systolic blood pressure between groups at 6 months (Fig. 1B). Hyperglycaemic state was evident in HFD/STZ mice at the end of the study, indicated by increased fasting blood glucose (15.7 ± 5.6 vs 7.8 ± 1.2 mmol/L) and HbA1c levels (6.0 ± 1.3 vs 4.5 ± 0.3%) compared to control mice (all P-value < 0.0001; Fig. 1C, D). Moreover, our HFD/STZ-induced DbCM model recapitulated the type 2 DM (T2DM) model with a higher level of fasting plasma insulin, suggesting hyperinsulinemic state (Fig. 1E). β-cell function (HOMA-B) was significantly decreased (P-value = 0.0147), along with increased insulin resistance (HOMA-IR) (P-value = 0.0005) and reduced insulin sensitivity (QUICKI) (P-value = 0.0002) in HDF/STZ mice (Fig. 1F–H). Taken together, our HFD/STZ-induced DbCM model clearly recapitulates clinical T2DM, reflected by high blood glucose, weight gain, and insulin dysregulation.

### Longitudinal analysis of cardiac function and structure indicates progressive development of DbCM

**Fig. 2:**
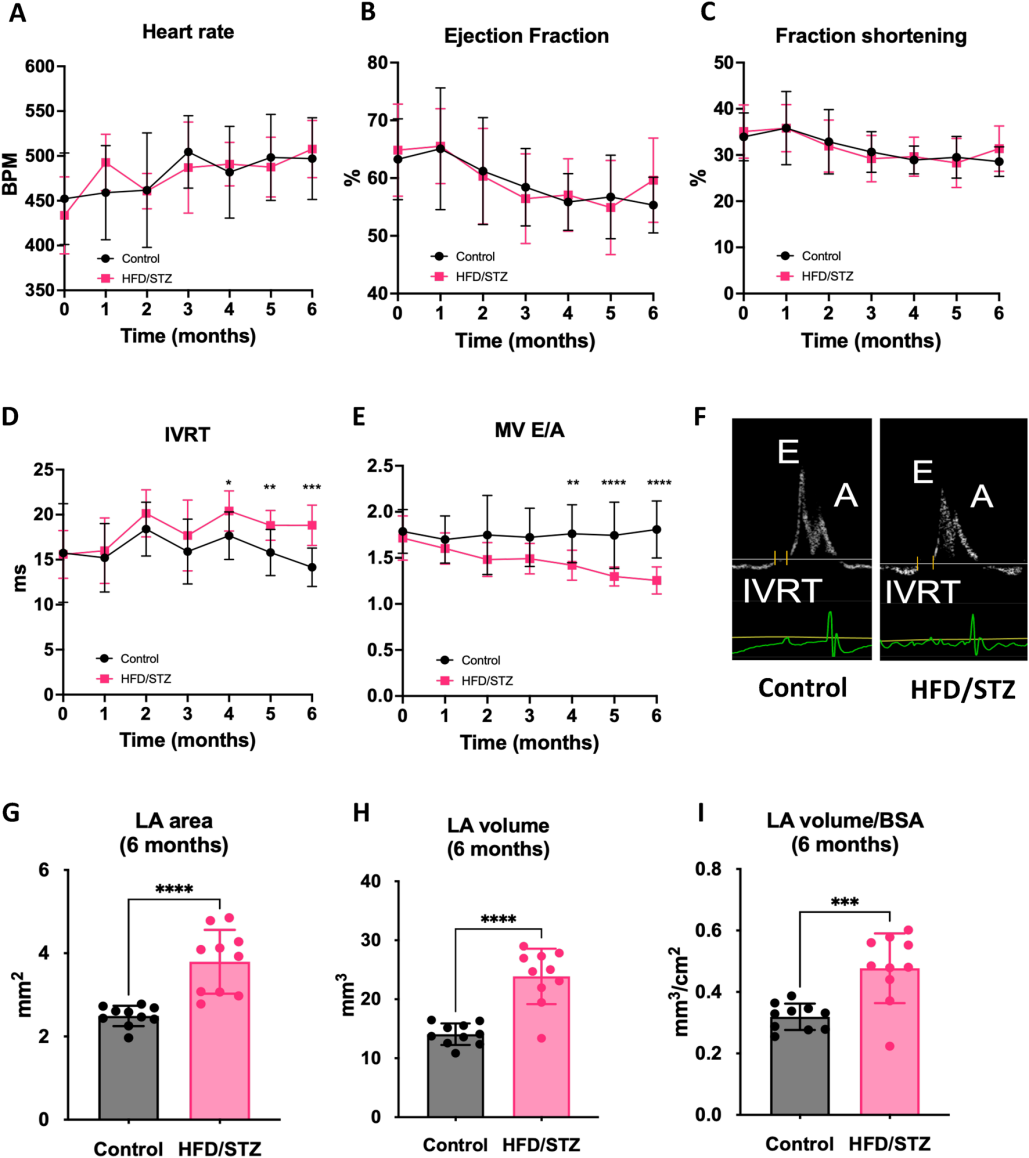
Longitudinal measurement of left ventricular function using echocardiography. Time course of **A** Heart rate (beats per minute), **B**, **C** Ejection fraction and fractional shortening, **D** IVRT, **E** Ratio of pulse wave Doppler E wave to A wave amplitude, and **F** Representative images of mitral valve flow by Pulse Wave Doppler. **G** Left atrial area, and **H**, **I** Left atrial volume at 6 months of study. *CD* control diet, *HFD/STZ* high fat diet/streptozotocin, *BPM* beats per minute, *IVRT* isovolumic relaxation time, *MV E/A ratio* early to late diastolic mitral flow velocity, *LA* left atrial, *BSA* body surface area. Data presented as mean ± SD. *P-value < 0.05, **P-value < 0.01, ***P-value < 0.001, ****P-value < 0.0001

**Fig. 3:**
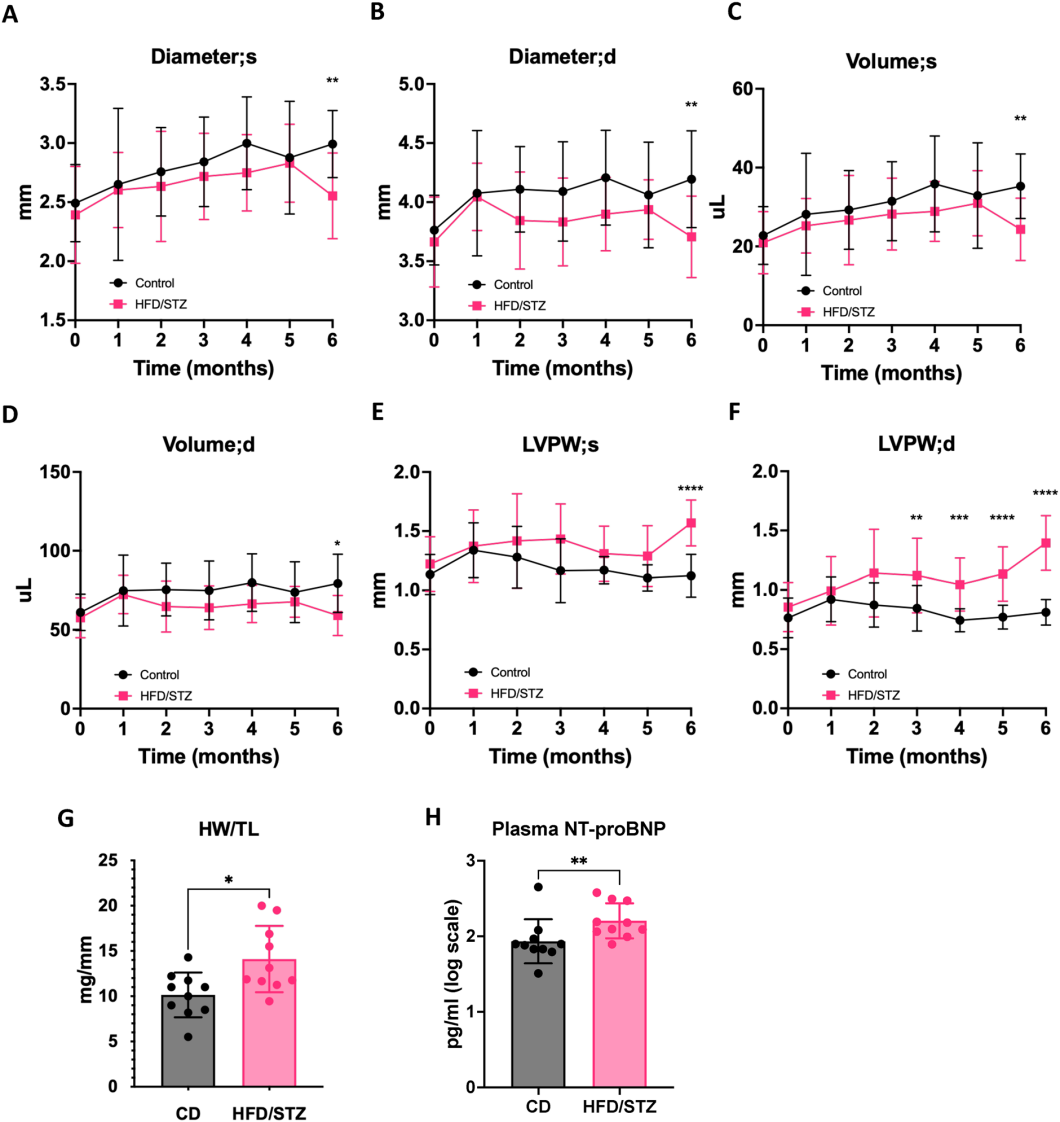
Longitudinal measurement of left ventricular structure using echocardiography. Time course of **A**, **B** Left ventricular diameter during systole and diastole, **C**, **D** Left ventricular volume during systole and diastole, and **E**, **F** Left ventricular posterior wall thickness during systole and diastole. **G** Heart weight/Tibia length, and **H** Plasma NT-proBNP level at 6 months of study. *CD* control diet, *HFD/STZ* high fat diet/streptozotocin, *LVPW* left ventricular posterior wall, *HW* heart weight, *TL* tibia length, *NT-proBNP* N-terminal pro B-type natriuretic peptide. Data presented as mean ± SD. *P-value < 0.05, **P-value < 0.01, ***P-value < 0.001, ****P-value < 0.0001

Serial echocardiography was performed at monthly intervals to study the progression of cardiac function and structure in HFD/STZ mice relative to controls. HFD/STZ mice developed diastolic dysfunction (Fig. 2D, E), which was evident at 4 months and maintained for the duration of the study, without changes in ejection fraction, fractional shortening, or heart rate (Fig. 2A-C). Specifically, IVRT was prolonged in HFD/STZ mice at 4, 5, and 6 months (all P-value < 0.05; Fig. 2D), whilst MV E/A ratio progressively decreased over the same timeframe in HFD/STZ mice compared with control mice (1.4 ± 0.2 vs 1.8 ± 0.3, P-value = 0.0071 at 4 months; 1.3 ± 0.1 vs 1.8 ± 0.4, P-value < 0.0001 at 5 months; 1.3 ± 0.2 vs 1.8 ± 0.3, P-value < 0.0001 at 6 months) (Fig. 2E, F). Chronic LV filling pressure effects were also evident in HFD/STZ mice as enlarged LA area and LA volume at 6 months of study (Fig. 2G–I) (all P-value < 0.05). In addition to observed functional alterations, HFD/STZ mice showed reduced LV diameter and LV volume in both systole and diastole compared to control mice at 6 months of study (Fig. 3A–D; all P-values < 0.05), suggesting cardiac chamber alteration.

**Fig. 4:**
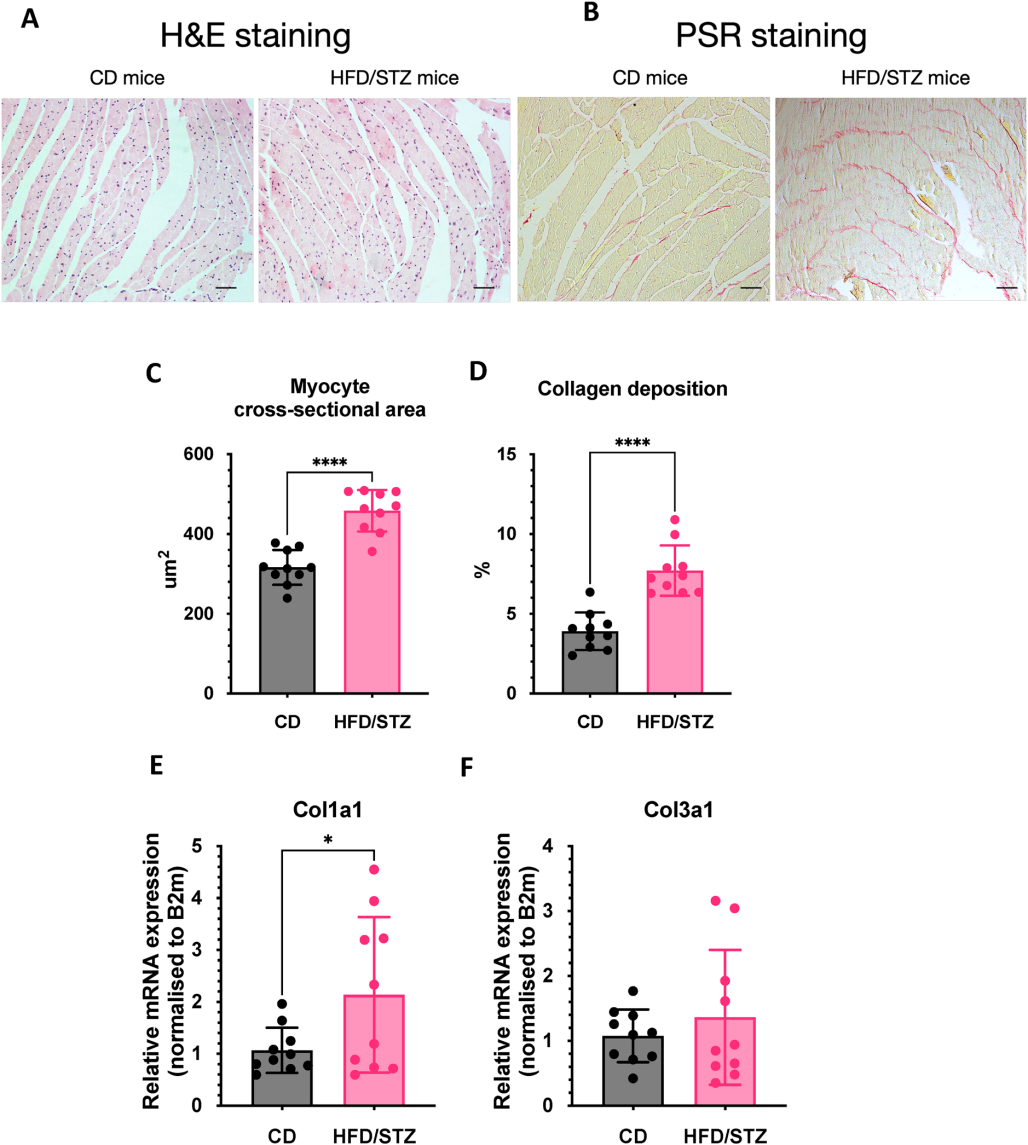
Cardiac remodelling analysis. **A**, **B** H&E staining and PicroSirius Red staining, **C**, **D** Quantification of cardiomyocyte cross-sectional area and collagen deposition, and **E**, **F** Col1a1 and Col3a1 expression by RT-qPCR in LV tissue from DbCM and CD mice at 6 months. *CD* control diet, *HFD/STZ* high fat diet/streptozotocin, *H&E* hematoxylin and eosin, *PSR* picrosirius red. Data presented as mean ± SD. *P-value < 0.05, ****P-value < 0.0001

Additionally, LV posterior wall during systolic phase showed an increase at 6 months of study for HFD/STZ mice vs control mice (1.6 ± 0.2 vs 1.1 ± 0.2, P-value < 0.0001) (Fig. 3E). It was noted that the LV posterior wall during the diastolic phase was enlarged in HFD/STZ mice, compared to control mice at 3 months (1.1 ± 0.3 vs 0.9 ± 0.2 mm, P-value = 0.0089), 4 months (1.1 ± 0.2 vs 0.7 ± 0.1 mm, P-value = 0.0003), 5 months (1.1 ± 0.2 vs 0.8 ± 0.1, P-value < 0.0001), and 6 months (1.4 ± 0.2 vs 0.8 ± 0.1, P-value < 0.0001) (Fig. 3F). Moreover, HW/TL was significantly higher in HFD/STZ mice (14.1 ± 3.7 mg/mm) in comparison to controls (10.1 ± 2.5, P-value = 0.0110) (Fig. 3G). Plasma NT-proBNP level was measured to reveal cardiac stress condition, and this was significantly increased in HFD/STZ mice (Fig. 3H). To assess tissue-level changes, histological analysis of LV sections was performed, which revealed increased cardiomyocyte cross-sectional area and collagen deposition (all P-value < 0.0001) in HFD/STZ compared to CD mice (Fig. 4A–D). Cardiac tissue RT-qPCR for pro-fibrotic genes confirmed that *Col1a1* expression was induced in the diabetic heart, compared to the control group (P-value = 0.0440), while *Col3a1* expression was higher in the diabetic heart but did not reach a significant level (Fig. 4E, F). Collectively, these data characterise our HFD/STZ induction protocol as a clinically relevant model of DbCM, which shows progressive diastolic dysfunction with preserved systolic function and blood pressure, in parallel with typical metabolic and LV remodelling alterations, thereby highlighting its suitability for translational research.

### Single-nuclei RNA sequencing deciphers key inflammatory drivers of DbCM progression

**Fig. 5:**
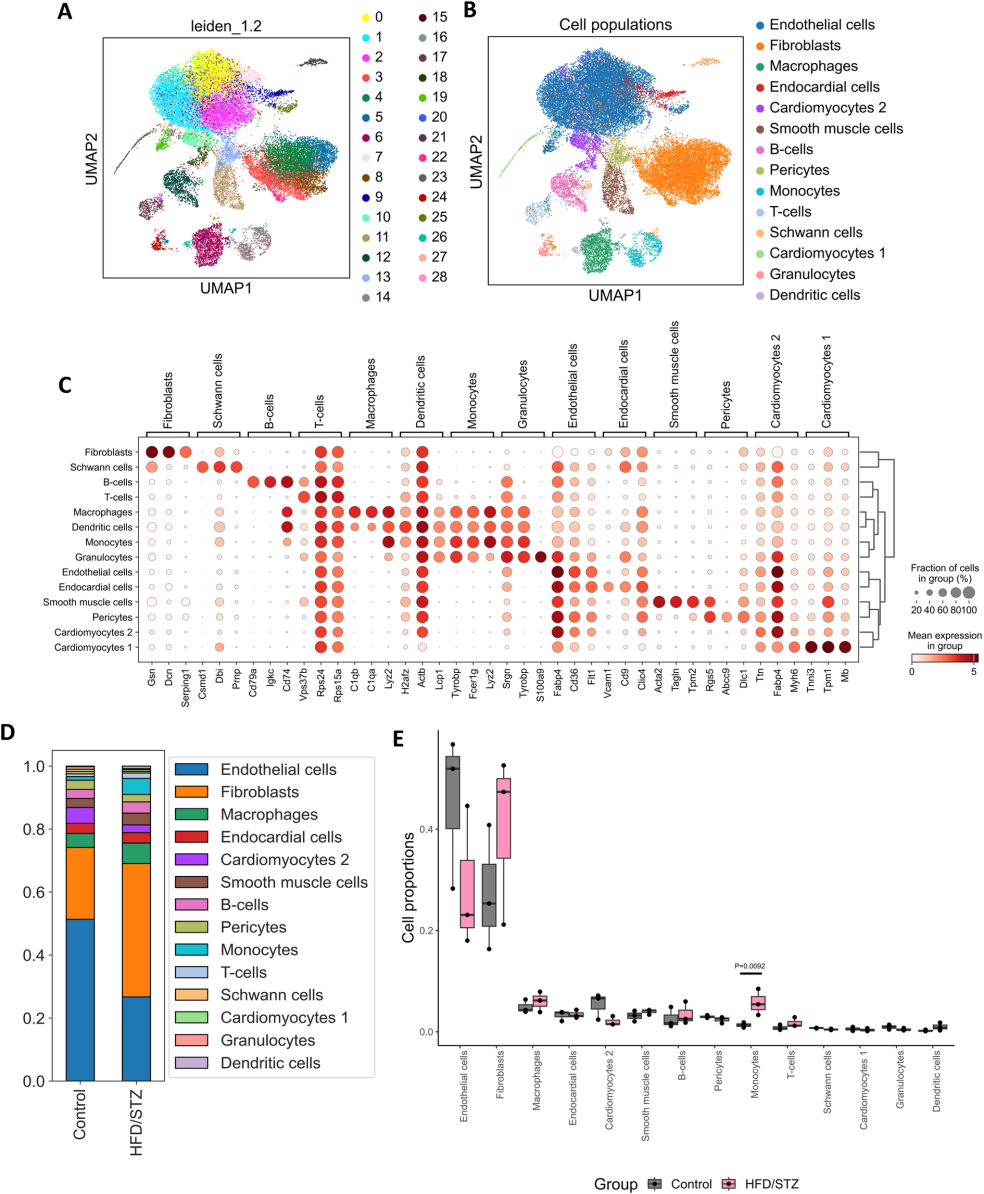
Single-nuclei RNA sequencing analysis. **A**, **B** Clustering and major cell populations using Leiden algorithm and established cell markers, **C** Top 5 signature gene expressions per cell population, **D** Cell proportions per sample group, and **E** Composition analysis across sample groups, using LV tissue from DbCM and CD mice at 6 months. *CD* control diet, *HFD/STZ* high fat diet/streptozotocin

**Fig. 6:**
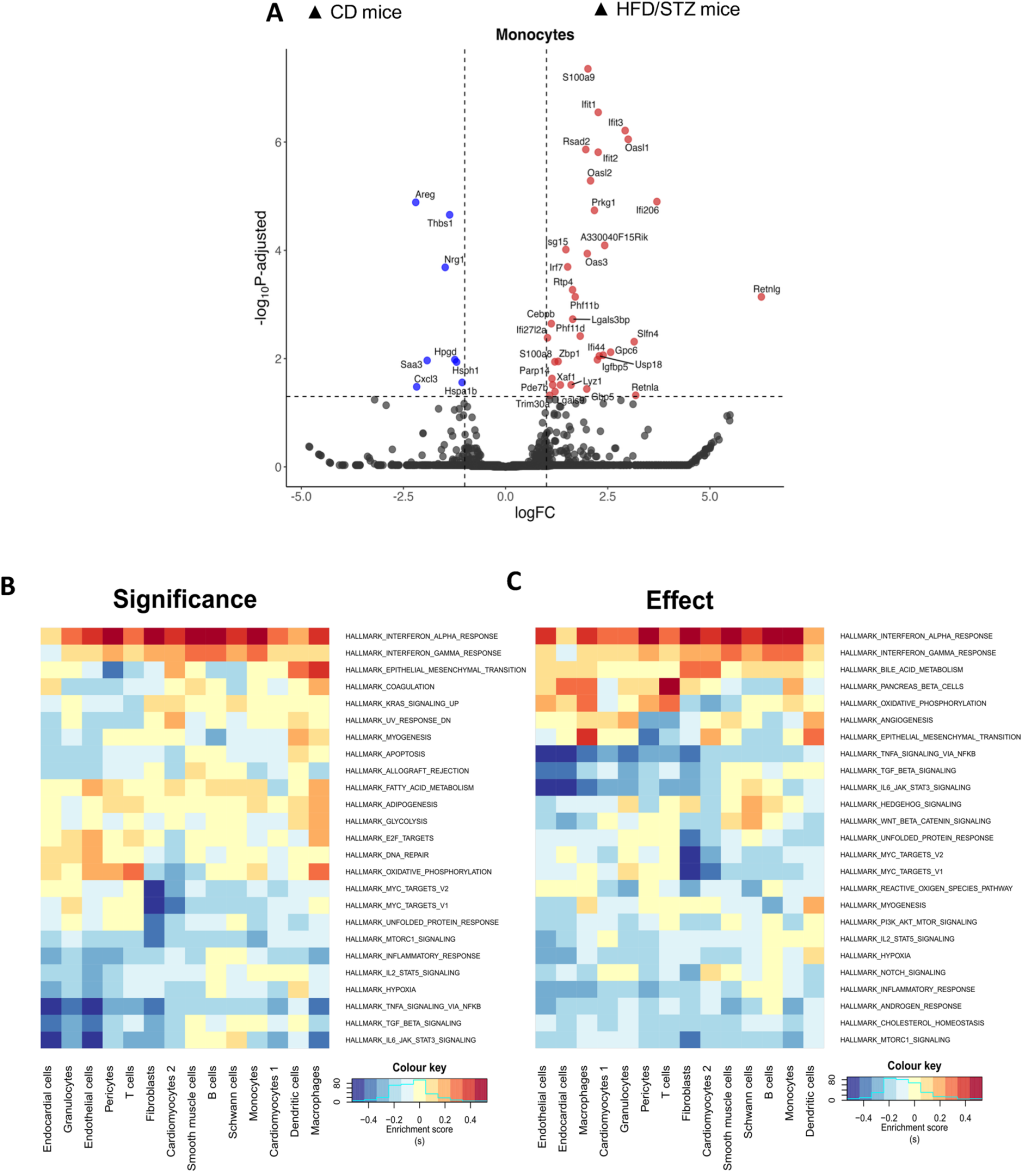
Signature of cardiac inflammatory markers of DbCM progression. Single-nuclei RNA sequencing analysis of LV tissue from DbCM and CD mice for **A** Differential gene expression of monocyte population, and **B**, **C** Pathway analysis using Mitch package with ranking by significance and effect (magnitude). *CD* control diet, *HFD/STZ* high fat diet/streptozotocin

To study pathogenic drivers of adverse structural and functional remodelling, LV tissue from HFD/STZ and control mice at 6 months of study were subjected to snRNA-seq to decipher underlying mechanisms related to DbCM progression at the cellular level. The snRNA-seq revealed various cell populations in mouse LV tissue. After quality control, we obtained 29,077 cells from three CD mice (21,246 cells) and three HFD/STZ mice (7,831 cells) for downstream analysis. Clustering and annotation revealed all major cell populations in the dataset (Fig. 5A, B and Figure S1), with the top three most specific genes for each cell population presented in Fig. 5C. Composition analysis was performed to study the impact of diabetes upon cell abundance (Fig. 5D). Monocytes significantly increased in the diabetic heart, compared to control mice (P-value = 0.0092; Fig. 5E), suggesting enhanced cardiac inflammation and immune cell infiltration associated with DbCM development. Otherwise, cardiac endothelial cells tended towards decrease in HFD/STZ mice, whilst fibroblast numbers appeared to be increased compared to CD mice (Fig. 5E). These findings suggest that diabetes could alter cell abundance within the myocardium. Differential gene expression analysis of monocytes revealed that those from HFD/STZ mice exhibited activated type I IFN signature and predominantly expressed genes such as *Ifit1*, *Ifit2*, *Ifit3, Isg15*, and *Irf7*, compared to CD mice (Fig. 6A)*.* Moreover, *S100a8* (P-value = 2.23 × 10^–05^) and *S100a9* (P-value = 2.70 × 10^–12^)*,* which are involved in inflammatory mediation and migration [31], were also considerably induced in monocytes in HFD/STZ mice vs CD mice (Fig. 6A). Differential gene expression with FDR < 0.05 and log2foldchange > 1 across all cell populations is presented in Supplement Table S3.

To gain biological insight into activated pathways in DbCM mice, we used a multi-contrast gene set enrichment (Mitch) analysis. “Pseudobulk” differential expression (DE) tables from edgeR for each cell population were inputted, and differential gene activity was scored based on the default setting. The DE profiles of each cell population were considered as an independent contrast. Then, Mitch analysis was performed using hallmark gene sets of *Mus musculus* from the MSigDB database [24]. The hallmark gene sets were differentially regulated (FDR MANOVA < 0.05). We prioritised the results by significance and effect (magnitude) (Fig. 6B, C). The findings showed that interferon-α response was upregulated in the diabetic heart condition, whether ranked by significance or effect. Notably, the monocyte population was among the important divers in inflammatory responses (Fig. 6B, C). Apart from monocytes, the interferon-α response was also involved across cell populations, indicating the involvement of systemic-level response in DbCM progression (Fig. 6B, C). To assess the systemic impact of diabetes-induced pathogenesis of DbCM, we employed an unbiased approach to detect changes in the plasma proteome using untargeted proteomics analysis (DIA-PASEF). This allows us to understand natural inflammation responses at the systemic level during disease progression.

### Plasma proteomics indicates elevated C-reactive protein in DbCM mice

**Fig. 7:**
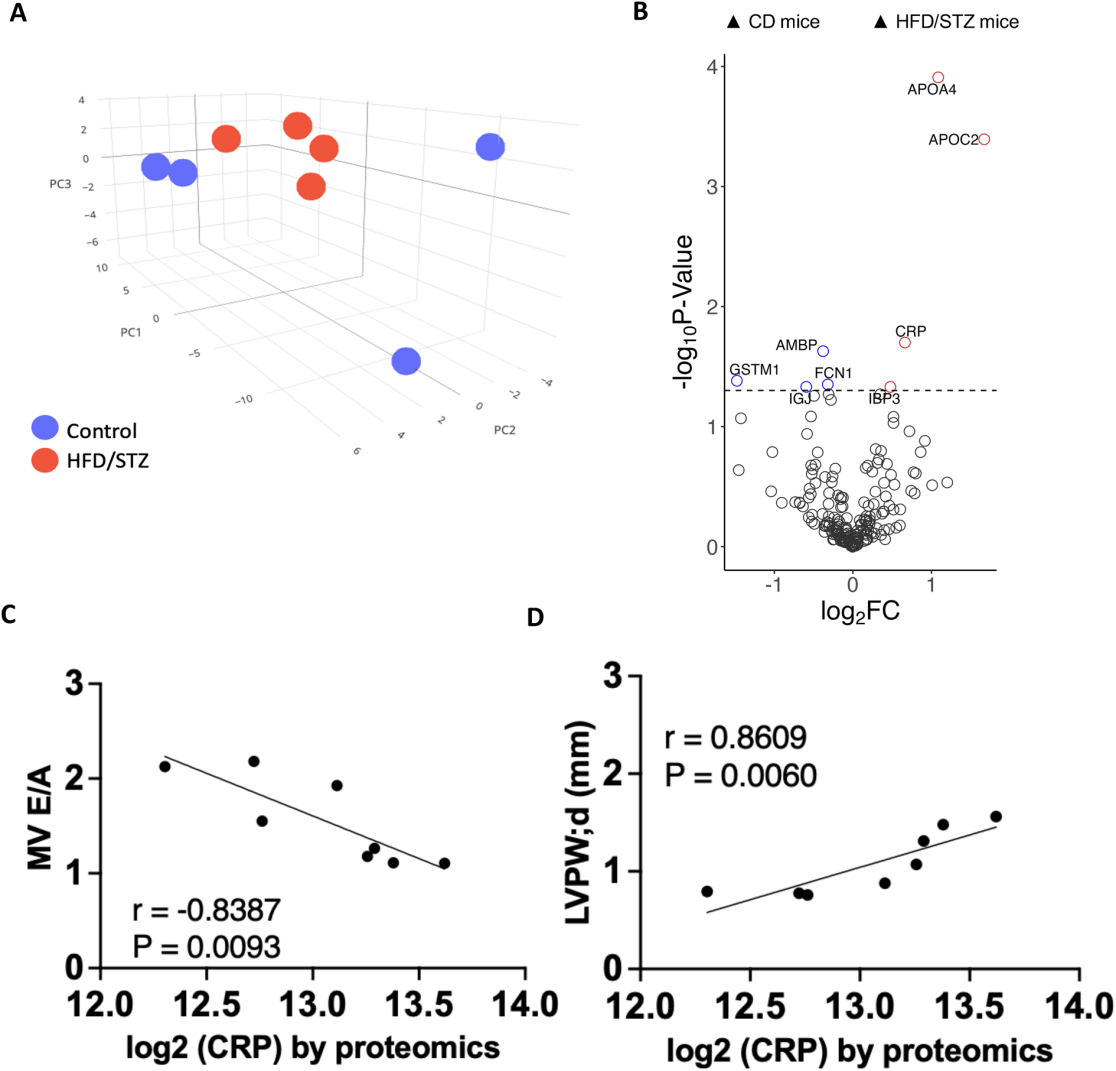
Plasma proteomic profiling using DIA-PASEF method. Mass spectrometry proteomics analysis of plasma from DbCM and CD mice at 6 months. **A** Principal component analysis (PCA) analysis across sample groups, **B** Differential protein expression, **C** Correlation between plasma C-reactive protein and MV E/A ratio, and **D** Correlation between plasma C-reactive protein and left ventricular posterior wall during diastolic phase (LVPW;d). *CD* control diet, *HFD/STZ* high fat diet/streptozotocin, *CRP* C-reactive protein, *MV E/A ratio* early to late diastolic mitral flow velocity

Proteomics analysis was performed on plasma samples from both groups to define the signature of the circulating proteome in DbCM mice. The results demonstrated that plasma proteome profiles in HFD/STZ mice were clearly distinct from those of CD mice (Fig. 7A). C-reactive protein (CRP) was among the most upregulated proteins in the plasma of HFD/STZ mice, compared to CD mice, consistent with a DbCM-induced inflammatory state (Fig. 7B). Moreover, increased plasma CRP levels were significantly correlated with worsening MV E/A ratio (r = − 0.8387, P = 0.0093) and positively correlated with LV posterior wall thickness during diastole (r = 0.8609, P = 0.0060) (Fig. 7C, D). Taken together, these findings indicate that HFD/STZ mice exhibit systemic inflammation and may be linked with specific induction of interferon-α response and elevated plasma CRP level, which are reflective of progressive diastolic dysfunction and cardiac remodelling.

## Discussion

In clinical studies, evaluation of cardiac abnormalities related to DM is often assessed at a single time point, limiting ability to comprehensively track the influence of DM on cardiac structure and function throughout its progression. In the current study, we phenotyped and characterised a modified mouse model of T2DM induced by long-term HFD and single-dose STZ to promote the reliable development of DbCM. Our findings demonstrate that this HFD/STZ-induced DbCM model effectively replicates clinical features of DbCM, specifically, chronic hyperglycaemia, insulin resistance, progressive diastolic dysfunction with maintained ejection fraction and blood pressure, and adverse LV remodelling linked with systemic and myocardial inflammation. Notably, these characteristics of DbCM serve as reliable structural and functional markers, which are commonly found in the early stage of natural history of HF development, indicating predisposition to cardiac stress and advance to symptomatic HF stages [2, 32, 33].

It can be challenging to model the cardiac phenotype of DM patients in the absence of other risk factors, such as hypertension, coronary artery disease, and atherosclerosis. Robust pre-clinical models that mimic the complexities of DbCM progression in humans are required to support the generation of reliable clinically relevant data, which is a fundamental requirement for effective translational research. In this regard, our modified HFD/STZ-induced DbCM model exhibits T2DM, reflected by hyperglycaemia, insulin resistance, and reduced insulin sensitivity, in parallel with progressive LV diastolic dysfunction with preserved ejection fraction and blood pressure. Indeed, insulin resistance is central to the development of T2DM and plays a significant role in the “vicious circle” between T2DM and HF [34]. In patients with diabetes, LV diastolic dysfunction is specifically associated with elevated fasting blood glucose, HbA1c levels, and body mass index (BMI), all of which are indicators of insulin resistance [35]. LV diastolic dysfunction is evident in many T2DM patients, even with optimal metabolic control, and may occur in the presence or absence of clinically detectable heart disease [36]. Notably, the severity of LV diastolic dysfunction in T2DM is linked to the extent of glucose dysregulation related to insulin resistance, leading to an elevated risk of incident HF and cardiovascular mortality in individuals with T2DM [2, 33, 36, 37]. In fact, LV diastolic dysfunction is reported to be predictive of HF development over a 6-year follow-up period in a population-based cohort [38].

DbCM is also characterised by adverse LV remodelling, particularly disproportionate myocardial fibrosis together with cardiac hypertrophy as prevalent structural features underlying associated myocardial stiffness and compromised cardiac function [39]. Diabetes-induced cardiac remodelling is driven by various complex mechanisms, including hyperglycaemia, oxidative stress, and inflammation, both at a systemic level and within the myocardium [40, 41]. There is growing evidence supporting the role of diverse pathophysiological mechanisms in DbCM, offering new opportunities for therapeutic intervention. Several review studies have highlighted the multifactorial nature of DbCM progression, reinforcing the potential for targeted treatment strategies [42, 43]. Currently, therapeutic options for DbCM remain limited beyond conventional risk factor management, and further in-depth investigation is clearly warranted.

Ongoing preclinical and clinical studies have proposed innovative therapeutic strategies targeting key pathological pathways of DbCM, including impaired cardiac metabolism (e.g., AT-001), oxidative stress (e.g., mito-TEMPO), ferroptosis (e.g., Ferrostatin-1), mitochondrial homeostasis (e.g., arginine supplementation), myocardial fibrosis and remodelling (e.g., FT23), and inflammation (e.g., MCC950) [43–45]. More recently, SGLT2 inhibitors have been shown to mitigate cardiomyocyte senescence in DbCM by modulating the FOXO1–ANGPTL4 axis [46].

In addition, several natural compounds and pharmacological agents, including sarpogrelate [47], oleuropein [48], adipsin [49], and β-caryophyllene [50], have demonstrated potential in attenuating DbCM-related pathological changes. However, these interventions have yet to be translated into established clinical practice, raising concerns about the current challenges in translational research. Given the incomplete understanding of underlying DbCM mechanisms, there is a pressing need for the development of robust preclinical models that accurately recapitulate human DbCM, thereby advancing translational studies and therapeutic development. Taken together, an advantage of pre-clinical models is the ability to easily and accurately assess cardiac dysfunction at both early and established stages of LV remodelling in diabetes before progression to HF or end-stage clinical disease, enabling the investigation of innovative diagnostic and preventive therapeutic strategies.

Notably, our modified model of progressive DbCM was characterised by specific activation of clinically relevant inflammation processes, including increased monocytes into the myocardium, increased *S100a8/9* expression, and elevated plasma CRP levels. Indeed, myocardial inflammation is implicated as one of the key drivers in the progression of clinical DbCM [51, 52], with our snRNA-seq analysis identifying monocyte trafficking into hearts of diabetic mice as a defining feature, together with enhanced expression of *S100A8/9,* which stimulates inflammatory cytokine production and human monocyte migration [31, 53]. In line with this, S100a9 − / − mice demonstrate diminished migratory capacity in response to chemokines [54]. To emphasise inflammation-driven DbCM progression, our present study underlines the role of circulating CRP level correlated with progressive diastolic dysfunction and LV hypertrophy. Overexpression of CRP in diabetic mice was shown to exacerbate LV dysfunction and remodelling [55]. Translating to humans, evidence would suggest that measurement of CRP could support risk prediction of DbCM in individuals with T2DM [56, 57]. Likewise, the Framingham Heart Study revealed that elevated baseline levels of TNF-α, IL-6, and CRP in individuals without a history of acute myocardial infarction (MI) are associated with a significantly increased long-term risk of developing HF, irrespective of MI occurrence [58]. Moreover, other studies emphasised that elevated CRP and IL-6 levels are associated with an increased risk of HF in individuals with obesity and metabolic syndrome [59, 60]. Consistent with these data, our HFD/STZ-induced DbCM model highlights that inflammatory markers are importantly associated with DbCM progression, and these markers demonstrate the potential clinical applicability. Taken together, our modified model of experimental DbCM, induced by the combination of HFD and STZ, recapitulates multiple metabolic, structural, functional, and inflammatory features of clinical disease progression, thereby mimicking complex interplay amongst these processes as an important foundation for robust and reliable translational research.

Whilst our presented data provide strong support for the clinical relevance of our HFD/STZ-induced DbCM model, this study has several limitations which should be considered in the design for future hypothesis-led investigation. Firstly, we chose to focus on male mice for the initial characterisation of our HFD/STZ-induced DbCM model to overcome established challenges associated with the use of female mice for diabetes research, including resistance to STZ-induced β-cell dysfunction [61]. There are known differences between women and men with HF in terms of risk factors, underlying pathophysiology, clinical presentation, treatment responses, and outcomes [62, 63]. In clinical studies, the Framingham Heart Study showed that diabetes increases HF risk more in women than men, with a 5.1-fold and 2.4-fold increase, respectively, compared to non-diabetic patients [64]. Supporting the presence of sex differences in DbCM, the recent phase 3 ARISE-HF trial, which evaluated the effects of AT-001 in patients with DbCM, highlighted sex-specific baseline characteristics—such as higher NT-proBNP levels, more preserved LV function, and greater symptomatic burden in women compared to men [65]. Therefore, sex-specific mechanisms governing cardiac remodelling in DbCM need to be explored in future studies. It is clearly important to develop alternate protocols for reliable induction of experimental DM and DbCM in female mice to support the investigation of sex differences in the context of DbCM progression, which are widely reported in patients [65]. Secondly, our HFD/STZ-induced DbCM model may only be suitable for the study of diabetes-induced cardiac remodelling exhibiting an inflammatory component, although we note that this represents a central aspect of the clinical DbCM. In this regard, our model may be particularly relevant for the investigation of residual HF risk associated with inflammation-driven cardiac remodelling. However, this study did not perform causal validation of Type I IFN signalling; future work using the IFN-α/β receptor (IFNAR) blockade or genetic loss-of-function models will be necessary to establish causality linking inflammatory drivers and DbCM development. Thirdly, complementary models are required to support the study of other contributors to DbCM progression, such as mitochondrial alteration, impaired Ca^2+^ handling, and lipotoxicity. Fourthly, our snRNA-seq analysis shows the underrepresentation of cardiomyocyte nuclei, likely due to technical challenges in isolating large, fragile, and multinucleated cardiomyocytes [18, 66, 67]. This known bias may affect the interpretation of cardiomyocyte-specific signals and compositions. In addition, detailed myocardial multimodality imaging in DbCM may allow for early detection of subclinical structural and functional changes in the diabetic heart [68], so it warrants future investigation. Finally, we specifically used the C57BL/6J mouse strain in this study due to its well-documented susceptibility to diet-induced cardiac remodelling and consistent manifestation of the HFpEF phenotype [69, 70]. These characteristics underscore a suitable and widely accepted model for investigating metabolic cardiomyopathy, particularly in the context of HFD/STZ induction [71]. To enhance the generalisability and reproducibility of our findings, future studies utilising the HFD/STZ-induced DbCM model in non-C57BL/6J mouse strains are warranted. Further investigations would help to delineate strain-specific effects and validate whether the observed cardiac phenotypes and molecular alterations are broadly applicable across different genetic backgrounds.

Beyond the limitations of our study that must be addressed to enhance DbCM diagnosis and treatments, this study accordingly paves the way for future direction in DbCM research. Given the involvement of IFN signalling in DbCM pathogenesis, future studies should systematically evaluate IFN-targeted therapies to assess their therapeutic potential in modulating inflammation and improving cardiac remodelling. Developing IFN-targeted therapies in DbCM may face several key challenges, including the heterogeneity of IFN responses, the potential for off-target effects, and the difficulty in accurately measuring IFN activity [72]. Thus, balancing between cardiac effects and side effect profile using IFN-targeted therapies in DbCM treatment is paramount [73]. Regarding the chronic nature of DbCM development, longer-term studies using the HFD/STZ-induced DbCM model are warranted to evaluate the durability of cardiac remodelling, progression toward HF phenotypes and potential impacts on survival and systemic metabolic dysfunction in the HFD/STZ-induced DbCM model. Furthermore, specific diagnostic markers for DbCM are useful for screening individuals with T2DM, preventing DbCM development [74]. Both identified gene and protein related to DbCM in our study may serve as potential candidates for early screening of DbCM development. Although the sample size used for snRNA-seq is comparable to previous studies [19, 75, 76], our analysis in HFD/STZ and CD mice (n = 3 per group) is underpowered due to the lower number of nuclei for robust statistical comparisons based on a priori power analysis, but these findings present an opportunity to develop a reliable HFD/STZ-induced DbCM model. Therefore, future studies incorporating longitudinal snRNA-seq at multiple time points, across both sexes, will be important to capture cell type–specific dynamics underlying disease progression, and will require larger sample sizes to ensure robust statistical power.

In summary, animal models play a crucial role in advancing the understanding of diabetes complications and can provide particularly valuable insights into the mechanisms driving initiation and progression of DbCM. However, design of rodent models which faithfully recapitulate human DbCM progression is challenging due to disease complexity and characteristic subclinical remodelling. It is, therefore, critical to employ animal models that accurately reflect metabolic alterations and exhibit early DbCM development and progressive disease, to support the discovery of clinically relevant targets for translation. In this regard, the modified DbCM mouse model reported in the present study induced through a combination of HFD and STZ provides a “triple-hit” reflection of a clinical disease characterised by (1) T2DM with insulin resistance, (2) progressive diastolic dysfunction and cardiac remodelling, and 3) metabolic and myocardial inflammation. As such, this model represents an improved approach to robust induction of experimental DbCM, which accurately recapitulates the clinical phenotype, so it is evidently suitable to support reliable translational investigation of DbCM progression from early subclinical dysfunction to established remodelling and advanced HF.

## Supplementary Information

Additional file 1.

## Data Availability

The data that support the findings of this study are available from the corresponding author upon request.
