# Supplementary material for: Deep phenotyping of a modified diabetic cardiomyopathy mouse model which reflects clinical disease progression
Source: Diabetol Metab Syndr. 2025 Aug 14;17:334. doi: 10.1186/s13098-025-01913-3 (PMC12351925; doi:10.1186/s13098-025-01913-3)
Supplement: Supplementary file 1 — Additional file 1. [file 13098_2025_1913_MOESM1_ESM.docx]

**Supplement for**

**Deep phenotyping of a modified diabetic cardiomyopathy mouse model which reflects clinical disease progression**

**Authors**

Narainrit Karuna^1,2^, Lauren Kerrigan^1^, Kevin Edgar^1^, Oisin Cappa^1^,
David Simpson^1^, Claire Tonry^1^, David J Grieve^1^, Chris J Watson^1^

^1^Wellcome-Wolfson Institute for Experimental Medicine, Queen’s University Belfast, Belfast, United Kingdom

^2^Department of Pharmaceutical Care, Faculty of Pharmacy, Chiang Mai University, Chiang Mai, Thailand

**Corresponding author:** Chris J Watson, [chris.watson@qub.ac.uk](mailto:chris.watson@qub.ac.uk)

Wellcome-Wolfson Institute for Experimental Medicine, Queen’s University Belfast, Belfast, Northern Ireland

**Expanded Materials & Methods section**

Sample preparation for single-nuclei RNA analysis

Data processing for single-nuclei RNA analysis

Sample preparation for proteomics analysis

Mass spectrometry

Spectral library generation in FragPipe

DIA software configuration and dia-PASEF data processing

**Supplement Table and Figure**

Table 1 Primer sequences

Table 2 Cell marker genes

Table 3 Differential gene expression in each cell population

Figure 1 Clustering and cell markers

Figure 2 A priori power analysis for snRNA-seq and proteomics analysis

**Expanded Materials & Methods section**

**Sample preparation for single-nuclei RNA analysis**

Ventricles from CD mice (n=3) and HFD/STZ mice (n=3) were processed following a previously established protocol [1]. Library preparation and nuclei sequencing were carried out at the Genomics Core Facility of Queen’s University Belfast. Nuclei suspensions were applied to the 10X Genomics platform, aiming to recover between 5,000 and 10,000 nuclei per sample. Single-nucleus RNA sequencing (snRNA-seq) libraries were prepared using the Chromium Single Cell 3′ v2 Reagent Kit (10X Genomics), and sequencing was conducted on the Illumina NovaSeq 6000 system.

**Data processing for single-nuclei RNA analysis**

Pre-processing of snRNA-seq data adhered to established quality control protocols to ensure the retention of high-quality nuclei for downstream analysis. Cells expressing fewer than 200 genes were excluded to eliminate low-quality or dying cells. Genes detected in fewer than three cells were removed to reduce noise from low-abundance transcripts. Additionally, nuclei in which over 20% of the total transcript count was attributed to mitochondrial genes were discarded. Subsequently, doublets were detected and removed using Scrublet package [2], while contamination from ambient RNA was corrected using DecontX package [3], which estimates and subtracts background expression levels from each nucleus base on Bayesian method.

Following quality control, gene expression data were normalised and log-transformed to stabilise variance. Highly variable genes across the dataset were identified for use in downstream analyses. Dimensionality reduction was achieved via principal component analysis (PCA), and clustering of nuclei was conducted using a k-nearest neighbors graph-based method implemented with the Leiden algorithm [4], which optimises modularity to define transcriptionally distinct clusters. Annotation of major cell populations was identified by expression of canonical marker genes derived from previously published studies [1, 5-7] , with full marker lists presented in Supplementary Table S2 and visualised in Supplementary Figure S1. Differential gene expression analysis between identified cell populations was performed using the non-parametric Wilcoxon rank-sum test to detect cell-type-specific marker genes. To assess group-level transcriptional changes between experimental conditions (HFD/STZ vs CD) within each cell population, we employed a pseudobulk differential expression strategy using the edgeR package[8], aggregating counts from individual nuclei per sample to approximate bulk-like comparisons. Genes with a false discovery rate (FDR) < 0.05 and absolute log2 fold change > 1 were considered significantly differentially expressed. All Data analysis carried out using Scanpy (version 1.9.6) [9], running on Python (version 3.11) or R (version 4.3.2) upon package compatibility.

To assess changes in cellular composition between experimental groups, we quantified the relative proportions of identified cell populations and performed statistical analysis using the speckle R package [10]. Prior to comparison, cell proportion data were transformed using the arcsine square root transformation to meet the assumptions of parametric testing. Transformed cell-type proportions were then compared between the HFD/STZ and CD groups to identify significant compositional changes.

To investigate the biological significance of transcriptional alterations, we conducted multi-contrast gene set enrichment analysis using the Mitch framework [11]. This method applies a rank-based multivariate analysis of variance (rank-MANOVA) approach, allowing for simultaneous assessment of pathway-level gene expression shifts across multiple contrasts—specifically, across different cell populations between conditions. This approach enhances detection power for pathway-level effects that may show coordinated or divergent trends across cell types. Gene set enrichment was performed using the Molecular Signatures Database (MSigDB) [12], specifically utilising the Hallmark gene sets curated for *Mus musculus*. These hallmark sets represent well-defined biological processes and signalling pathways, enabling interpretable insights into cell type–specific and global transcriptomic responses in the HFD/STZ-induced DbCM model. Results from the Mitch analysis allowed us to identify pathways dysregulated across multiple cell types, as well as those exhibiting cell type–restricted patterns of regulation.

**Sample preparation** **for proteomics analysis**

Plasma from control diet mice (control; n=4) and DbCM mice (HFD/STZ; n=4) were used in proteomics analysis. Plasma samples were depleted with High-Select™ Top 14 Abundant Protein Depletion Resin (A36368, Thermo Scientific™) to remove the 14 most abundant proteins from plasma. Plasma proteins (50 µg) were denatured and reduced in 50 mM ammonium bicarbonate buffer, pH 8.0, 8 M urea. Then, 5 ul of 100 mM dithiothreitol (DTT) was added and incubated at 27°C for 1 hour. Protein cysteines were alkylated with 5 µl of 140 mM iodoacetamide (OAA) for 60 minutes at room temperature in the dark. Proteins were enzymatically cleaved overnight by adding equal amounts of trypsin in a 1:50 (%w/w) enzyme: protein ratio. De-salting and purification of peptides were performed on C18 Stage Tips. Purified peptides were vacuum-centrifuged to dryness and reconstituted in 0.1 % Formic Acid (FA), stored at -20ºC until analysis.

**Mass spectrometry**

Peptide concentrations were measured by DeNovix®, and 200 ng peptides were loaded onto Evotips for LC/MS analysis. The Evotips were washed with 0.1% FA/99.9% Acetonitrile (ACN), equilibrated with 0.1% FA, loaded with the sample dissolved in 0.1% FA, and washed with 0.1% FA. The Evosep One LC system coupled with a timsTOF Pro mass spectrometer (Bruker, Germany) was used to measure all samples. The 30 samples per day protocol was applied, and the peptides were separated on a reversed-phase C18 PEPSEP column (15cm x 100μm ID, C18, 3 μm). The library samples were acquired in Data Dependent Acquisition (DDA) mode and PASEF (Parallel Accumulation Serial Fragmentation) modes enabled. For Data Independent Acquisition (DIA), a diaPASEF scheme consisting of 34 precursor isolation windows of 26 Da width with a mass overlap of 1 Da, covering a mass range of 350 – 1200 m/z and an ion mobility range of 0.6 to 1.6 Vs/cm, was created using the Bruker timsControl interface (2.0.53).

**Spectral library generation in FragPipe**

We used FragPipe computational platform (version 21.1)[13] with MSFragger (version 4.0)[14] to build spectral libraries. Raw files (.d) from library samples were used for generating spectral libraries based on default workflow for simple conventional (closed) searches. Protein sequence database of *Mus musculus* ([UP000000589](https://www.uniprot.org/proteomes/UP000000589)) from UniProt (downloaded on December 22, 2023) was obtained.

**DIA software configuration and DIA-PASEF data processing**

DIA-NN (version 1.8.1)[15] with default setting and maximum mass accuracy tolerances set to 15 ppm for both MS1 and MS2 spectra were used. DDA-based spectral libraries were used as input, protein inference was disabled, and MBR was enabled. All other settings were left default. The precursor FDR threshold was set to 5% for all DIA-NN analyses. The Protein.Group column in DIA-NN’s report was used to identify the protein group.

Pre-processing of DIA-NN output file was performed. Unique protein groups were checked and log2 transformation was applied. Next, any proteins containing missing values more than 50% of samples were filter outed, leading to 201 unique proteins. Data normalisation was carried out using cyclic loess from limma package.[16] All missing values were imputed based on these proteins with low abundance using a manually defined left-shifted Gaussian distribution (shift = 1.8, scale = 0.3). Principal component analysis was performed. To compare two groups, a t-test was carried out, and P<0.05 was considered as significant protein expressions. Benjamini and Hochberg (BH) method was used for adjusted P.

**Supplement Table and Figure**

**Table 1 Primer sequences**

| **Genes** | **Forward** | **Reverse** |
| --- | --- | --- |
| *Col1a1* | CGATGGATTCCCGTTCGAGT | CGATCTCGTTGGATCCCTGG |
| *Col3a1* | CCAAGGGTGCTACTGGACTC | GCTCACCCTTGTTACCGGAT |
| *B2m* | TCACACTGAATTCACCCCCA | TCACATGTCTCGATCCCAGT |

**Table 2 Cell marker genes**

| **Cell type** | **Markers** |
| --- | --- |
| B cells | *Ms4a1*, *Cd79a*, *Cd79b* |
| Cardiomyocytes | *Myh6*, *Tnnt2*, *Ttn* |
| Dendritic cells | *Naaa* |
| Endocardial cells | *Npr3* |
| Endothelial cells | *Cdh5*, *Pecam1*, *Emcn* |
| Fibroblasts | *Col1a1*, *Pdgfra* |
| Granulocytes | *Ccr1*, *S100a8*, *S100a9* |
| Macrophages | *Adgre1*, *Mrc1* |
| Monocytes | *Plac8*, *Itgal* |
| Pericytes | *Kcnj8*, *Vtn* |
| Schwann cells | *Plp1*, *Mbp*, *S100b* |
| Smooth muscle cells | *Tagln*, *Acta2*, *Myh11* |
| T cells | *Cd3e*, *Cd5*, *Lef1* |

**Table 3 Differential gene expression in each cell population**

| **B cells** | | | | | | |
| --- | --- | --- | --- | --- | --- | --- |
| **gene** | **logFC** | **logCPM** | | **F** | **pval** | **padj** |
| Jchain | -3.0521224 | 7.46027208 | | 91.1081663 | 1.40E-21 | 2.38E-17 |
| S100a8 | 3.37822799 | 5.64196765 | | 57.504576 | 3.41E-14 | 2.90E-10 |
| Ifit2 | 2.32892675 | 6.36857986 | | 52.8469039 | 3.64E-13 | 2.06E-09 |
| S100a9 | 3.1344106 | 5.64089878 | | 51.3904661 | 7.65E-13 | 3.25E-09 |
| Irf7 | 2.0043064 | 6.67900263 | | 44.4897085 | 2.58E-11 | 8.75E-08 |
| Igha | -1.8509213 | 8.35161034 | | 41.963625 | 9.36E-11 | 2.65E-07 |
| Mt1 | -1.8537726 | 7.13275841 | | 39.5809556 | 3.17E-10 | 7.68E-07 |
| Zbtb16 | -2.4817068 | 5.93296678 | | 38.6146017 | 5.19E-10 | 1.10E-06 |
| Ifi44 | 2.62020022 | 5.36314805 | | 37.0812553 | 1.14E-09 | 2.15E-06 |
| Ifi27l2a | 1.69465307 | 9.02560342 | | 36.566537 | 1.48E-09 | 2.52E-06 |
| Ifit3 | 2.69417289 | 5.1413796 | | 33.9770681 | 5.60E-09 | 8.65E-06 |
| Oasl1 | 2.43723422 | 5.22583466 | | 30.4133643 | 3.50E-08 | 4.96E-05 |
| Cmpk2 | 2.46551213 | 5.25326193 | | 29.3355613 | 6.11E-08 | 7.98E-05 |
| Cebpb | 1.782843 | 6.2224078 | | 28.9439653 | 7.47E-08 | 9.07E-05 |
| Icam1 | -1.4589558 | 7.64582744 | | 26.4787687 | 2.67E-07 | 0.00030249 |
| Pycard | 1.85649043 | 5.74440267 | | 26.2252898 | 3.05E-07 | 0.00032334 |
| Rsad2 | 1.40633988 | 7.55273293 | | 25.322204 | 4.86E-07 | 0.00048581 |
| Vcam1 | -2.0994478 | 5.4913046 | | 24.6174019 | 7.01E-07 | 0.00066122 |
| Foxp2 | 4.14475754 | 4.05036176 | | 23.2484226 | 1.43E-06 | 0.00127521 |
| Adamts4 | -1.5455506 | 6.29943887 | | 22.5697199 | 2.03E-06 | 0.00172355 |
| S100a6 | 1.3287394 | 8.40936234 | | 22.4252779 | 2.19E-06 | 0.00172355 |
| Csmd1 | -4.5349114 | 4.16605393 | | 22.3297227 | 2.30E-06 | 0.00172355 |
| Ifit1 | 1.36113249 | 6.66298006 | | 22.3019633 | 2.33E-06 | 0.00172355 |
| Isg15 | 1.25027992 | 7.9634367 | | 20.8974844 | 4.85E-06 | 0.00343449 |
| Acta1 | -3.3628732 | 4.40743578 | | 20.7034079 | 5.37E-06 | 0.0035558 |
| Ifit3b | 3.38295781 | 4.14680318 | | 20.6776496 | 5.44E-06 | 0.0035558 |
| Hsph1 | -1.2374295 | 8.46078228 | | 20.1480724 | 7.18E-06 | 0.00451533 |
| Stip1 | -1.3133513 | 6.66289366 | | 19.769454 | 8.75E-06 | 0.00530724 |
| Cadm2 | -6.2492342 | 3.70977596 | | 18.9780666 | 1.32E-05 | 0.00775452 |
| Cxcl1 | -1.3614687 | 6.35178885 | | 18.2060288 | 1.99E-05 | 0.01053512 |
| A730063M14Rik | 4.74340893 | 3.56894419 | | 18.2168345 | 1.97E-05 | 0.01053512 |
| Xaf1 | 1.37241003 | 6.18748888 | | 18.2922776 | 1.90E-05 | 0.01053512 |
| Ism1 | 2.7795484 | 4.25497683 | | 17.6328997 | 2.68E-05 | 0.01380452 |
| Ifi206 | 2.01782645 | 4.92424973 | | 17.4763788 | 2.91E-05 | 0.01454775 |
| Ddx60 | 1.94616843 | 4.97643718 | | 17.1471678 | 3.46E-05 | 0.01680452 |
| Igfbp5 | 1.73510041 | 5.19875953 | | 16.8351957 | 4.08E-05 | 0.01925458 |
| Btnl9 | 1.10407155 | 7.31402271 | | 15.9969856 | 6.35E-05 | 0.02765476 |
| H1f0 | 1.54596131 | 5.53384223 | | 16.011667 | 6.30E-05 | 0.02765476 |
| Socs1 | 1.32104502 | 5.91138039 | | 16.049605 | 6.18E-05 | 0.02765476 |
| **Cardiomyocytes1** | | | | | | |
| **gene** | **logFC** | **logCPM** | | **F** | **pval** | **padj** |
| Acta1 | -2.9006407 | 9.7481951 | | 44.2657292 | 2.90E-11 | 3.77E-07 |
| Ankrd1 | -1.8030646 | 10.2210888 | | 23.2518691 | 1.43E-06 | 0.00929037 |
| Ifit1 | 4.18656257 | 6.20368396 | | 20.3298972 | 6.53E-06 | 0.02838631 |
| **Cardiomyocytes2** | | | | | | |
| **gene** | **logFC** | **logCPM** | | **F** | **pval** | **padj** |
| Cxcl2 | -2.9916458 | | 7.73746567 | 72.7573056 | 1.49E-17 | 2.61E-13 |
| Ifi27l2a | 2.11348566 | | 8.51772367 | 55.5836029 | 9.06E-14 | 7.90E-10 |
| Zbtb16 | -2.4678416 | | 7.85996954 | 52.8400717 | 3.66E-13 | 2.13E-09 |
| Cxcl14 | 2.69119945 | | 5.85381407 | 45.2116955 | 1.78E-11 | 7.77E-08 |
| Ifit1 | 1.9255336 | | 7.49519558 | 42.1022173 | 8.72E-11 | 3.04E-07 |
| Clec3b | 1.90056783 | | 7.08222074 | 36.70768 | 1.38E-09 | 4.01E-06 |
| C3 | 1.80655925 | | 7.22191375 | 35.7770546 | 2.22E-09 | 5.48E-06 |
| Ifit3 | 2.31836816 | | 6.05474749 | 35.5395458 | 2.51E-09 | 5.48E-06 |
| Meg3 | 2.07531504 | | 6.31126004 | 34.9376912 | 3.42E-09 | 6.63E-06 |
| Irf7 | 1.85260501 | | 7.03937169 | 33.5746614 | 6.89E-09 | 1.20E-05 |
| Abca8a | 1.91427791 | | 6.74615473 | 33.283757 | 8.00E-09 | 1.27E-05 |
| Tmem252 | -4.9718169 | | 5.0418727 | 33.1087661 | 8.75E-09 | 1.27E-05 |
| Ccl8 | 3.51333219 | | 4.91721621 | 32.9290316 | 9.60E-09 | 1.29E-05 |
| Timp4 | -1.8794485 | | 7.78523277 | 31.0152742 | 2.57E-08 | 3.20E-05 |
| Myh11 | -2.3189297 | | 6.44716284 | 29.747209 | 4.94E-08 | 5.75E-05 |
| C1qa | -4.1799503 | | 5.07035365 | 29.4448481 | 5.77E-08 | 6.29E-05 |
| Smoc2 | 1.61667772 | | 7.25030605 | 28.5315295 | 9.25E-08 | 9.49E-05 |
| Ifit2 | 1.81454414 | | 6.57582631 | 28.2320313 | 1.08E-07 | 0.00010463 |
| Acta2 | -1.9127218 | | 7.08180812 | 28.0627213 | 1.18E-07 | 0.00010819 |
| Cacybp | -1.8323826 | | 7.22090153 | 27.0914961 | 1.95E-07 | 0.00016169 |
| Adamts5 | 1.67776844 | | 6.9437157 | 27.1602607 | 1.88E-07 | 0.00016169 |
| Hsd11b1 | 1.94709345 | | 6.08846572 | 26.8944031 | 2.15E-07 | 0.00016445 |
| Myh7 | -2.517682 | | 5.85606086 | 26.882943 | 2.17E-07 | 0.00016445 |
| Bicc1 | 1.69922467 | | 6.76283483 | 26.3828137 | 2.81E-07 | 0.00019153 |
| Ccl7 | 1.58453945 | | 6.87341635 | 26.4943843 | 2.65E-07 | 0.00019153 |
| Rnd1 | -1.6332662 | | 7.74813029 | 26.351303 | 2.85E-07 | 0.00019153 |
| Col3a1 | 1.4978979 | | 7.56808477 | 26.1418342 | 3.18E-07 | 0.00019822 |
| Tagln | -1.966163 | | 6.65356149 | 26.1638405 | 3.14E-07 | 0.00019822 |
| Serping1 | 1.46901897 | | 7.69454173 | 25.9638372 | 3.49E-07 | 0.00020986 |
| Gfra1 | 2.64323406 | | 5.01559136 | 25.3727165 | 4.74E-07 | 0.00027554 |
| Plxdc2 | 1.68173272 | | 6.52309343 | 24.9174518 | 6.00E-07 | 0.00033762 |
| Procr | -6.389078 | | 4.43877012 | 23.9701415 | 9.81E-07 | 0.0005347 |
| Ifi44 | 1.73970947 | | 6.31805777 | 23.3176833 | 1.38E-06 | 0.00072769 |
| Dpep1 | 1.58825348 | | 6.70536358 | 23.0710488 | 1.56E-06 | 0.0008029 |
| Ebf2 | 1.93375145 | | 5.81331569 | 22.9069965 | 1.70E-06 | 0.00084941 |
| Pf4 | -1.8337077 | | 6.46872867 | 22.021111 | 2.70E-06 | 0.00130411 |
| Hsph1 | -1.3364835 | | 9.01584553 | 21.976481 | 2.77E-06 | 0.00130411 |
| Pdk4 | 2.12315759 | | 5.30702318 | 21.7578618 | 3.10E-06 | 0.00138539 |
| Lyz2 | -2.0254335 | | 6.12237409 | 21.7979633 | 3.03E-06 | 0.00138539 |
| Fndc1 | 2.56873525 | | 4.86253726 | 21.7108032 | 3.18E-06 | 0.00138539 |
| Htra3 | 1.57839945 | | 6.49454576 | 21.6160679 | 3.34E-06 | 0.00138619 |
| Stambpl1 | 3.65969072 | | 4.22641004 | 21.6473684 | 3.28E-06 | 0.00138619 |
| Ahsa1 | -1.7896094 | | 6.55877273 | 21.465383 | 3.61E-06 | 0.00146459 |
| Ogn | 1.36893557 | | 7.41460267 | 21.0798501 | 4.41E-06 | 0.00175007 |
| Apod | 2.70222462 | | 4.69658521 | 20.8576939 | 4.96E-06 | 0.0019215 |
| Lgals4 | 1.50511561 | | 6.46779402 | 20.7617078 | 5.21E-06 | 0.00193426 |
| Ddit4 | -1.9227051 | | 6.24055532 | 20.7877606 | 5.14E-06 | 0.00193426 |
| Rsad2 | 1.29175545 | | 8.05019986 | 20.6615045 | 5.49E-06 | 0.00199568 |
| Abi3bp | 1.8526849 | | 5.56267521 | 20.1467269 | 7.18E-06 | 0.00255814 |
| Oasl2 | 1.63215979 | | 6.16553618 | 19.9677887 | 7.89E-06 | 0.0027528 |
| Prelp | 1.57608336 | | 6.25210615 | 19.3097162 | 1.11E-05 | 0.00380809 |
| Pcdh9 | 1.56216414 | | 6.26979627 | 19.2401181 | 1.15E-05 | 0.00387348 |
| C1s1 | 1.99032854 | | 5.30779875 | 19.1696221 | 1.20E-05 | 0.00394332 |
| Col6a2 | 1.61366858 | | 6.02367025 | 18.9065583 | 1.37E-05 | 0.00444219 |
| Aldh1a1 | 2.16720543 | | 5.05324455 | 18.7548555 | 1.49E-05 | 0.00472238 |
| Crispld2 | 1.35946775 | | 6.92474515 | 18.6047014 | 1.61E-05 | 0.00501796 |
| Cygb | 1.41818412 | | 6.63653297 | 18.5439577 | 1.66E-05 | 0.0050895 |
| Itgb6 | 1.76193994 | | 5.65794873 | 18.4088336 | 1.78E-05 | 0.00534282 |
| Scn7a | 1.82348188 | | 5.56271081 | 18.3535942 | 1.84E-05 | 0.00534282 |
| C1qtnf7 | 2.15958951 | | 5.05963305 | 18.3660705 | 1.83E-05 | 0.00534282 |
| Hspe1 | -1.245828 | | 8.49334743 | 18.2726109 | 1.92E-05 | 0.00548338 |
| Rtl8a | -1.9326244 | | 5.95726333 | 18.1905462 | 2.00E-05 | 0.0056324 |
| Nuak2 | -2.638298 | | 5.06143744 | 17.9642769 | 2.25E-05 | 0.00624227 |
| Inmt | 3.85147918 | | 3.93155654 | 17.8996222 | 2.33E-05 | 0.00630305 |
| Angptl4 | 1.87139499 | | 5.47221031 | 17.8863321 | 2.35E-05 | 0.00630305 |
| Lpar1 | 1.95579856 | | 5.21932605 | 17.7048722 | 2.58E-05 | 0.00682856 |
| Stip1 | -1.5058223 | | 6.70494201 | 17.6696514 | 2.63E-05 | 0.00685232 |
| Ifih1 | 1.38154281 | | 6.74598328 | 17.4619615 | 2.93E-05 | 0.00753054 |
| Col5a3 | 1.66198089 | | 5.69720333 | 17.4219123 | 3.00E-05 | 0.00757937 |
| Sox17 | -1.3926179 | | 7.17960782 | 17.3143277 | 3.17E-05 | 0.00781204 |
| Ucp3 | 3.76264551 | | 3.91964238 | 17.3101147 | 3.18E-05 | 0.00781204 |
| Islr | 1.44684231 | | 6.41355416 | 17.0756092 | 3.60E-05 | 0.00871542 |
| Lrp1 | 1.37406752 | | 6.48710004 | 16.7316687 | 4.31E-05 | 0.01030316 |
| Tbc1d4 | 1.40577101 | | 6.28343636 | 16.6446144 | 4.51E-05 | 0.01064109 |
| S100a8 | -5.849198 | | 4.03691398 | 16.6012639 | 4.62E-05 | 0.01074188 |
| Rarres2 | 1.26501556 | | 6.95650275 | 16.5341207 | 4.78E-05 | 0.01098249 |
| Gas1 | 1.49528869 | | 6.05202553 | 16.3921384 | 5.16E-05 | 0.01168265 |
| Pdgfra | 1.5348108 | | 5.83752327 | 16.3260813 | 5.34E-05 | 0.01194183 |
| Pla1a | 1.73151207 | | 5.4672415 | 16.2559378 | 5.54E-05 | 0.0122352 |
| Ccl2 | -1.2803564 | | 7.27715198 | 16.231479 | 5.61E-05 | 0.01223921 |
| Col6a1 | 1.42663561 | | 6.17964369 | 16.1153824 | 5.97E-05 | 0.01285198 |
| Spon2 | 2.27405052 | | 4.70783635 | 15.9671482 | 6.45E-05 | 0.01372882 |
| Chordc1 | -1.4360539 | | 6.77771695 | 15.9188592 | 6.62E-05 | 0.0139138 |
| Lepr | 5.36812592 | | 3.38365609 | 15.8453525 | 6.88E-05 | 0.01429252 |
| Ptgds | -2.2821601 | | 5.20704871 | 15.7824485 | 7.11E-05 | 0.01443193 |
| Vwa8 | 1.30092204 | | 6.65394992 | 15.7975505 | 7.06E-05 | 0.01443193 |
| G0s2 | 1.61220203 | | 5.61777409 | 15.6690381 | 7.55E-05 | 0.01514732 |
| Zbp1 | 3.36417654 | | 3.94303875 | 15.5505872 | 8.04E-05 | 0.01594304 |
| Sh3bgrl2 | -2.0704467 | | 5.40601027 | 15.5121158 | 8.21E-05 | 0.01608792 |
| Ptpn3 | 1.98655707 | | 4.95419787 | 15.4650793 | 8.41E-05 | 0.01613071 |
| Lum | 1.14101246 | | 7.50758903 | 15.485933 | 8.32E-05 | 0.01613071 |
| Ier3 | -1.1302789 | | 9.40223851 | 15.3726169 | 8.83E-05 | 0.01675528 |
| Naaladl2 | 1.90671106 | | 5.11135186 | 15.3519335 | 8.93E-05 | 0.01675752 |
| Ccl21a | 2.48164426 | | 4.48440567 | 15.2497244 | 9.43E-05 | 0.0175008 |
| Ms4a4d | 1.55088906 | | 5.76057545 | 15.1709996 | 9.83E-05 | 0.01805356 |
| Grb14 | 1.35412708 | | 6.23726446 | 15.1050891 | 0.00010178 | 0.01836649 |
| Gadd45b | -1.1313509 | | 8.44001239 | 15.0992043 | 0.0001021 | 0.01836649 |
| Snhg18 | 2.16024041 | | 4.76450991 | 15.0159476 | 0.0001067 | 0.01899873 |
| Igsf10 | 3.26617355 | | 3.92772249 | 14.898808 | 0.00011354 | 0.01938698 |
| S100a9 | -3.939579 | | 4.25909019 | 14.9356677 | 0.00011134 | 0.01938698 |
| Plac8 | 5.26059462 | | 3.32773984 | 14.902537 | 0.00011331 | 0.01938698 |
| Gfpt2 | 1.23102536 | | 6.84904578 | 14.9571113 | 0.00011008 | 0.01938698 |
| Thbs2 | 2.11240384 | | 4.82091294 | 14.8838605 | 0.00011444 | 0.01938698 |
| Depp1 | -1.4278897 | | 6.74426148 | 14.8525873 | 0.00011635 | 0.01952149 |
| Apba2 | 5.29885141 | | 3.30361101 | 14.7833362 | 0.0001207 | 0.02005861 |
| Tnfaip6 | 1.19221451 | | 6.79599271 | 14.6203393 | 0.0001316 | 0.02146095 |
| Abca8b | 1.86571584 | | 5.0755927 | 14.6306456 | 0.00013088 | 0.02146095 |
| Rnf150 | 1.51401251 | | 5.61296585 | 14.5055439 | 0.00013987 | 0.02259762 |
| Ifit3b | 2.13967663 | | 4.80237819 | 14.4541811 | 0.00014373 | 0.02300911 |
| Angptl2 | 1.72728754 | | 5.22030386 | 14.3439963 | 0.00015239 | 0.02384664 |
| C1qb | -2.7867087 | | 4.70864455 | 14.3357041 | 0.00015306 | 0.02384664 |
| Gm15564 | -1.5632392 | | 6.14813863 | 14.3626454 | 0.00015089 | 0.02384664 |
| Ces1d | 1.83117997 | | 5.08963332 | 14.2595457 | 0.00015938 | 0.02461127 |
| Arhgap6 | 1.31331142 | | 6.33615894 | 14.2226445 | 0.00016254 | 0.02487834 |
| Hspd1 | -1.0995753 | | 8.16669081 | 13.9948813 | 0.00018346 | 0.02747297 |
| Ifi207 | 1.17065822 | | 6.95185941 | 13.9839813 | 0.00018452 | 0.02747297 |
| Nus1 | -1.3179637 | | 6.79393631 | 14.0163403 | 0.00018137 | 0.02747297 |
| Qpct | 2.6578495 | | 4.22226679 | 13.9711264 | 0.00018579 | 0.02747297 |
| Kcnq4 | 3.49845346 | | 3.75864397 | 13.9467575 | 0.00018821 | 0.02759745 |
| Clmp | 2.12280037 | | 4.70525066 | 13.8991321 | 0.00019304 | 0.0280696 |
| Adgrd1 | 2.16469514 | | 4.61605843 | 13.8344551 | 0.0001998 | 0.02881215 |
| Vipr2 | 5.17999685 | | 3.24270783 | 13.7684341 | 0.00020694 | 0.02959779 |
| Hspa1b | -1.0470003 | | 9.67113699 | 13.6862209 | 0.0002162 | 0.03067044 |
| Sfrp1 | 1.70985012 | | 5.21420629 | 13.5800316 | 0.00022878 | 0.03219301 |
| Ggt5 | 2.43483548 | | 4.37389497 | 13.5401441 | 0.00023369 | 0.03236235 |
| P3h4 | 2.02888675 | | 4.79628837 | 13.5462872 | 0.00023293 | 0.03236235 |
| Col6a3 | 1.40391664 | | 5.8071416 | 13.4738757 | 0.00024209 | 0.0332614 |
| Gxylt2 | 2.49994579 | | 4.31478374 | 13.3571693 | 0.00025763 | 0.03511955 |
| Lgr6 | 1.81301867 | | 4.99118889 | 13.3387986 | 0.00026016 | 0.03516003 |
| Dnaja1 | -1.0678142 | | 10.8405976 | 13.2972965 | 0.00026598 | 0.03516003 |
| Egfr | 1.35262116 | | 6.0239506 | 13.3103039 | 0.00026414 | 0.03516003 |
| Gm11290 | -1.2561552 | | 6.94791776 | 13.3171973 | 0.00026317 | 0.03516003 |
| Rnase4 | 1.26332794 | | 6.34929565 | 13.281896 | 0.00026818 | 0.03518343 |
| Ehd1 | -1.13506 | | 7.52045232 | 13.2053776 | 0.00027935 | 0.03637559 |
| Mt2 | -1.0341336 | | 8.1817785 | 13.1158398 | 0.00029302 | 0.03787297 |
| Gramd1b | 1.45783629 | | 5.62968082 | 13.0943082 | 0.0002964 | 0.038029 |
| Fign | 1.80751883 | | 4.99429831 | 12.9393596 | 0.00032197 | 0.04012861 |
| Ccnd1 | -1.0520049 | | 8.45676594 | 12.9454007 | 0.00032093 | 0.04012861 |
| Cpq | 1.14381061 | | 6.80065623 | 12.9395146 | 0.00032194 | 0.04012861 |
| Fgf12 | 5.01466711 | | 3.20826117 | 12.9561537 | 0.00031909 | 0.04012861 |
| Pcolce2 | 1.37852468 | | 5.82476876 | 12.9253617 | 0.00032438 | 0.04014301 |
| Cdkn2c | 2.12476787 | | 4.61425839 | 12.8891097 | 0.00033073 | 0.04042058 |
| Sdc2 | 1.23682233 | | 6.37617844 | 12.8860946 | 0.00033126 | 0.04042058 |
| Ctla2b | -1.7393721 | | 5.56028861 | 12.8549903 | 0.00033681 | 0.04053102 |
| Mmp14 | 1.6390619 | | 5.28661924 | 12.8594132 | 0.00033602 | 0.04053102 |
| Col1a2 | 1.08532467 | | 7.11305944 | 12.8069579 | 0.00034557 | 0.04101898 |
| Icam1 | -1.0391454 | | 8.52963928 | 12.8137062 | 0.00034432 | 0.04101898 |
| Gja1 | -1.0428363 | | 8.09445457 | 12.7886051 | 0.00034897 | 0.0411434 |
| Hspb1 | -1.0978398 | | 12.1797796 | 12.6406847 | 0.00037769 | 0.04423042 |
| Pla2r1 | 2.87040035 | | 3.95361543 | 12.4898079 | 0.00040944 | 0.0476291 |
| Hspa8 | -1.0577214 | | 11.5641845 | 12.4570578 | 0.00041668 | 0.04815036 |
| Entpd2 | 1.3276387 | | 5.95800869 | 12.4407025 | 0.00042035 | 0.0482542 |
| Six4 | 4.96135365 | | 3.16145227 | 12.4233162 | 0.00042428 | 0.0483871 |
| Gm10076 | -1.0734421 | | 8.47197224 | 12.3786878 | 0.00043454 | 0.04923544 |
| **Dendritic cells** | | | | | | |
| **gene** | **logFC** | | **logCPM** | **F** | **pval** | **padj** |
| Retnla | 4.84380149 | | 7.42995425 | 55.4968361 | 9.49E-14 | 1.35E-09 |
| Dcn | 2.74731923 | | 8.17650661 | 35.3937455 | 2.71E-09 | 1.93E-05 |
| Ccl12 | 3.20950597 | | 7.39733409 | 33.6360006 | 6.68E-09 | 3.17E-05 |
| Il10 | -3.845494 | | 6.3494052 | 27.3033508 | 1.75E-07 | 0.00062177 |
| Gsn | 2.07578209 | | 8.94063796 | 24.8666748 | 6.16E-07 | 0.00175616 |
| Fn1 | 3.35906623 | | 6.24090225 | 19.5471606 | 9.83E-06 | 0.02335445 |
| Ccl5 | -2.5885023 | | 6.54547514 | 18.7525722 | 1.49E-05 | 0.03034979 |
| Rhoj | 2.49018737 | | 6.81947575 | 18.0473396 | 2.16E-05 | 0.03844581 |
| **Endocardial cells** | | | | | | |
| **gene** | **logFC** | | **logCPM** | **F** | **pval** | **padj** |
| Ifi27l2a | 4.13700847 | | 9.44713148 | 372.270505 | 9.91E-83 | 1.71E-78 |
| Lsamp | 4.30543823 | | 7.9861761 | 284.811074 | 9.02E-64 | 7.79E-60 |
| Cxcl2 | -3.8972022 | | 8.80851488 | 270.58539 | 1.10E-60 | 6.36E-57 |
| Cxcl1 | -2.7681586 | | 9.06361469 | 173.131975 | 1.71E-39 | 7.38E-36 |
| Postn | 3.11727265 | | 6.33217146 | 107.98956 | 2.82E-25 | 9.75E-22 |
| Nppc | 6.03878646 | | 4.96527083 | 106.023369 | 7.59E-25 | 2.19E-21 |
| Selp | -1.7779931 | | 9.5281028 | 86.3874972 | 1.52E-20 | 3.75E-17 |
| Zfp46 | -2.5955573 | | 6.77639269 | 82.4958704 | 1.09E-19 | 2.35E-16 |
| Icam1 | -1.6075848 | | 10.046082 | 78.8458076 | 6.87E-19 | 1.32E-15 |
| Csf1 | -1.8309676 | | 8.59943848 | 76.4580406 | 2.30E-18 | 3.97E-15 |
| Tnip1 | -2.19452 | | 7.18297903 | 73.1973803 | 1.20E-17 | 1.78E-14 |
| Ccl2 | -2.0925773 | | 7.52127796 | 73.1288523 | 1.24E-17 | 1.78E-14 |
| Hspb1 | -1.3073469 | | 12.131182 | 71.0644986 | 3.52E-17 | 4.68E-14 |
| Rbfox1 | 3.72534652 | | 4.98775457 | 66.6111859 | 3.36E-16 | 4.15E-13 |
| Hamp | 3.47702504 | | 5.06356189 | 65.3315412 | 6.43E-16 | 7.41E-13 |
| Vcam1 | -1.3920435 | | 10.5481087 | 64.3262875 | 1.07E-15 | 1.16E-12 |
| Mt1 | -1.7398006 | | 8.11220753 | 64.0319748 | 1.24E-15 | 1.26E-12 |
| Cxcl9 | -1.5717029 | | 8.93100621 | 62.8456586 | 2.27E-15 | 2.18E-12 |
| Hspa1a | -1.3178735 | | 11.0417758 | 62.61446 | 2.55E-15 | 2.32E-12 |
| Foxp2 | 3.76391596 | | 4.75440751 | 59.1500897 | 1.48E-14 | 1.28E-11 |
| Nfkbia | -1.2603028 | | 10.8976787 | 56.0909158 | 7.00E-14 | 5.76E-11 |
| Nckap5 | 2.01590211 | | 6.38336101 | 55.2412274 | 1.08E-13 | 8.47E-11 |
| Igfbp5 | 1.33643171 | | 9.89901178 | 54.6687851 | 1.44E-13 | 1.08E-10 |
| Noct | -1.7077168 | | 7.6421346 | 54.4776915 | 1.59E-13 | 1.14E-10 |
| Sele | -1.4873732 | | 8.82561576 | 54.0594588 | 1.97E-13 | 1.36E-10 |
| Zbtb16 | -2.026373 | | 6.63750147 | 52.5255433 | 4.29E-13 | 2.85E-10 |
| Hsph1 | -1.4192985 | | 8.90367951 | 51.6093367 | 6.84E-13 | 4.38E-10 |
| Enpp2 | 2.19920835 | | 6.05267721 | 51.4888069 | 7.27E-13 | 4.49E-10 |
| Wnt9b | 2.83082766 | | 5.13027758 | 51.1028321 | 8.85E-13 | 5.28E-10 |
| Hspa1b | -1.335111 | | 9.45943713 | 50.7044899 | 1.08E-12 | 6.25E-10 |
| Ankrd33b | -1.5969118 | | 7.76551015 | 50.2537368 | 1.36E-12 | 7.60E-10 |
| Hsp90aa1 | -1.1617688 | | 11.0587382 | 49.038404 | 2.53E-12 | 1.37E-09 |
| Inhba | 2.10849221 | | 5.90243192 | 48.6018923 | 3.16E-12 | 1.66E-09 |
| Irf7 | 1.56449611 | | 7.27207289 | 46.8266588 | 7.82E-12 | 3.98E-09 |
| Csf3 | -2.3165984 | | 5.93636452 | 46.2823119 | 1.03E-11 | 5.10E-09 |
| Ifi27l2b | 2.99708156 | | 4.78026851 | 43.7193873 | 3.82E-11 | 1.83E-08 |
| Dnaja1 | -1.1056678 | | 10.78138 | 42.9167253 | 5.75E-11 | 2.69E-08 |
| Il1r1 | -1.4365166 | | 7.76770554 | 40.2234358 | 2.28E-10 | 1.04E-07 |
| Timp4 | -1.4065145 | | 7.76015864 | 39.4327973 | 3.42E-10 | 1.51E-07 |
| Cacybp | -1.5769435 | | 6.969049 | 39.3156797 | 3.63E-10 | 1.53E-07 |
| Ptprt | 6.78113935 | | 3.53809791 | 39.3244845 | 3.61E-10 | 1.53E-07 |
| Ier3 | -1.0743194 | | 10.4811577 | 38.8754944 | 4.54E-10 | 1.87E-07 |
| Hspd1 | -1.3841927 | | 7.79476942 | 38.7497652 | 4.85E-10 | 1.95E-07 |
| Rarres2 | 1.92153544 | | 5.79462181 | 37.635014 | 8.58E-10 | 3.37E-07 |
| Gja1 | -1.303522 | | 8.03846094 | 36.6469419 | 1.42E-09 | 5.47E-07 |
| Mast4 | -1.1802151 | | 8.91745722 | 35.7722923 | 2.23E-09 | 8.37E-07 |
| Mt2 | -1.9725484 | | 5.90215019 | 35.5395559 | 2.51E-09 | 9.24E-07 |
| Tnfsf9 | -1.6169903 | | 6.53047137 | 35.478564 | 2.59E-09 | 9.33E-07 |
| Cd24a | 1.49406463 | | 6.56850515 | 34.5215162 | 4.23E-09 | 1.49E-06 |
| Phlda1 | -1.3182025 | | 7.70994299 | 34.4064896 | 4.49E-09 | 1.55E-06 |
| Neurl3 | -1.3310675 | | 7.61631517 | 34.2795727 | 4.80E-09 | 1.61E-06 |
| AW112010 | -1.2075306 | | 8.37134154 | 34.2579354 | 4.85E-09 | 1.61E-06 |
| Ripk2 | -1.439141 | | 7.00592512 | 33.8726393 | 5.91E-09 | 1.93E-06 |
| Plaur | -1.2594167 | | 7.90949851 | 33.4234672 | 7.44E-09 | 2.38E-06 |
| Epha7 | 1.48135788 | | 6.47071036 | 32.2146341 | 1.39E-08 | 4.36E-06 |
| Rps6ka3 | -1.1565635 | | 8.51269677 | 32.1069619 | 1.46E-08 | 4.52E-06 |
| Ltbp1 | 1.28990694 | | 7.39694331 | 31.7394263 | 1.77E-08 | 5.37E-06 |
| C3 | 2.01232368 | | 5.2713902 | 31.2887546 | 2.23E-08 | 6.65E-06 |
| Fas | -1.323189 | | 7.23757083 | 30.9408598 | 2.67E-08 | 7.82E-06 |
| Pim3 | -1.0971908 | | 8.81511657 | 30.8697923 | 2.77E-08 | 7.98E-06 |
| Coch | 6.45488215 | | 3.32294353 | 30.7145641 | 3.00E-08 | 8.50E-06 |
| Gadd45b | -1.0953337 | | 8.91546047 | 30.6219791 | 3.15E-08 | 8.77E-06 |
| Rel | -1.3059054 | | 7.34137599 | 30.4802371 | 3.38E-08 | 9.29E-06 |
| Rnd1 | -1.0518949 | | 9.234903 | 30.4321504 | 3.47E-08 | 9.37E-06 |
| Dennd3 | -1.4064669 | | 6.81144112 | 29.9108363 | 4.54E-08 | 1.21E-05 |
| Prox1 | 1.63815037 | | 5.92281467 | 29.6720325 | 5.13E-08 | 1.34E-05 |
| Il6 | -1.2522894 | | 7.27945497 | 29.0867201 | 6.94E-08 | 1.76E-05 |
| Ifit3 | 1.23818011 | | 7.12632381 | 29.0177677 | 7.20E-08 | 1.80E-05 |
| Fn1 | 1.19447572 | | 7.63066304 | 28.5677329 | 9.08E-08 | 2.24E-05 |
| Zbtb46 | -1.3470852 | | 6.86749196 | 28.5291241 | 9.26E-08 | 2.25E-05 |
| Tcim | -1.0865258 | | 8.44760304 | 28.4414762 | 9.69E-08 | 2.33E-05 |
| Lgals3bp | 1.41753543 | | 6.2426475 | 28.1106306 | 1.15E-07 | 2.68E-05 |
| Ifi44 | 1.20815884 | | 7.16380642 | 28.0603964 | 1.18E-07 | 2.72E-05 |
| Ptpre | -1.3206747 | | 6.92092574 | 28.0190617 | 1.20E-07 | 2.74E-05 |
| Igfbp3 | -1.7558054 | | 5.81754884 | 27.9295943 | 1.26E-07 | 2.83E-05 |
| Arhgap26 | -1.1822915 | | 7.62127421 | 27.8117853 | 1.34E-07 | 2.93E-05 |
| Apol9a | 3.72964566 | | 3.75370702 | 26.6661592 | 2.42E-07 | 5.24E-05 |
| Pfkfb3 | -1.1952644 | | 7.36489687 | 26.5283264 | 2.60E-07 | 5.52E-05 |
| Flnb | -1.0039205 | | 8.99064774 | 26.5182776 | 2.62E-07 | 5.52E-05 |
| Ccdc141 | 1.40204954 | | 6.1541771 | 26.3711932 | 2.82E-07 | 5.88E-05 |
| Angptl2 | 2.37584031 | | 4.55158264 | 26.2796749 | 2.96E-07 | 6.09E-05 |
| Plscr1 | -1.1418262 | | 7.59119442 | 26.2241707 | 3.05E-07 | 6.20E-05 |
| Adamts4 | -1.0428413 | | 8.38896718 | 25.6989284 | 4.00E-07 | 8.04E-05 |
| Nrxn1 | 2.89814108 | | 4.13034848 | 25.4842721 | 4.47E-07 | 8.88E-05 |
| Clu | 1.02534054 | | 8.46269555 | 25.4350819 | 4.59E-07 | 9.01E-05 |
| Akap12 | -1.0005434 | | 8.71757963 | 25.1882879 | 5.21E-07 | 0.0001001 |
| Jak2 | -1.0461411 | | 8.22730851 | 24.8433786 | 6.23E-07 | 0.0001171 |
| Wnt4 | 2.60716522 | | 4.21640854 | 24.2449006 | 8.50E-07 | 0.00015634 |
| Uap1 | -1.112696 | | 7.52993062 | 24.0338357 | 9.49E-07 | 0.00016905 |
| Tmem252 | -2.0505727 | | 5.17710213 | 24.046186 | 9.43E-07 | 0.00016905 |
| Relb | -1.234269 | | 6.88930504 | 23.9554092 | 9.88E-07 | 0.00017428 |
| Fmnl2 | -1.0988782 | | 7.55238117 | 23.8396268 | 1.05E-06 | 0.00018137 |
| H19 | 1.02558494 | | 8.17208508 | 23.7659784 | 1.09E-06 | 0.00018658 |
| Stc1 | -1.3942861 | | 6.24809598 | 23.6880414 | 1.14E-06 | 0.00019238 |
| Birc3 | -1.1525515 | | 7.19212912 | 23.5332795 | 1.23E-06 | 0.00020647 |
| Olfml2a | 2.4400461 | | 4.33034436 | 23.3402776 | 1.36E-06 | 0.00022605 |
| Fgf14 | 1.42413601 | | 6.00773846 | 23.2225971 | 1.45E-06 | 0.00023802 |
| Hapln1 | 1.71386783 | | 5.18332584 | 23.1957366 | 1.47E-06 | 0.00023909 |
| Bcl3 | -1.1651385 | | 7.06268951 | 23.1000291 | 1.54E-06 | 0.00024894 |
| Clstn2 | 4.07245623 | | 3.47028641 | 22.970619 | 1.65E-06 | 0.0002638 |
| Ucp2 | 1.18061974 | | 6.6788129 | 22.6037192 | 1.99E-06 | 0.00031634 |
| Atp8b1 | -1.1254933 | | 7.1430921 | 22.4612276 | 2.15E-06 | 0.00033759 |
| Dkk2 | 1.72336882 | | 5.14966283 | 22.2706421 | 2.37E-06 | 0.00036944 |
| Rprml | 4.55688306 | | 3.31655069 | 21.9491748 | 2.80E-06 | 0.00043287 |
| Stip1 | -1.2401802 | | 6.57454113 | 21.7626629 | 3.09E-06 | 0.00047282 |
| Sox17 | -1.0694737 | | 7.34214289 | 21.6853827 | 3.22E-06 | 0.00048793 |
| Spats2 | -1.1011398 | | 7.16521798 | 21.5592808 | 3.44E-06 | 0.0005121 |
| Gm17268 | -2.3427004 | | 4.76625114 | 21.4298938 | 3.68E-06 | 0.00054056 |
| Serpine1 | -1.2374712 | | 6.50042796 | 21.2679068 | 4.00E-06 | 0.00058109 |
| Hif1a | -1.0377618 | | 7.58375259 | 21.2212285 | 4.10E-06 | 0.00058556 |
| Gbp5 | -1.0098723 | | 7.71827355 | 21.1321646 | 4.29E-06 | 0.00060838 |
| Ccdc85a | -1.0715411 | | 7.18127811 | 21.099946 | 4.37E-06 | 0.00061366 |
| Angptl4 | 1.76302661 | | 4.9986477 | 20.9696443 | 4.67E-06 | 0.00064633 |
| Olr1 | -1.2120253 | | 6.54521924 | 20.9207827 | 4.80E-06 | 0.00065776 |
| Fchsd2 | -1.130087 | | 6.90542584 | 20.4505321 | 6.13E-06 | 0.00083418 |
| Schip1 | -1.0282593 | | 7.41827083 | 20.2951019 | 6.65E-06 | 0.00089071 |
| Ddit4 | -1.4358274 | | 5.87279978 | 20.2975234 | 6.64E-06 | 0.00089071 |
| Ube2m | 1.25547776 | | 6.07332474 | 20.2425995 | 6.83E-06 | 0.00090844 |
| Grrp1 | -1.0695174 | | 7.02897482 | 19.7577434 | 8.80E-06 | 0.0011356 |
| Chordc1 | -1.1859023 | | 6.50694489 | 19.6807624 | 9.17E-06 | 0.00117351 |
| C4b | 4.39317641 | | 3.26694776 | 19.5499149 | 9.82E-06 | 0.00124742 |
| Tbx20 | 1.73052911 | | 4.89587934 | 19.3746891 | 1.08E-05 | 0.00134743 |
| Hand2 | 1.46401017 | | 5.48624844 | 19.2491132 | 1.15E-05 | 0.00142864 |
| Irak2 | -1.2727526 | | 6.17457693 | 19.1872495 | 1.19E-05 | 0.00145475 |
| Gcnt2 | -1.1006345 | | 6.79002012 | 18.9869165 | 1.32E-05 | 0.00160434 |
| Zc3h12a | -1.0656495 | | 6.9979131 | 18.8768599 | 1.40E-05 | 0.00168214 |
| Fst | 4.41093761 | | 3.21639114 | 18.8698597 | 1.40E-05 | 0.00168214 |
| Pip4k2a | -1.2293457 | | 6.25613483 | 18.6712719 | 1.56E-05 | 0.00182859 |
| Gm15283 | -1.8434157 | | 5.04697441 | 18.4080472 | 1.79E-05 | 0.00207113 |
| Gja4 | -1.3246016 | | 6.04836472 | 18.327889 | 1.86E-05 | 0.00213148 |
| Ifi206 | 3.07480694 | | 3.6292858 | 18.2358751 | 1.95E-05 | 0.00219099 |
| Slc10a6 | -1.2839842 | | 6.0160284 | 18.18994 | 2.00E-05 | 0.00221808 |
| Ppp1r14b | 1.25593941 | | 5.85180876 | 18.1284634 | 2.07E-05 | 0.00227624 |
| Apol9b | 2.62841507 | | 3.84530014 | 18.0897031 | 2.11E-05 | 0.00230834 |
| Gpc3 | 5.61985119 | | 2.98371791 | 18.0444552 | 2.16E-05 | 0.00234897 |
| Gm16685 | -3.9485067 | | 3.76429148 | 17.9605229 | 2.26E-05 | 0.00240937 |
| Baz1a | -1.1242805 | | 6.50194944 | 17.9670131 | 2.25E-05 | 0.00240937 |
| Efr3b | -1.1243281 | | 6.47716892 | 17.9320553 | 2.29E-05 | 0.00243066 |
| Syne3 | -1.922168 | | 4.89001319 | 17.7491819 | 2.52E-05 | 0.00262743 |
| Acer3 | -1.0124987 | | 7.00204781 | 17.6382633 | 2.67E-05 | 0.00276847 |
| Clec4g | 2.74432069 | | 3.73675533 | 17.4937371 | 2.89E-05 | 0.00288737 |
| Fkbp5 | -1.3595515 | | 5.75083243 | 17.3785595 | 3.07E-05 | 0.00304589 |
| Ahsa2 | -1.2607312 | | 5.96397655 | 17.073223 | 3.60E-05 | 0.00353616 |
| Ppard | -1.2065636 | | 6.14418728 | 17.0271125 | 3.69E-05 | 0.00360259 |
| Pik3r6 | -1.4959789 | | 5.42627288 | 16.9556081 | 3.83E-05 | 0.0037198 |
| Mmp14 | 1.85361078 | | 4.56301204 | 16.9396521 | 3.86E-05 | 0.00373023 |
| Stx11 | -1.0846195 | | 6.56596303 | 16.9108884 | 3.92E-05 | 0.00376613 |
| Muc16 | 5.52374623 | | 2.92469965 | 16.6811554 | 4.43E-05 | 0.00418101 |
| Serpinb9b | 2.82533314 | | 3.6310805 | 16.6428152 | 4.52E-05 | 0.00424317 |
| Sulf2 | 1.6103453 | | 4.87724118 | 16.6049764 | 4.61E-05 | 0.00428826 |
| Phlda3 | 1.04087243 | | 6.42295313 | 16.5204558 | 4.82E-05 | 0.00445328 |
| Bcl10 | -1.030251 | | 6.69919363 | 16.5041121 | 4.86E-05 | 0.00446793 |
| Gpx3 | 1.97874624 | | 4.29670712 | 16.2596551 | 5.53E-05 | 0.00502934 |
| Slc7a1 | -1.1156114 | | 6.32924453 | 16.1190975 | 5.95E-05 | 0.00528692 |
| Tmem100 | 1.48829877 | | 5.16104141 | 16.0531019 | 6.17E-05 | 0.00543697 |
| Gpat3 | -3.2217223 | | 3.87047795 | 16.0413239 | 6.20E-05 | 0.00544312 |
| Mir155hg | -1.2543647 | | 5.85709953 | 15.9938195 | 6.36E-05 | 0.00555319 |
| Enah | -1.1926265 | | 6.01592477 | 15.8290429 | 6.94E-05 | 0.00601598 |
| Edn1 | 1.12772674 | | 6.1037007 | 15.8044909 | 7.03E-05 | 0.0060457 |
| Rab20 | -1.33244 | | 5.70502046 | 15.7186764 | 7.36E-05 | 0.00626386 |
| Aebp1 | 1.34512095 | | 5.36716336 | 15.5184366 | 8.18E-05 | 0.00692924 |
| Tnnc1 | -1.0962323 | | 6.249262 | 15.4488903 | 8.48E-05 | 0.00715382 |
| Tnfaip3 | -1.0277334 | | 6.45504891 | 15.239407 | 9.48E-05 | 0.0078772 |
| Heatr5a | -1.3319169 | | 5.60357412 | 15.221488 | 9.57E-05 | 0.00791423 |
| Terb2 | 3.00109733 | | 3.44345492 | 15.0558721 | 0.00010447 | 0.00843393 |
| Mcf2l | -1.053596 | | 6.37680672 | 15.0479368 | 0.00010491 | 0.00843393 |
| Ltbp2 | 2.90763361 | | 3.49038815 | 14.8735895 | 0.00011507 | 0.00920736 |
| Ano1 | 2.89691901 | | 3.50174251 | 14.829703 | 0.00011777 | 0.00934942 |
| Hivep1 | -1.0454876 | | 6.36701324 | 14.8273178 | 0.00011792 | 0.00934942 |
| Cd14 | -1.882325 | | 4.63909657 | 14.6598002 | 0.00012888 | 0.01012503 |
| Vwa1 | -1.3447525 | | 5.51092887 | 14.582493 | 0.00013427 | 0.01050118 |
| Gramd1a | -1.0905771 | | 6.17960214 | 14.5138684 | 0.00013925 | 0.01079295 |
| Slc7a11 | 3.26326701 | | 3.31897499 | 14.4468136 | 0.0001443 | 0.01113407 |
| Upp1 | -1.1901371 | | 5.85511915 | 14.4091353 | 0.00014721 | 0.01130854 |
| Hdx | -1.8606372 | | 4.67843039 | 14.2506315 | 0.00016014 | 0.01213979 |
| Nbl1 | 1.0619703 | | 5.97814562 | 14.2008237 | 0.00016443 | 0.01235693 |
| Ralgds | -1.1249107 | | 5.96339002 | 14.0481398 | 0.00017833 | 0.01326289 |
| Pkhd1l1 | 1.15427102 | | 5.7222007 | 13.967197 | 0.00018618 | 0.01357762 |
| Tnfaip8l1 | -1.3405212 | | 5.44243785 | 13.9235739 | 0.00019055 | 0.01383793 |
| Nsg1 | 1.04659552 | | 6.05929156 | 13.8921147 | 0.00019376 | 0.01401257 |
| Gata4 | 1.26054434 | | 5.4087896 | 13.8388798 | 0.00019933 | 0.01435504 |
| Ddx60 | 1.18935734 | | 5.55567025 | 13.7503555 | 0.00020894 | 0.01487707 |
| Nod2 | -1.2475887 | | 5.60772823 | 13.7484143 | 0.00020916 | 0.01487707 |
| Ankrd1 | -1.3716675 | | 5.35648023 | 13.6447629 | 0.00022103 | 0.0155334 |
| Edil3 | 1.73193391 | | 4.34476562 | 13.4437101 | 0.00024601 | 0.01680668 |
| Col23a1 | 1.2005739 | | 5.61013654 | 13.4240247 | 0.00024861 | 0.01691706 |
| Megf6 | 1.34266562 | | 5.1579098 | 13.175939 | 0.00028377 | 0.01886423 |
| Tgfb2 | 2.14297963 | | 3.90001073 | 13.1139206 | 0.00029332 | 0.01927653 |
| Wnt2 | 1.86196405 | | 4.16544926 | 13.0331915 | 0.00030623 | 0.01997344 |
| Prkd1 | -1.0166766 | | 6.16578526 | 12.9360128 | 0.00032255 | 0.02087968 |
| Cst6 | 1.45415178 | | 4.79400059 | 12.9258702 | 0.0003243 | 0.02091476 |
| Adamts8 | 1.51280758 | | 4.71017389 | 12.8584514 | 0.00033619 | 0.02160114 |
| Camkk2 | -1.3538869 | | 5.32895739 | 12.8329759 | 0.0003408 | 0.02181608 |
| Gabpb1 | -1.077045 | | 5.97575169 | 12.702525 | 0.00036541 | 0.02313508 |
| Slc20a1 | -1.2557215 | | 5.50713123 | 12.6729602 | 0.00037123 | 0.02329454 |
| Ifit3b | 1.13763522 | | 5.53303215 | 12.6691909 | 0.00037198 | 0.02329454 |
| Pde4c | 1.497804 | | 4.76067382 | 12.656563 | 0.0003745 | 0.02336771 |
| Rrad | -1.2446931 | | 5.48734504 | 12.4735296 | 0.00041303 | 0.02549563 |
| Ttpal | -1.8336773 | | 4.47365365 | 12.4526904 | 0.00041766 | 0.02568986 |
| Pde7b | 1.23806705 | | 5.29377152 | 12.366238 | 0.00043745 | 0.0268115 |
| Inhbb | 1.57210643 | | 4.52981314 | 12.3519577 | 0.00044081 | 0.02682707 |
| Ccl4 | 1.95150747 | | 3.99113167 | 12.3565345 | 0.00043973 | 0.02682707 |
| Enho | 1.44608956 | | 4.76936183 | 12.3264337 | 0.00044687 | 0.02700613 |
| Zbp1 | 1.44175409 | | 4.74894074 | 12.2847999 | 0.00045695 | 0.02751897 |
| Bnip3 | -1.3881038 | | 5.161646 | 12.2277312 | 0.00047114 | 0.02807991 |
| Upk3b | 5.11641942 | | 2.72251292 | 12.1356989 | 0.00049496 | 0.02891436 |
| Msln | 5.11641942 | | 2.72251292 | 12.1356989 | 0.00049496 | 0.02891436 |
| Pin1 | 1.07156593 | | 5.70282621 | 12.0745876 | 0.00051145 | 0.02947576 |
| Thbs2 | 3.29044684 | | 3.17475526 | 12.0739943 | 0.00051161 | 0.02947576 |
| F830016B08Rik | -1.1444667 | | 5.67684147 | 12.0530296 | 0.0005174 | 0.02963588 |
| Acta2 | -1.0952021 | | 5.83284238 | 11.9879181 | 0.00053579 | 0.03046245 |
| Ccl19 | -5.3051733 | | 3.12944585 | 11.9468272 | 0.00054773 | 0.03093802 |
| Pdgfra | 1.81129166 | | 4.15454987 | 11.9229973 | 0.00055478 | 0.03123414 |
| Ptgs2os | -1.8702801 | | 4.40424026 | 11.8406125 | 0.00057987 | 0.03233055 |
| Tgtp1 | -1.4489264 | | 4.98834186 | 11.8008995 | 0.00059237 | 0.0328157 |
| Gfpt2 | -1.2392823 | | 5.35627952 | 11.7077814 | 0.00062275 | 0.03438869 |
| Dsg2 | -1.2562983 | | 5.41330727 | 11.6280897 | 0.00065 | 0.03566558 |
| P2ry2 | -1.9331569 | | 4.28524857 | 11.5546198 | 0.00067619 | 0.0368685 |
| St7 | 1.42732545 | | 4.66980679 | 11.4504838 | 0.00071515 | 0.03850673 |
| Gm3336 | 1.87082014 | | 4.00576194 | 11.391023 | 0.0007384 | 0.03939068 |
| Ch25h | -1.1486076 | | 5.56081248 | 11.3553302 | 0.00075273 | 0.04003126 |
| Hspa4l | -1.2018042 | | 5.46108032 | 11.3165821 | 0.0007686 | 0.04074978 |
| Ahnak2 | 1.21952904 | | 5.15110219 | 11.290027 | 0.00077967 | 0.04095976 |
| Anxa8 | 1.83347778 | | 4.00056657 | 11.2832708 | 0.00078251 | 0.04098449 |
| Sirt3 | 2.66098122 | | 3.35588952 | 11.2167063 | 0.00081108 | 0.04235235 |
| Nhs | -1.2272446 | | 5.32493256 | 11.1440442 | 0.00084346 | 0.04391093 |
| Gm45509 | -2.8870162 | | 3.61511154 | 11.1256657 | 0.00085186 | 0.04421492 |
| Cecr2 | -1.0814275 | | 5.68374158 | 11.0928171 | 0.00086708 | 0.04476078 |
| Tmem179b | 1.4055465 | | 4.68968554 | 11.0880516 | 0.00086931 | 0.04476078 |
| Stx6 | -1.03702 | | 5.85323877 | 11.0206831 | 0.00090147 | 0.04569233 |
| Lsr | 1.34386009 | | 4.80663402 | 11.0301891 | 0.00089686 | 0.04569233 |
| Uba7 | 1.16408418 | | 5.29723908 | 11.0231372 | 0.00090028 | 0.04569233 |
| Zhx2 | -1.0940489 | | 5.71036424 | 10.9885964 | 0.00091721 | 0.04595106 |
| Gm12703 | 1.42895349 | | 4.61937934 | 10.9752557 | 0.00092384 | 0.04601623 |
| Phldb1 | -1.0093744 | | 5.9023062 | 10.975969 | 0.00092348 | 0.04601623 |
| **Endothelial cells** | | | | | | |
| **gene** | **logFC** | | **logCPM** | **F** | **pval** | **padj** |
| Ifi27l2a | 2.66819673 | | 7.02807032 | 364.907689 | 3.51E-81 | 7.75E-77 |
| Igfbp5 | 2.47783782 | | 6.92789357 | 317.669026 | 6.21E-71 | 6.86E-67 |
| Irf7 | 2.14538738 | | 7.63302278 | 256.480729 | 1.21E-57 | 8.91E-54 |
| Zbtb16 | -2.2243194 | | 6.92568164 | 239.980904 | 4.68E-54 | 2.58E-50 |
| Ifit3 | 2.10091947 | | 6.58080801 | 227.362205 | 2.60E-51 | 1.15E-47 |
| Foxp2 | 3.41968303 | | 3.60457057 | 172.984887 | 1.80E-39 | 6.61E-36 |
| Ifit3b | 2.10710134 | | 4.97769041 | 163.86762 | 1.75E-37 | 5.51E-34 |
| S100a9 | -3.2441318 | | 4.17995242 | 155.009142 | 1.49E-35 | 4.12E-32 |
| Mt2 | -2.1502867 | | 5.2517359 | 148.829005 | 3.33E-34 | 8.17E-31 |
| Ifi44 | 1.65157507 | | 6.84379141 | 148.5526 | 3.83E-34 | 8.45E-31 |
| Cxcl2 | -1.5859335 | | 7.62840085 | 137.467185 | 1.01E-31 | 2.02E-28 |
| Mt1 | -1.5704382 | | 7.64285421 | 137.063126 | 1.23E-31 | 2.27E-28 |
| Prc1 | 1.94731121 | | 5.01195912 | 135.017231 | 3.45E-31 | 5.86E-28 |
| Hsph1 | -1.4612215 | | 9.14189765 | 132.347283 | 1.32E-30 | 2.08E-27 |
| Ifit2 | 1.49354 | | 7.37515433 | 128.197785 | 1.07E-29 | 1.57E-26 |
| Zbp1 | 2.38594648 | | 4.04881258 | 124.863154 | 5.70E-29 | 7.87E-26 |
| Cenpf | 1.6990783 | | 5.40923276 | 119.313467 | 9.32E-28 | 1.21E-24 |
| Ahsa2 | -1.6345912 | | 6.0214737 | 118.877548 | 1.16E-27 | 1.42E-24 |
| Ankrd33b | -1.4949817 | | 6.93038842 | 117.309784 | 2.55E-27 | 2.97E-24 |
| Adamts4 | -1.4305286 | | 7.88470953 | 117.174249 | 2.74E-27 | 2.98E-24 |
| Ifit1 | 1.40173539 | | 8.01169302 | 117.106174 | 2.83E-27 | 2.98E-24 |
| Cacybp | -1.4735989 | | 7.14362843 | 117.00842 | 2.97E-27 | 2.99E-24 |
| Top2a | 1.64963101 | | 5.42241683 | 114.002077 | 1.35E-26 | 1.30E-23 |
| Ube2c | 1.87817707 | | 4.79499219 | 113.487669 | 1.75E-26 | 1.61E-23 |
| Cxcl1 | -1.4161388 | | 7.74958601 | 113.156825 | 2.07E-26 | 1.83E-23 |
| Mki67 | 1.56368562 | | 5.44061665 | 102.915107 | 3.61E-24 | 3.06E-21 |
| Hspd1 | -1.3245502 | | 8.01919191 | 102.837331 | 3.75E-24 | 3.07E-21 |
| Ccl2 | -1.5874959 | | 5.5704148 | 101.002858 | 9.46E-24 | 7.46E-21 |
| Fgl2 | 1.72242555 | | 4.7557314 | 100.606033 | 1.16E-23 | 8.80E-21 |
| S100a8 | -2.7136701 | | 3.85473216 | 97.6217149 | 5.21E-23 | 3.83E-20 |
| Slc10a6 | -1.4453356 | | 5.99686625 | 94.0359903 | 3.18E-22 | 2.27E-19 |
| Cyp26b1 | 1.44591396 | | 5.49460504 | 93.9450658 | 3.33E-22 | 2.30E-19 |
| Cp | 1.50717405 | | 5.23456771 | 91.4580118 | 1.17E-21 | 7.59E-19 |
| Noct | -1.2620384 | | 7.74642685 | 91.4985029 | 1.14E-21 | 7.59E-19 |
| Sema3d | 2.2758463 | | 3.69126231 | 90.8019983 | 1.63E-21 | 1.03E-18 |
| Ifi213 | 1.6918739 | | 4.56852505 | 90.2346528 | 2.17E-21 | 1.33E-18 |
| Tnip1 | -1.3960177 | | 6.07834697 | 89.0934432 | 3.85E-21 | 2.24E-18 |
| Pkhd1l1 | 2.7938204 | | 3.01417279 | 89.1117269 | 3.82E-21 | 2.24E-18 |
| Chordc1 | -1.3115336 | | 6.66543477 | 88.7065047 | 4.68E-21 | 2.65E-18 |
| Ifi206 | 2.5445064 | | 3.30617203 | 88.5369211 | 5.10E-21 | 2.82E-18 |
| Rnd1 | -1.2192689 | | 8.02705665 | 87.276021 | 9.65E-21 | 5.20E-18 |
| Il6 | -1.4478279 | | 5.70822835 | 87.1423842 | 1.03E-20 | 5.43E-18 |
| Cenpe | 2.08134912 | | 3.89651981 | 84.6033503 | 3.72E-20 | 1.91E-17 |
| Prox1 | 1.82203753 | | 4.23489711 | 83.3780588 | 6.91E-20 | 3.47E-17 |
| Ccl21a | 1.2138727 | | 6.45170949 | 80.3881699 | 3.13E-19 | 1.54E-16 |
| Csf1 | -1.199371 | | 7.30292456 | 80.050293 | 3.72E-19 | 1.79E-16 |
| Icam1 | -1.1345478 | | 8.86348983 | 79.8774594 | 4.06E-19 | 1.91E-16 |
| Neurl3 | -1.2429446 | | 6.51799917 | 77.9389148 | 1.08E-18 | 4.98E-16 |
| Slc1a1 | -1.1798169 | | 7.16958724 | 76.5313436 | 2.20E-18 | 9.94E-16 |
| Ubash3b | -1.2208994 | | 6.59312189 | 75.544493 | 3.63E-18 | 1.60E-15 |
| Lgals3bp | 1.20849838 | | 6.17619862 | 74.614928 | 5.81E-18 | 2.52E-15 |
| Myh7 | -2.5464144 | | 3.57944933 | 73.4907289 | 1.03E-17 | 4.36E-15 |
| Plaur | -1.106522 | | 7.99676674 | 72.1290474 | 2.05E-17 | 8.53E-15 |
| Stip1 | -1.1664132 | | 6.71150058 | 71.2000065 | 3.28E-17 | 1.34E-14 |
| Fkbp5 | -1.4345276 | | 5.05417264 | 70.4151103 | 4.87E-17 | 1.96E-14 |
| Ahsa1 | -1.1835276 | | 6.37103902 | 69.9264918 | 6.24E-17 | 2.46E-14 |
| Cdc20 | 1.67569962 | | 4.2349623 | 68.8460647 | 1.08E-16 | 4.18E-14 |
| Tnfaip3 | -1.2376009 | | 5.86905239 | 68.7230521 | 1.15E-16 | 4.38E-14 |
| Angptl4 | 1.19637201 | | 5.75480095 | 67.9940263 | 1.66E-16 | 6.22E-14 |
| Dnaja1 | -1.0012272 | | 11.0387236 | 67.0431402 | 2.69E-16 | 9.91E-14 |
| Phf11d | 1.10621873 | | 6.64324838 | 66.8358186 | 2.99E-16 | 1.08E-13 |
| Gja1 | -1.050107 | | 8.31155064 | 66.6576145 | 3.27E-16 | 1.15E-13 |
| Ankrd1 | -1.1804869 | | 6.09298858 | 66.6530514 | 3.28E-16 | 1.15E-13 |
| Ms4a4d | 2.05761074 | | 3.44962185 | 66.4369174 | 3.66E-16 | 1.26E-13 |
| Tsc22d3 | -1.1462997 | | 6.42873681 | 66.1542653 | 4.22E-16 | 1.44E-13 |
| Birc3 | -1.1067363 | | 6.88923421 | 65.8877062 | 4.83E-16 | 1.62E-13 |
| Oasl2 | 1.10199651 | | 6.33208021 | 63.798643 | 1.39E-15 | 4.60E-13 |
| Gpm6a | 2.39881858 | | 2.91168689 | 62.8554616 | 2.25E-15 | 7.31E-13 |
| Apol9b | 2.63325382 | | 2.52499759 | 62.3982216 | 2.84E-15 | 9.09E-13 |
| Gzma | 2.34698127 | | 2.87164839 | 61.5279074 | 4.41E-15 | 1.39E-12 |
| Nod2 | -1.214943 | | 5.55734139 | 61.2264273 | 5.14E-15 | 1.58E-12 |
| Usp18 | 1.1232563 | | 5.76252384 | 60.6816997 | 6.78E-15 | 2.02E-12 |
| P4ha1 | -1.0180915 | | 7.62477437 | 60.1596546 | 8.84E-15 | 2.60E-12 |
| Ddx60 | 1.12990265 | | 5.64655473 | 59.9164078 | 1.00E-14 | 2.91E-12 |
| Hmmr | 2.14700063 | | 3.14969001 | 58.2936211 | 2.28E-14 | 6.38E-12 |
| G630055G22Rik | 2.25487208 | | 2.89982072 | 57.6291441 | 3.20E-14 | 8.83E-12 |
| Birc5 | 1.49305637 | | 4.30680777 | 57.4995806 | 3.41E-14 | 9.31E-12 |
| Cmpk2 | 1.04849311 | | 6.07384361 | 56.199602 | 6.61E-14 | 1.78E-11 |
| Gbp3 | 1.01001514 | | 6.59797398 | 55.8859198 | 7.75E-14 | 2.06E-11 |
| Plod2 | -1.0978257 | | 5.90150972 | 55.1361195 | 1.13E-13 | 2.95E-11 |
| Tgfb2 | 1.58362063 | | 3.98096096 | 55.0504065 | 1.19E-13 | 3.04E-11 |
| Pgf | -1.1538828 | | 5.54886841 | 54.4248598 | 1.63E-13 | 4.04E-11 |
| Acta1 | -1.2547824 | | 4.99794665 | 54.239777 | 1.79E-13 | 4.34E-11 |
| Map3k6 | -1.4046428 | | 4.61204283 | 53.8235924 | 2.21E-13 | 5.20E-11 |
| Dtx1 | 2.74614098 | | 2.0883368 | 53.4874439 | 2.62E-13 | 6.04E-11 |
| Ccl5 | 1.42143021 | | 4.19045532 | 53.5058503 | 2.60E-13 | 6.04E-11 |
| Aspm | 1.96466167 | | 3.29991864 | 53.134714 | 3.14E-13 | 7.15E-11 |
| Thy1 | 2.31006571 | | 2.64986906 | 52.6327847 | 4.05E-13 | 9.14E-11 |
| Zfp46 | -1.0482063 | | 6.03742841 | 52.0144467 | 5.55E-13 | 1.23E-10 |
| Gm4951 | 1.03403551 | | 5.79840874 | 51.8331366 | 6.09E-13 | 1.32E-10 |
| Cdkn3 | 2.37925811 | | 2.51759132 | 51.2814213 | 8.07E-13 | 1.73E-10 |
| Ccnb2 | 1.6140441 | | 3.88916601 | 50.8150355 | 1.02E-12 | 2.15E-10 |
| Reln | 1.61680207 | | 3.76326994 | 50.7147137 | 1.08E-12 | 2.23E-10 |
| Rab20 | -1.1182428 | | 5.43378636 | 49.9570101 | 1.58E-12 | 3.18E-10 |
| Pvr | -1.0326975 | | 5.95357996 | 49.3762967 | 2.13E-12 | 4.24E-10 |
| Agrn | 1.00047775 | | 5.87184667 | 49.2788778 | 2.24E-12 | 4.41E-10 |
| Nuak2 | -1.2003727 | | 4.96901028 | 49.1180584 | 2.43E-12 | 4.75E-10 |
| 4430402I18Rik | -1.1800517 | | 5.04780775 | 48.9037321 | 2.71E-12 | 5.25E-10 |
| Stx11 | -1.1493204 | | 5.16941741 | 48.6774227 | 3.04E-12 | 5.84E-10 |
| Hspa4l | -1.114008 | | 5.30131887 | 48.1902702 | 3.89E-12 | 7.42E-10 |
| Spp1 | 3.37650195 | | 1.26825046 | 47.773504 | 4.82E-12 | 8.94E-10 |
| Dhx58 | 1.79255913 | | 3.32510814 | 47.1376889 | 6.66E-12 | 1.21E-09 |
| Tead4 | -1.0334439 | | 5.66598506 | 46.2896022 | 1.03E-11 | 1.83E-09 |
| 9630014M24Rik | -1.3431207 | | 4.45203122 | 45.9796535 | 1.20E-11 | 2.09E-09 |
| Knl1 | 1.85589589 | | 3.20866225 | 45.7381654 | 1.36E-11 | 2.35E-09 |
| Rnf24 | -1.0704973 | | 5.33929665 | 45.2768706 | 1.72E-11 | 2.92E-09 |
| Ifi208 | 1.23248061 | | 4.41975731 | 45.0432071 | 1.94E-11 | 3.27E-09 |
| Tnfrsf10b | -1.0374497 | | 5.51429379 | 45.013105 | 1.97E-11 | 3.30E-09 |
| Cdk1 | 1.4144675 | | 4.05523481 | 44.6469858 | 2.37E-11 | 3.94E-09 |
| Prss23 | 1.43593916 | | 3.94792569 | 44.2608011 | 2.89E-11 | 4.73E-09 |
| Tpx2 | 1.39837496 | | 4.06814344 | 43.7729774 | 3.71E-11 | 5.94E-09 |
| Lockd | 1.53846088 | | 3.75341661 | 43.7906501 | 3.68E-11 | 5.94E-09 |
| Fosl1 | -1.1100462 | | 5.08469262 | 43.7823013 | 3.69E-11 | 5.94E-09 |
| Kif11 | 1.7158688 | | 3.30909489 | 42.2330769 | 8.14E-11 | 1.22E-08 |
| Lbp | 1.72458348 | | 3.26908218 | 42.1607235 | 8.45E-11 | 1.25E-08 |
| Lrg1 | 1.60431532 | | 3.51388212 | 42.133834 | 8.57E-11 | 1.26E-08 |
| Cenpa | 1.09222145 | | 4.77574963 | 40.9155362 | 1.60E-10 | 2.23E-08 |
| Ube2l6 | 1.01379381 | | 5.03959528 | 40.4199961 | 2.06E-10 | 2.82E-08 |
| Cdca8 | 1.49718035 | | 3.70601855 | 40.3024243 | 2.19E-10 | 2.98E-08 |
| Chsy3 | -1.0653161 | | 5.04114051 | 40.2390008 | 2.26E-10 | 3.06E-08 |
| Maf | 1.3022551 | | 4.07903662 | 40.0237126 | 2.52E-10 | 3.38E-08 |
| Phf11b | 1.83685899 | | 2.9601998 | 39.8894409 | 2.70E-10 | 3.59E-08 |
| Apol9a | 2.78308223 | | 1.53399922 | 39.7182511 | 2.95E-10 | 3.85E-08 |
| Lancl3 | -1.2580761 | | 4.38650884 | 39.1068479 | 4.03E-10 | 5.12E-08 |
| Pdk4 | 1.1978289 | | 4.26474069 | 38.6118624 | 5.19E-10 | 6.52E-08 |
| Pard6g | 1.44409573 | | 3.67886752 | 38.0810954 | 6.82E-10 | 8.37E-08 |
| Diaph3 | 1.50588684 | | 3.58829534 | 37.7644418 | 8.02E-10 | 9.52E-08 |
| Cdca3 | 2.08177732 | | 2.35015523 | 36.3763134 | 1.63E-09 | 1.88E-07 |
| Slc24a2 | 1.59850046 | | 3.22521062 | 35.7652859 | 2.23E-09 | 2.53E-07 |
| H1fx | 2.64003031 | | 1.47968099 | 35.2974652 | 2.84E-09 | 3.15E-07 |
| Parp11 | 1.09103697 | | 4.40093839 | 35.1325999 | 3.09E-09 | 3.38E-07 |
| Spsb1 | -1.0747494 | | 4.71124909 | 34.633623 | 3.99E-09 | 4.26E-07 |
| Anks1b | 1.41240594 | | 3.580764 | 34.4700184 | 4.34E-09 | 4.59E-07 |
| Hist1h1b | 1.04863322 | | 4.58303935 | 34.3119556 | 4.71E-09 | 4.93E-07 |
| Trim16 | -1.1135502 | | 4.55606197 | 34.2419935 | 4.88E-09 | 5.09E-07 |
| Gm14964 | 2.68957397 | | 1.33998059 | 34.1056901 | 5.24E-09 | 5.43E-07 |
| Anln | 1.66149787 | | 3.00686546 | 33.4065247 | 7.50E-09 | 7.60E-07 |
| Clca3a1 | 1.87813934 | | 2.51157365 | 33.2149139 | 8.28E-09 | 8.24E-07 |
| Sft2d3 | 1.15104414 | | 4.16358703 | 33.0829478 | 8.86E-09 | 8.76E-07 |
| Slc15a2 | -2.1805853 | | 3.10959602 | 43.9780456 | 1.10E-08 | 1.06E-06 |
| Ckap2 | 1.32061782 | | 3.77746941 | 32.5669355 | 1.16E-08 | 1.11E-06 |
| Tbx1 | 2.89984931 | | 1.03354828 | 31.4685009 | 2.03E-08 | 1.89E-06 |
| Mir99ahg | 1.04982366 | | 4.40288813 | 31.4354 | 2.07E-08 | 1.91E-06 |
| Per2 | -1.9927309 | | 2.85468963 | 31.4172938 | 2.09E-08 | 1.92E-06 |
| S100a4 | 1.25022805 | | 3.88948132 | 31.3947924 | 2.11E-08 | 1.94E-06 |
| Ptn | 1.55896722 | | 3.01931348 | 30.9745135 | 2.62E-08 | 2.35E-06 |
| Cenpw | 1.80178367 | | 2.5468985 | 30.4412531 | 3.45E-08 | 2.99E-06 |
| Ifi27l2b | 3.10801703 | | 0.85089906 | 30.3446691 | 3.63E-08 | 3.13E-06 |
| Sgms2 | -1.2025046 | | 4.18924777 | 30.1656983 | 3.98E-08 | 3.40E-06 |
| Lsmem1 | -1.0360743 | | 4.53908347 | 29.7669804 | 4.89E-08 | 4.09E-06 |
| Ikzf4 | -1.0931445 | | 4.37782672 | 29.7009072 | 5.05E-08 | 4.21E-06 |
| Plvap | 1.68697055 | | 2.68659057 | 29.2260763 | 6.46E-08 | 5.24E-06 |
| Cmah | 2.17637813 | | 1.77010105 | 29.0892589 | 6.93E-08 | 5.61E-06 |
| Lyve1 | 1.2537019 | | 3.61281145 | 28.5941003 | 8.95E-08 | 7.06E-06 |
| Tnfaip2 | -1.039101 | | 4.46616676 | 28.524214 | 9.28E-08 | 7.29E-06 |
| Kif20b | 1.46252299 | | 3.17016536 | 28.1522111 | 1.12E-07 | 8.68E-06 |
| Pclaf | 1.36508441 | | 3.28314686 | 26.6356677 | 2.46E-07 | 1.79E-05 |
| A730049H05Rik | -1.5065031 | | 3.31715304 | 25.8649618 | 3.67E-07 | 2.57E-05 |
| Ptpn3 | 1.73762827 | | 2.25457125 | 24.9631388 | 5.85E-07 | 3.91E-05 |
| Pimreg | 1.62396146 | | 2.55066892 | 24.7123768 | 6.67E-07 | 4.40E-05 |
| Gm5431 | 2.52747084 | | 1.02438836 | 24.6079035 | 7.04E-07 | 4.63E-05 |
| Gm15283 | -1.2060444 | | 3.78141931 | 24.3266576 | 8.15E-07 | 5.28E-05 |
| Ston2 | 1.6793295 | | 2.31781246 | 24.2925876 | 8.29E-07 | 5.34E-05 |
| Gzmb | 3.19057004 | | 0.42388381 | 24.010554 | 9.60E-07 | 6.08E-05 |
| Racgap1 | 1.29398684 | | 3.30916752 | 23.9600792 | 9.85E-07 | 6.20E-05 |
| Spc24 | 1.33916606 | | 3.18560658 | 23.9307184 | 1.00E-06 | 6.22E-05 |
| Melk | 2.07763261 | | 1.60889422 | 23.7431861 | 1.10E-06 | 6.80E-05 |
| C3 | -1.2653305 | | 3.59333834 | 23.6556698 | 1.15E-06 | 7.10E-05 |
| Hdx | -1.0009661 | | 4.25597028 | 23.5319833 | 1.23E-06 | 7.49E-05 |
| Kif15 | 1.81888496 | | 2.02032889 | 23.3730826 | 1.34E-06 | 8.05E-05 |
| Lcn2 | 1.28693868 | | 3.22948013 | 23.1746726 | 1.48E-06 | 8.87E-05 |
| Arhgap42 | 1.67395641 | | 2.27893185 | 23.1607976 | 1.49E-06 | 8.91E-05 |
| Nusap1 | 1.15044238 | | 3.64929962 | 23.1132483 | 1.53E-06 | 9.09E-05 |
| Csprs | 1.21814508 | | 3.38697277 | 22.7119138 | 1.88E-06 | 0.0001076 |
| Tnc | -1.2759291 | | 3.50353884 | 22.6782713 | 1.92E-06 | 0.00010922 |
| Klrb1f | 6.17082481 | | -0.6609263 | 22.4351291 | 2.18E-06 | 0.0001214 |
| Plce1 | 1.09466142 | | 3.68769611 | 21.8942821 | 2.89E-06 | 0.00015546 |
| Mis18bp1 | 1.44290608 | | 2.75582417 | 21.8872703 | 2.90E-06 | 0.00015565 |
| Cks1b | 1.06701262 | | 3.78715925 | 21.6998769 | 3.19E-06 | 0.00017037 |
| Ahnak2 | 1.69136492 | | 2.08151775 | 21.6901965 | 3.21E-06 | 0.00017082 |
| Retnlg | -2.2220438 | | 1.99332173 | 21.5438021 | 3.46E-06 | 0.00018217 |
| Wdr95 | -1.2700662 | | 3.45999311 | 21.4800763 | 3.58E-06 | 0.00018699 |
| Metrnl | -1.100842 | | 3.86220829 | 21.4380536 | 3.66E-06 | 0.00019068 |
| Sema3a | 1.42812043 | | 2.70078912 | 21.3776612 | 3.78E-06 | 0.00019629 |
| Xlr3b | 6.0311229 | | -0.669776 | 21.3600041 | 3.81E-06 | 0.00019721 |
| Oas1g | 1.58037996 | | 2.26237495 | 21.3509925 | 3.83E-06 | 0.00019767 |
| Kntc1 | 2.0072575 | | 1.46080972 | 21.1590464 | 4.23E-06 | 0.00021697 |
| Incenp | 1.24828691 | | 3.18705397 | 21.142509 | 4.27E-06 | 0.00021834 |
| Ckap2l | 1.41462311 | | 2.76210294 | 21.0946051 | 4.38E-06 | 0.00022283 |
| Lypd6 | 3.57889833 | | -0.0171167 | 20.8900274 | 4.87E-06 | 0.00024624 |
| Gm37245 | -2.2348606 | | 1.91324272 | 20.8800243 | 4.90E-06 | 0.00024673 |
| Cenpm | 1.63010835 | | 2.1521025 | 20.4635195 | 6.09E-06 | 0.00029811 |
| Tcf23 | 1.42823963 | | 2.58130387 | 20.430689 | 6.19E-06 | 0.00030193 |
| Thsd4 | 1.58805516 | | 2.14785631 | 19.8343972 | 8.46E-06 | 0.00039913 |
| Il1r2 | -2.8781554 | | 1.38426825 | 19.7823608 | 8.69E-06 | 0.00040715 |
| Nkg7 | 2.18698388 | | 1.08100882 | 19.5825369 | 9.65E-06 | 0.00044489 |
| Wdr17 | 1.55438897 | | 2.21493527 | 19.4269255 | 1.05E-05 | 0.00047963 |
| Ccnb1 | 1.51872907 | | 2.33659074 | 19.327594 | 1.10E-05 | 0.00050108 |
| Plk1 | 1.81578069 | | 1.67798983 | 19.2514744 | 1.15E-05 | 0.0005172 |
| Dapk1 | 1.25599345 | | 2.98185688 | 19.014464 | 1.30E-05 | 0.00057837 |
| Fut2 | -1.2449998 | | 3.34905528 | 18.5455399 | 1.66E-05 | 0.00071938 |
| Ubd | 1.67300213 | | 1.85714208 | 18.399749 | 1.79E-05 | 0.00076902 |
| Ptprc | 1.30989617 | | 2.73966995 | 18.3255259 | 1.86E-05 | 0.0007942 |
| Glis3 | 1.16316186 | | 3.15527074 | 18.133158 | 2.06E-05 | 0.00086767 |
| Eid2 | 1.32072464 | | 2.69036277 | 18.1008868 | 2.10E-05 | 0.00088082 |
| Fbxo5 | 1.42977817 | | 2.36378423 | 17.9814785 | 2.23E-05 | 0.00092551 |
| Ccl4 | 1.12324449 | | 3.34076266 | 17.8412736 | 2.40E-05 | 0.00098882 |
| 5830444B04Rik | 1.32034087 | | 2.67328061 | 17.801434 | 2.45E-05 | 0.00100413 |
| Tmod2 | 1.29348709 | | 2.7346903 | 17.7801291 | 2.48E-05 | 0.00101169 |
| Cenpk | 1.71490536 | | 1.71953741 | 17.6307422 | 2.68E-05 | 0.00108631 |
| Ccdc141 | 1.07290376 | | 3.40071584 | 17.4932577 | 2.89E-05 | 0.00115505 |
| Gm10638 | 1.26789393 | | 2.76942478 | 17.3797714 | 3.06E-05 | 0.00121291 |
| Mir155hg | -1.1353775 | | 3.4278792 | 17.3627803 | 3.09E-05 | 0.00122161 |
| Ptgs2 | -1.9866188 | | 1.93223498 | 17.3106488 | 3.18E-05 | 0.0012511 |
| Nuf2 | 1.57001081 | | 1.99766479 | 17.2519951 | 3.28E-05 | 0.00128118 |
| Ccna2 | 1.25406539 | | 2.84484325 | 16.8231561 | 4.11E-05 | 0.00156151 |
| Inhba | 2.38946022 | | 0.67210134 | 16.7340594 | 4.30E-05 | 0.00162212 |
| Tk1 | 1.33563909 | | 2.50536725 | 16.6700082 | 4.45E-05 | 0.00166976 |
| Kif22 | 1.21012144 | | 2.88311096 | 16.4485869 | 5.00E-05 | 0.00185137 |
| Nppb | -1.348034 | | 2.86647382 | 16.3906198 | 5.16E-05 | 0.00190248 |
| Efcab11 | 1.87515165 | | 1.28132451 | 16.3443205 | 5.29E-05 | 0.0019398 |
| Pbk | 1.3010954 | | 2.56920786 | 16.3458351 | 5.28E-05 | 0.0019398 |
| Lipg | -1.5469837 | | 2.41483304 | 16.1560133 | 5.84E-05 | 0.00210745 |
| Tchh | -1.1206098 | | 3.38179575 | 16.1509235 | 5.85E-05 | 0.00210968 |
| Ltb4r1 | -1.4555404 | | 2.60218277 | 16.0670942 | 6.12E-05 | 0.0021944 |
| Kif18b | 2.28810626 | | 0.7063744 | 16.002267 | 6.33E-05 | 0.00224967 |
| Kif2c | 2.02557718 | | 1.06185159 | 15.9504642 | 6.51E-05 | 0.00230388 |
| Gas7 | 1.23706044 | | 2.69525429 | 15.9149135 | 6.63E-05 | 0.0023438 |
| Wipf3 | 1.19103101 | | 2.78637549 | 15.6902744 | 7.47E-05 | 0.00260174 |
| Sphk1 | -1.2560375 | | 2.9968907 | 15.6421934 | 7.66E-05 | 0.00266033 |
| Csf3 | -1.598762 | | 2.28382121 | 15.4542786 | 8.46E-05 | 0.00288834 |
| Oas3 | 2.94003971 | | 0.0005601 | 15.3951694 | 8.73E-05 | 0.00297091 |
| Arhgap11a | 1.26810255 | | 2.52853653 | 15.3411943 | 8.98E-05 | 0.0030476 |
| Cfap69 | -1.3209886 | | 2.78840041 | 15.3380665 | 9.00E-05 | 0.00304796 |
| 2010315B03Rik | 1.1406216 | | 2.88098242 | 15.217576 | 9.59E-05 | 0.00322231 |
| Gjc2 | 2.92271903 | | -0.0013767 | 15.2074407 | 9.64E-05 | 0.00323148 |
| Gm17268 | -1.3704067 | | 2.68099428 | 15.1660189 | 9.85E-05 | 0.0032832 |
| Depdc1a | 1.61631418 | | 1.67301876 | 15.0343263 | 0.00010565 | 0.00349403 |
| Ncapg | 1.4365661 | | 2.06541826 | 14.9848988 | 0.00010845 | 0.00356539 |
| Cdca2 | 1.48607241 | | 1.95466479 | 14.9857608 | 0.0001084 | 0.00356539 |
| Cobl | -1.3173829 | | 2.76535931 | 14.8888458 | 0.00011412 | 0.00372386 |
| Tacc3 | 1.19471654 | | 2.73520517 | 14.8433979 | 0.0001169 | 0.00378669 |
| Phf11c | 1.35003693 | | 2.1892583 | 14.8446415 | 0.00011682 | 0.00378669 |
| Hap1 | 1.56933667 | | 1.66477516 | 14.5736672 | 0.00013488 | 0.00425064 |
| Col25a1 | -1.2497619 | | 2.89955173 | 14.5699359 | 0.00013514 | 0.004253 |
| Pdpn | 1.28479827 | | 2.32317484 | 14.537384 | 0.0001375 | 0.00431481 |
| Cfb | 1.11907898 | | 2.87878869 | 14.5016634 | 0.00014013 | 0.00438146 |
| Cdh11 | 1.84214887 | | 1.1525934 | 14.4182768 | 0.00014647 | 0.00455116 |
| Ndc80 | 1.04254885 | | 3.15268107 | 14.4153294 | 0.0001467 | 0.00455188 |
| Cacna1e | 3.19381601 | | -0.2155396 | 14.3799336 | 0.00014949 | 0.00462409 |
| Fam107a | -1.109358 | | 3.21050894 | 14.3777675 | 0.00014966 | 0.00462409 |
| Adamtsl1 | 1.11843598 | | 2.84540936 | 14.3626061 | 0.00015087 | 0.00464847 |
| Cip2a | 1.43713857 | | 2.01968862 | 14.3537889 | 0.00015157 | 0.00466378 |
| AC137513.1 | -1.3767748 | | 2.52312451 | 14.2946534 | 0.00015641 | 0.00479921 |
| Cd14 | -1.0463761 | | 3.40051295 | 14.1905884 | 0.0001653 | 0.00500939 |
| Chp2 | 1.92291061 | | 1.00414354 | 14.0294905 | 0.00018008 | 0.00539069 |
| Nppc | 2.62031239 | | 0.19756336 | 14.0023497 | 0.0001827 | 0.00544691 |
| Nek2 | 1.82963797 | | 1.1794285 | 13.982659 | 0.00018462 | 0.00548943 |
| Bub1b | 1.33304379 | | 2.15911004 | 13.900311 | 0.00019289 | 0.0057079 |
| Dlgap5 | 1.35975783 | | 2.11432753 | 13.8174986 | 0.00020158 | 0.0059297 |
| Cybb | 1.71834986 | | 1.27937365 | 13.760967 | 0.00020773 | 0.00609458 |
| E230013L22Rik | -1.3688321 | | 2.44462563 | 13.6280996 | 0.00022296 | 0.00647256 |
| Celsr1 | 2.28143707 | | 0.4687121 | 13.624888 | 0.00022334 | 0.00647513 |
| Mxd3 | 1.50145836 | | 1.72436412 | 13.5489573 | 0.00023256 | 0.0066897 |
| Gm16251 | -5.9483028 | | 0.17243682 | 13.506067 | 0.00023793 | 0.00681769 |
| Mns1 | 1.30297906 | | 2.17066263 | 13.4576559 | 0.00024415 | 0.00695974 |
| Gm16124 | 1.09985993 | | 2.76388369 | 13.3040507 | 0.00026499 | 0.00745404 |
| Zfp512b | 1.41963898 | | 1.84428039 | 13.2894479 | 0.00026706 | 0.00748709 |
| Ptx3 | -1.9126936 | | 1.60288783 | 13.2444037 | 0.00027355 | 0.00764972 |
| Cenph | 1.6937777 | | 1.25141066 | 13.2326282 | 0.00027527 | 0.00768819 |
| Gm13822 | 1.64442986 | | 1.30530684 | 13.1052094 | 0.00029464 | 0.00814667 |
| Plac8 | 1.6509323 | | 1.34042579 | 13.0713908 | 0.00030001 | 0.00824348 |
| Zfp157 | 1.07190906 | | 2.79020083 | 12.9542733 | 0.00031937 | 0.00873199 |
| Spc25 | 1.11534196 | | 2.74919799 | 12.8622752 | 0.00033545 | 0.00907079 |
| Esco2 | 1.16646682 | | 2.48825951 | 12.6997811 | 0.00036589 | 0.00979789 |
| Il7 | 1.31754923 | | 2.025435 | 12.6744416 | 0.00037088 | 0.00991953 |
| Sapcd2 | 2.08905568 | | 0.64443514 | 12.6682447 | 0.00037211 | 0.00994042 |
| 4930469K13Rik | 5.27005318 | | -1.0302305 | 12.6177672 | 0.0003823 | 0.01018829 |
| Cldn11 | 2.54489639 | | 0.09548737 | 12.5602269 | 0.00039425 | 0.0104684 |
| Ms4a6b | 1.63614081 | | 1.26727449 | 12.5456222 | 0.00039734 | 0.01053249 |
| Ralgps2 | 1.13779389 | | 2.52496904 | 12.4489703 | 0.00041843 | 0.01097869 |
| Cytip | 1.32746261 | | 1.99263379 | 12.3368353 | 0.00044433 | 0.01146742 |
| Lsr | 1.64490547 | | 1.26129582 | 12.2946487 | 0.00045448 | 0.01167495 |
| Pir | 1.0607088 | | 2.74783925 | 12.2567215 | 0.00046381 | 0.01184575 |
| Ncaph | 1.30855667 | | 2.00207173 | 12.2330799 | 0.00046973 | 0.01197777 |
| Gm17359 | -5.8007718 | | 0.07508644 | 12.2108794 | 0.00047535 | 0.01208447 |
| Gm20045 | -1.3083879 | | 2.4312908 | 12.2077792 | 0.00047614 | 0.01208658 |
| Iqgap3 | 1.23140325 | | 2.22212251 | 12.1758196 | 0.00048437 | 0.01227134 |
| 1700066B19Rik | -1.0425804 | | 3.14054959 | 12.1699292 | 0.0004859 | 0.01227918 |
| Sirpa | 1.26204041 | | 2.08607072 | 12.148821 | 0.00049143 | 0.01234891 |
| Ablim2 | 1.14193354 | | 2.46922969 | 12.1167703 | 0.00049994 | 0.01253664 |
| Gas2l3 | 1.36701171 | | 1.86896098 | 12.0529544 | 0.00051735 | 0.0128999 |
| Icosl | -1.3344608 | | 2.3713504 | 12.0437125 | 0.00051992 | 0.0129348 |
| Gdf10 | 3.71049363 | | -0.6778114 | 12.0446054 | 0.00051967 | 0.0129348 |
| Ldoc1 | 1.48400385 | | 1.50685416 | 12.0363288 | 0.00052198 | 0.01296183 |
| Gm15587 | -1.2351164 | | 2.56948897 | 11.9476541 | 0.00054742 | 0.01342241 |
| Isoc2b | 1.53771408 | | 1.38478861 | 11.9247795 | 0.00055418 | 0.01355811 |
| Ect2 | 1.03122059 | | 2.87915111 | 11.8336836 | 0.00058196 | 0.01411261 |
| Slc16a3 | -1.1665164 | | 2.73401215 | 11.7953382 | 0.00059406 | 0.01437467 |
| Gm42699 | -1.5114484 | | 1.99265588 | 11.7513834 | 0.00060826 | 0.01459025 |
| Sh3gl3 | 1.45914222 | | 1.55435741 | 11.7085886 | 0.0006224 | 0.01484988 |
| Zfp414 | 1.05899488 | | 2.62996724 | 11.6653448 | 0.00063704 | 0.01513272 |
| Neil3 | 1.25866716 | | 2.04111724 | 11.5759366 | 0.0006684 | 0.01560928 |
| Lepr | 1.82096396 | | 0.8522523 | 11.5531677 | 0.00067664 | 0.01576823 |
| Aqp9 | 2.8174856 | | -0.2332683 | 11.5485341 | 0.00067833 | 0.01579092 |
| Tmem51 | -1.1195565 | | 2.85906679 | 11.5442528 | 0.00067989 | 0.01581065 |
| Gm49980 | 1.1036827 | | 2.48441207 | 11.4964078 | 0.00069761 | 0.01610415 |
| Knstrn | 1.04431104 | | 2.74438505 | 11.4915551 | 0.00069944 | 0.01612939 |
| Gsta3 | -1.8720399 | | 1.453415 | 11.4190781 | 0.00072725 | 0.01666643 |
| Gm11772 | 1.54345085 | | 1.28650303 | 11.3278238 | 0.00076387 | 0.0173615 |
| Grb7 | 5.17164309 | | -1.1057811 | 11.3251792 | 0.00076496 | 0.01736837 |
| Gm15737 | -1.0545246 | | 2.95766987 | 11.2600112 | 0.00079228 | 0.01775159 |
| Gnat1 | -2.3833077 | | 0.94311811 | 11.2527401 | 0.00079539 | 0.01780319 |
| Capn11 | 2.0972658 | | 0.3911824 | 11.1030148 | 0.00086223 | 0.01908652 |
| Serpinb8 | 1.99337837 | | 0.57420385 | 11.0514033 | 0.00088656 | 0.01954682 |
| Nup37 | 1.01305052 | | 2.75636127 | 11.03351 | 0.00089516 | 0.01967745 |
| Ccl11 | -2.0408612 | | 1.20713275 | 10.9933838 | 0.00091475 | 0.01996893 |
| Scube1 | -1.2328686 | | 2.40843592 | 10.9662573 | 0.00092823 | 0.02022336 |
| Myot | -1.7154514 | | 1.59539283 | 10.9514679 | 0.00093567 | 0.02028537 |
| Hoxd9 | 1.73527564 | | 0.90314491 | 10.9177411 | 0.00095286 | 0.02059734 |
| Npl | 1.88170504 | | 0.7048791 | 10.9081456 | 0.0009578 | 0.02068405 |
| A830019P07Rik | 1.74619622 | | 0.87949328 | 10.8534786 | 0.00098649 | 0.02117369 |
| Gm29107 | -1.1374723 | | 2.61829322 | 10.7365075 | 0.00105083 | 0.02219398 |
| Slc7a6 | -1.0117405 | | 3.028395 | 10.6907841 | 0.00107712 | 0.0227058 |
| Klf15 | -1.6528852 | | 1.62983269 | 10.6610674 | 0.00109456 | 0.0230076 |
| Tusc1 | 1.27984367 | | 1.86744958 | 10.6573467 | 0.00109676 | 0.02303201 |
| Cxcl14 | -1.3340602 | | 2.13544497 | 10.6200232 | 0.00111912 | 0.02343467 |
| Slc45a3 | 1.47925953 | | 1.32276491 | 10.5832493 | 0.0011416 | 0.02376687 |
| Pglyrp1 | 1.25044942 | | 1.91051946 | 10.5607762 | 0.00115556 | 0.02399304 |
| Dnah2 | -1.0770935 | | 2.77113836 | 10.4208878 | 0.00124643 | 0.02552013 |
| Rasef | 2.02054985 | | 0.4394677 | 10.3309855 | 0.00130862 | 0.02661241 |
| Trub2 | 1.02138166 | | 2.5412917 | 10.294934 | 0.00133443 | 0.02701497 |
| Gm32250 | -1.3680088 | | 2.02730298 | 10.2709306 | 0.00135191 | 0.02727517 |
| Cep55 | 1.03073935 | | 2.59829547 | 10.2062778 | 0.00140013 | 0.02809406 |
| Gm28731 | -1.6696727 | | 1.54318812 | 10.1905949 | 0.00141208 | 0.02827277 |
| Ankrd6 | 1.76348698 | | 0.75298704 | 10.154997 | 0.00143961 | 0.02870378 |
| Fndc1 | 1.12366943 | | 2.12889134 | 10.063818 | 0.00151261 | 0.02986295 |
| Slc26a11 | -1.0135202 | | 2.88783366 | 10.0295315 | 0.00154103 | 0.03031553 |
| Dse | 1.55743067 | | 1.12853274 | 9.97010132 | 0.00159156 | 0.03114331 |
| Gm31763 | -1.0514543 | | 2.74269185 | 9.93167669 | 0.00162513 | 0.03165988 |
| Aurkb | 1.41656822 | | 1.35474956 | 9.86040344 | 0.0016893 | 0.0327081 |
| Ifit1bl2 | 1.25345712 | | 1.73985129 | 9.79410624 | 0.00175129 | 0.03378996 |
| Nrxn3 | 1.44812869 | | 1.27155619 | 9.79244855 | 0.00175287 | 0.03379092 |
| Gm38477 | -2.8286045 | | 0.4718288 | 9.76131906 | 0.0017828 | 0.03412606 |
| Cyp1a1 | -1.7773789 | | 1.31923823 | 9.7542498 | 0.00178966 | 0.03417415 |
| Greb1 | 4.96826882 | | -1.1903425 | 9.6315552 | 0.00191323 | 0.03621856 |
| Gm41496 | -1.1292502 | | 2.48049254 | 9.6056384 | 0.00194042 | 0.03654535 |
| Ccl6 | 1.18903219 | | 1.96063717 | 9.59694056 | 0.00194963 | 0.03662518 |
| Gm29282 | -1.2649767 | | 2.10832744 | 9.5034243 | 0.00205152 | 0.03823477 |
| Foxp4 | -1.0132047 | | 2.81445857 | 9.50244176 | 0.00205262 | 0.03823477 |
| Kif14 | 1.75973638 | | 0.6839628 | 9.47081575 | 0.0020883 | 0.03880135 |
| Entpd2 | -1.3321557 | | 2.01710456 | 9.44819225 | 0.00211422 | 0.03918396 |
| Sh2d5 | -1.8628059 | | 1.13403803 | 9.38211721 | 0.00219178 | 0.04014996 |
| Olfr373 | 4.88375566 | | -1.1948972 | 9.33861399 | 0.00224442 | 0.0409783 |
| Gm5820 | -1.6808698 | | 1.35812123 | 9.28368367 | 0.00231272 | 0.04194799 |
| Fgf14 | 1.80046457 | | 0.59206284 | 9.25953808 | 0.00234341 | 0.04240015 |
| Mastl | 1.11096036 | | 2.10648127 | 9.18970635 | 0.0024345 | 0.04349201 |
| Kifc1 | 1.48548671 | | 1.12577397 | 9.1540622 | 0.00248238 | 0.04411964 |
| Nptx1 | -2.4122318 | | 0.70517058 | 9.12470604 | 0.00252252 | 0.04469646 |
| BC065397 | -1.2223203 | | 2.15513699 | 9.08684496 | 0.00257528 | 0.04551271 |
| E2f8 | 1.64590924 | | 0.81835087 | 9.07295076 | 0.00259491 | 0.0457152 |
| 4930512B01Rik | -1.3624254 | | 1.85961247 | 9.06274706 | 0.00260943 | 0.04589778 |
| Espl1 | 1.56451606 | | 0.95033836 | 9.04855052 | 0.00262977 | 0.04603559 |
| Ska1 | 1.32396207 | | 1.49727983 | 9.03284454 | 0.00265246 | 0.04628604 |
| Cdhr3 | -1.8936549 | | 1.09957987 | 9.02386746 | 0.00266552 | 0.04647717 |
| H19 | 1.9662745 | | 0.30516159 | 8.99574839 | 0.00270684 | 0.04697522 |
| Bdh1 | -1.3457471 | | 1.88481646 | 8.99609069 | 0.00270633 | 0.04697522 |
| Guca2a | 4.78329822 | | -1.2006931 | 8.99386372 | 0.00270963 | 0.04698678 |
| Clec4d | -1.7786412 | | 1.23748565 | 8.9610985 | 0.00275865 | 0.04776188 |
| 1700016K05Rik | -1.0846958 | | 2.46117982 | 8.95000747 | 0.00277545 | 0.04801507 |
| 2010310C07Rik | -1.130246 | | 2.34735128 | 8.90241906 | 0.00284871 | 0.04906447 |
| Lrp1b | -1.0995455 | | 2.43442185 | 8.87416213 | 0.00289314 | 0.04962356 |
| Tox3 | 1.07369502 | | 2.08589412 | 8.86479699 | 0.00290801 | 0.04984006 |
| Aurka | 1.05146538 | | 2.25705751 | 8.85745103 | 0.00291974 | 0.04993195 |
| Stac | 1.92244341 | | 0.3776254 | 8.85664924 | 0.00292102 | 0.04993195 |
| **Fibroblasts** | | | | | | |
| **gene** | **logFC** | | **logCPM** | **F** | **pval** | **padj** |
| Ifit3 | 1.97083685 | | 6.26861046 | 86.7232864 | 1.48E-09 | 3.29E-05 |
| Plac8 | 2.71450421 | | 4.24346574 | 77.1015232 | 4.57E-09 | 3.39E-05 |
| Ifi27l2a | 1.47945733 | | 11.1091082 | 77.3136085 | 4.46E-09 | 3.39E-05 |
| Bdnf | -1.7307319 | | 5.78818854 | 70.3323886 | 1.08E-08 | 6.01E-05 |
| Ifit3b | 1.98432828 | | 4.52846769 | 66.4395349 | 1.82E-08 | 8.11E-05 |
| Ifi44 | 1.99193108 | | 4.28974792 | 63.8052868 | 2.63E-08 | 9.76E-05 |
| Cxcl5 | -2.3093967 | | 5.81613122 | 62.6129757 | 3.12E-08 | 9.92E-05 |
| Irf7 | 1.35544137 | | 6.31115028 | 58.8462131 | 5.43E-08 | 0.00015081 |
| Chil3 | 7.77952995 | | 1.25028522 | 60.636676 | 7.43E-08 | 0.0001652 |
| Tac1 | -2.0795157 | | 4.96679215 | 57.374043 | 6.78E-08 | 0.0001652 |
| Cxcl3 | -4.3487007 | | 3.12376588 | 55.11009 | 9.63E-08 | 0.00017481 |
| Cxcl2 | -2.3344903 | | 7.92719919 | 54.7346295 | 1.02E-07 | 0.00017481 |
| Ly6a | 1.0957044 | | 9.74682361 | 55.4950364 | 9.07E-08 | 0.00017481 |
| Cmpk2 | 1.49106264 | | 4.33388873 | 52.3424259 | 1.50E-07 | 0.00023831 |
| Rsad2 | 1.5703639 | | 6.0320936 | 51.7817752 | 1.64E-07 | 0.0002438 |
| Usp18 | 1.40759507 | | 4.57015007 | 50.0668009 | 2.19E-07 | 0.00030394 |
| Rab20 | -1.6265869 | | 5.86148973 | 46.3257334 | 4.17E-07 | 0.00051874 |
| Ddx60 | 1.34435144 | | 4.34149101 | 46.2885592 | 4.20E-07 | 0.00051874 |
| Sp110 | 1.12530139 | | 4.90299958 | 40.8275996 | 1.15E-06 | 0.00121947 |
| Hmgcs2 | 1.56087388 | | 4.85230133 | 39.1105565 | 1.61E-06 | 0.00162766 |
| Ifit2 | 1.42843847 | | 5.24053888 | 38.123857 | 1.96E-06 | 0.0018957 |
| Oasl2 | 1.04845119 | | 6.3756686 | 37.2008368 | 2.36E-06 | 0.00193571 |
| Ccl2 | -2.1804558 | | 9.77896756 | 37.714273 | 2.13E-06 | 0.00193571 |
| Pde10a | -1.4352407 | | 6.23917879 | 37.134286 | 2.40E-06 | 0.00193571 |
| Ms4a6c | 2.82046851 | | 2.25147967 | 37.3554686 | 2.29E-06 | 0.00193571 |
| Ifit1 | 1.56570503 | | 7.61046234 | 37.0518179 | 2.44E-06 | 0.00193571 |
| Nfkbib | -1.0122235 | | 5.25827196 | 36.5956042 | 2.68E-06 | 0.00198431 |
| Zbp1 | 1.35216282 | | 3.75712307 | 35.1991952 | 3.58E-06 | 0.00253488 |
| Apoc2 | 4.3533554 | | 1.31588974 | 35.1151918 | 3.65E-06 | 0.00253488 |
| Arhgap15 | 1.38663556 | | 4.46158634 | 34.7823956 | 3.92E-06 | 0.00262932 |
| Slc2a1 | -1.4194902 | | 5.27911968 | 34.657829 | 4.02E-06 | 0.00262932 |
| Nfkb1 | -1.5210365 | | 9.89634128 | 34.3029997 | 4.34E-06 | 0.00275575 |
| Hspd1 | -1.3927772 | | 7.97443969 | 33.6993419 | 4.94E-06 | 0.00296961 |
| Ahsa2 | -1.0770649 | | 5.57264511 | 33.7826309 | 4.85E-06 | 0.00296961 |
| Hspa5 | -1.1775719 | | 10.7497388 | 33.1143373 | 5.61E-06 | 0.00322623 |
| Mt2 | -1.7652237 | | 10.5442411 | 32.964823 | 5.80E-06 | 0.00322623 |
| Chordc1 | -1.0479508 | | 6.16699775 | 32.963122 | 5.80E-06 | 0.00322623 |
| Cybb | 2.36921285 | | 2.69844079 | 32.606966 | 6.28E-06 | 0.00340485 |
| Stip1 | -1.0855553 | | 6.37267438 | 32.4045648 | 6.57E-06 | 0.00347637 |
| Sertad4 | 1.404848 | | 3.58141262 | 31.5968243 | 7.87E-06 | 0.00388759 |
| Angptl4 | 1.3968589 | | 5.32184982 | 31.7751509 | 7.56E-06 | 0.00388759 |
| Tnfrsf12a | -1.2970478 | | 6.83262051 | 31.3522931 | 8.32E-06 | 0.00401918 |
| Thbs1 | -1.5749837 | | 9.86194021 | 30.8429613 | 9.34E-06 | 0.00415216 |
| Ubash3b | -1.3523623 | | 7.43314324 | 31.0731241 | 8.86E-06 | 0.00415216 |
| Ccl11 | -1.8941579 | | 7.52433529 | 30.9183233 | 9.18E-06 | 0.00415216 |
| Apol9b | 2.11964202 | | 2.12138424 | 30.7542405 | 9.53E-06 | 0.00415426 |
| Hspe1 | -1.2208367 | | 8.58013389 | 30.3449768 | 1.05E-05 | 0.00439203 |
| Mt1 | -1.1429314 | | 12.7201578 | 30.0329803 | 1.13E-05 | 0.00463366 |
| Cxcl1 | -1.2896871 | | 11.3996471 | 29.1382297 | 1.39E-05 | 0.00467531 |
| Hspb1 | -1.217453 | | 11.1266393 | 29.1681793 | 1.38E-05 | 0.00467531 |
| Ddit4 | -1.0147713 | | 4.91260323 | 29.6332608 | 1.24E-05 | 0.00467531 |
| Slfn2 | 1.14449259 | | 5.14683462 | 29.6941578 | 1.22E-05 | 0.00467531 |
| Sphk1 | -1.1507802 | | 5.2314565 | 29.3672733 | 1.31E-05 | 0.00467531 |
| Lox | -1.2482974 | | 5.19806359 | 29.4340091 | 1.29E-05 | 0.00467531 |
| Pqlc1 | -1.0398725 | | 6.30675254 | 28.9556571 | 1.45E-05 | 0.00480895 |
| Kctd11 | -1.3166222 | | 5.46698469 | 28.7886632 | 1.51E-05 | 0.00492997 |
| Ifi213 | 2.28965348 | | 2.21376556 | 28.7238178 | 1.53E-05 | 0.00493411 |
| Ighm | 1.59570689 | | 3.65777451 | 28.5346443 | 1.60E-05 | 0.00508827 |
| Oas2 | 1.42470776 | | 3.64580686 | 28.349806 | 1.67E-05 | 0.00524374 |
| Eps8 | -1.191426 | | 7.48554684 | 28.0423129 | 1.80E-05 | 0.00537312 |
| Nkg7 | 3.65849453 | | 1.79854938 | 28.0409471 | 1.80E-05 | 0.00537312 |
| Gm11973 | -2.0107275 | | 2.51827899 | 27.8438836 | 1.89E-05 | 0.00537312 |
| B830012L14Rik | -1.872228 | | 4.60329655 | 27.8696095 | 1.88E-05 | 0.00537312 |
| Plau | -1.2662971 | | 4.08382325 | 27.806371 | 1.91E-05 | 0.00537312 |
| Itgal | 2.15114662 | | 2.11261469 | 27.620985 | 2.00E-05 | 0.00548201 |
| Lpar4 | 1.4669411 | | 3.20498452 | 27.3879357 | 2.11E-05 | 0.00573216 |
| Foxs1 | -1.1257925 | | 4.28451215 | 27.3327698 | 2.14E-05 | 0.00573736 |
| Mcoln2 | -1.9261425 | | 5.29991878 | 27.2285865 | 2.20E-05 | 0.00573736 |
| Ccn3 | 1.04847069 | | 4.5775167 | 27.1899779 | 2.22E-05 | 0.00573736 |
| Arc | -1.727648 | | 5.00668198 | 27.2638001 | 2.18E-05 | 0.00573736 |
| Itga4 | 2.17167995 | | 2.06752896 | 27.1157247 | 2.26E-05 | 0.0057759 |
| Inhbb | 1.05221618 | | 4.66480075 | 27.025971 | 2.31E-05 | 0.00578747 |
| Coro1a | 1.57425515 | | 3.81841899 | 27.0153222 | 2.32E-05 | 0.00578747 |
| Ccbe1 | 1.00639006 | | 4.8121799 | 26.8444925 | 2.42E-05 | 0.00596981 |
| Dpp4 | 1.14728006 | | 4.6976456 | 26.7415989 | 2.48E-05 | 0.00599435 |
| Vipr2 | 1.53659156 | | 3.12560198 | 26.2922927 | 2.77E-05 | 0.00662817 |
| Adgrv1 | -1.2252429 | | 4.59775298 | 26.0613747 | 2.94E-05 | 0.00687559 |
| Gm34907 | -1.0866597 | | 4.7983856 | 25.5115763 | 3.38E-05 | 0.0077384 |
| Hspa4l | -1.2581285 | | 5.04892928 | 25.300598 | 3.56E-05 | 0.00807715 |
| Il6 | -1.3587708 | | 8.99930733 | 25.2193937 | 3.64E-05 | 0.00807715 |
| Satb1 | 1.11217132 | | 4.36304978 | 25.2090016 | 3.65E-05 | 0.00807715 |
| Ccl5 | 2.63985462 | | 3.75174563 | 25.0507183 | 3.80E-05 | 0.00827916 |
| March3 | -1.0619161 | | 8.13649579 | 24.7451051 | 4.11E-05 | 0.00870171 |
| Rin2 | 1.02430557 | | 5.21557775 | 24.4610039 | 4.42E-05 | 0.00919204 |
| Il33 | -1.2479756 | | 6.28036624 | 24.243245 | 4.68E-05 | 0.00955062 |
| Sod2 | -1.1127446 | | 7.56154479 | 23.9635006 | 5.04E-05 | 0.01000219 |
| Ear2 | 6.41904976 | | 0.36037382 | 24.6148758 | 5.32E-05 | 0.01012041 |
| Gm16685 | -1.9791386 | | 5.76582429 | 23.660635 | 5.46E-05 | 0.01022665 |
| Ifitm1 | -1.0498551 | | 5.42826304 | 23.6496928 | 5.47E-05 | 0.01022665 |
| Gm17268 | -1.3424166 | | 5.53556378 | 23.5049482 | 5.69E-05 | 0.01047551 |
| Mpeg1 | 2.09278587 | | 1.83091585 | 23.4962294 | 5.70E-05 | 0.01047551 |
| Il1r1 | -1.1723042 | | 8.56549141 | 23.2424584 | 6.10E-05 | 0.01055895 |
| Cytip | 1.61489296 | | 3.60081168 | 23.3010449 | 6.01E-05 | 0.01055895 |
| Retnlg | -4.0410976 | | 4.5724042 | 23.226255 | 6.13E-05 | 0.01055895 |
| Ptx3 | -1.8123045 | | 8.08469957 | 23.0733901 | 6.38E-05 | 0.01091553 |
| Fosl1 | -1.0285124 | | 4.94228112 | 22.9502523 | 6.60E-05 | 0.01115127 |
| Ctps | -1.1274464 | | 5.12679552 | 22.6666354 | 7.12E-05 | 0.01147392 |
| Egr3 | -1.3841415 | | 6.26384649 | 22.7187266 | 7.02E-05 | 0.01147392 |
| Lipg | -2.1963459 | | 2.95794037 | 22.72939 | 7.00E-05 | 0.01147392 |
| Pdk4 | 1.27051113 | | 3.17897541 | 22.5138035 | 7.42E-05 | 0.01165374 |
| Zfp516 | -1.1904737 | | 5.20119923 | 22.5039454 | 7.44E-05 | 0.01165374 |
| Tfpi2 | -1.6070071 | | 5.81169121 | 22.2031021 | 8.08E-05 | 0.01221966 |
| Ly6c2 | 2.52647185 | | 2.20480819 | 22.1067137 | 8.30E-05 | 0.01229562 |
| Dhx58 | 1.41545031 | | 2.75540527 | 21.6720164 | 9.35E-05 | 0.01361598 |
| Ttyh2 | -1.6321386 | | 3.96046615 | 21.1468033 | 0.00010825 | 0.01531299 |
| Zc3h6 | 1.13505085 | | 3.8799202 | 20.4928438 | 0.00013022 | 0.01723336 |
| Piga | -1.2440275 | | 3.29543789 | 20.559403 | 0.00012778 | 0.01723336 |
| Csprs | 1.55820307 | | 2.27681093 | 20.5227269 | 0.00012912 | 0.01723336 |
| Napsa | 1.99529119 | | 2.18375035 | 20.4081303 | 0.0001334 | 0.0172696 |
| Cemip2 | -1.1024059 | | 6.20006145 | 20.4271728 | 0.00013268 | 0.0172696 |
| Hsph1 | -1.2058807 | | 8.05393207 | 20.3616696 | 0.00013519 | 0.01730079 |
| A230001M10Rik | 1.05342381 | | 3.67823524 | 20.1490821 | 0.00014367 | 0.01794527 |
| Ptgs2os | -1.5597981 | | 4.48768896 | 20.0474693 | 0.00014793 | 0.01827194 |
| Gch1 | -1.0507468 | | 6.77507094 | 20.0668084 | 0.00014711 | 0.01827194 |
| Col20a1 | -1.168569 | | 3.69345218 | 20.0193058 | 0.00014914 | 0.01831894 |
| 9930111J21Rik2 | 1.1169161 | | 5.14747951 | 19.7745717 | 0.00016006 | 0.01896617 |
| Hspe1-rs1 | -1.6196095 | | 2.63906166 | 19.847525 | 0.00015672 | 0.01896617 |
| Nab2 | -1.0864901 | | 3.68513077 | 19.4797206 | 0.00017439 | 0.02000449 |
| Chsy3 | -1.2091215 | | 6.62315871 | 19.4790836 | 0.00017442 | 0.02000449 |
| Ptgs2 | -1.3487148 | | 8.25978206 | 19.3940004 | 0.00017882 | 0.02038651 |
| Ak4 | -1.3382595 | | 2.99132026 | 19.3593282 | 0.00018064 | 0.02038651 |
| 2700038G22Rik | -1.5023943 | | 3.17900614 | 19.2963861 | 0.000184 | 0.02055722 |
| Ankrd33b | -1.108707 | | 4.55742687 | 19.1856185 | 0.00019008 | 0.02092625 |
| Peg10 | -1.1089775 | | 4.25445227 | 18.9429541 | 0.00020418 | 0.02182513 |
| Ikzf1 | 1.92190055 | | 1.69736948 | 18.8807007 | 0.00020798 | 0.02201934 |
| Ccl19 | -1.4659246 | | 5.16041728 | 18.8088199 | 0.00021246 | 0.02208812 |
| Hs3st3b1 | -1.2003553 | | 4.12165639 | 18.8362843 | 0.00021074 | 0.02208812 |
| Cstdc4 | -4.4059919 | | 0.93485724 | 18.835165 | 0.00021081 | 0.02208812 |
| Eid2 | 1.3588634 | | 2.92729662 | 18.5687285 | 0.0002282 | 0.02316735 |
| Dct | 4.25896182 | | 1.23329072 | 18.4810106 | 0.00023427 | 0.02367475 |
| Ccl7 | -1.1730104 | | 9.66055066 | 18.4634518 | 0.0002355 | 0.02369182 |
| Rcl1 | -1.0360469 | | 4.82840442 | 18.343272 | 0.00024414 | 0.02440323 |
| Grap2 | 2.02607095 | | 1.8585861 | 18.2255413 | 0.00025294 | 0.02499402 |
| Adamts6 | 1.01025123 | | 3.77232006 | 18.1669939 | 0.00025745 | 0.02521796 |
| Laptm5 | 1.38222689 | | 3.50026174 | 17.8590818 | 0.00028261 | 0.0272039 |
| Oas1a | 1.12045424 | | 3.33195307 | 17.7941482 | 0.00028825 | 0.0272039 |
| Serpinb6b | 1.81624983 | | 1.73240135 | 17.8267541 | 0.0002854 | 0.0272039 |
| Ms4a4c | 2.29431085 | | 1.74118227 | 17.7882685 | 0.00028877 | 0.0272039 |
| Procr | -1.1613048 | | 4.15918458 | 17.7198343 | 0.00029485 | 0.02742881 |
| Slc9a7 | 2.91207825 | | 0.769049 | 17.5790756 | 0.00030782 | 0.0283973 |
| Lvrn | -1.0137178 | | 5.46863412 | 17.5469492 | 0.00031086 | 0.02855972 |
| Pou2f2 | 1.58963761 | | 2.50476251 | 17.4881732 | 0.00031652 | 0.02872328 |
| Abat | -1.1102272 | | 3.32196544 | 17.438041 | 0.00032143 | 0.02898705 |
| Rnf125 | -1.014585 | | 5.11299266 | 17.2733996 | 0.00033816 | 0.02995326 |
| Tmem95 | -1.3228297 | | 3.19728645 | 17.1459832 | 0.00035175 | 0.03103353 |
| Carmn | -1.093384 | | 3.22035646 | 17.0215128 | 0.0003656 | 0.03175161 |
| Ucp2 | 1.15845989 | | 4.13939358 | 16.7873048 | 0.0003933 | 0.03288031 |
| Cd300a | 1.70836206 | | 1.97488996 | 16.8532221 | 0.00038528 | 0.03288031 |
| Pld4 | 2.41729121 | | 1.15356226 | 16.7388907 | 0.0003993 | 0.03288031 |
| Mrap | -1.2172115 | | 2.98006404 | 16.769086 | 0.00039555 | 0.03288031 |
| Rnd1 | -1.0218695 | | 5.12754541 | 16.5216893 | 0.00042749 | 0.03443641 |
| Tnc | -1.7436031 | | 5.22742438 | 16.2043364 | 0.00047264 | 0.03623526 |
| March3 | 2.31724129 | | 1.04874791 | 16.244914 | 0.00046659 | 0.03623526 |
| Batf | -1.0023071 | | 4.07567104 | 16.2088125 | 0.00047197 | 0.03623526 |
| Adgre4 | 3.73151836 | | 0.78618989 | 16.2059571 | 0.0004724 | 0.03623526 |
| Il4 | 4.19714879 | | 0.08332758 | 16.1376609 | 0.00048277 | 0.03663284 |
| Ms4a1 | 2.13871767 | | 1.82629059 | 16.1033655 | 0.00048807 | 0.03665976 |
| Jak2 | -1.0744137 | | 7.22829636 | 16.1076395 | 0.00048741 | 0.03665976 |
| Traf1 | 1.80591658 | | 1.99505063 | 16.0698738 | 0.00049331 | 0.03692846 |
| Otud7a | -1.4121199 | | 2.86930748 | 15.9837766 | 0.00050706 | 0.03757809 |
| Rffl | -1.056323 | | 4.95300551 | 15.9281354 | 0.00051616 | 0.0378742 |
| Cd101 | -1.2937525 | | 2.73876919 | 15.8854712 | 0.00052327 | 0.0379381 |
| Ighd | 1.6980145 | | 2.01264253 | 15.8985702 | 0.00052108 | 0.0379381 |
| Gm21188 | 4.04192122 | | -0.1306887 | 15.7767196 | 0.00054186 | 0.03848913 |
| Ifi206 | 2.31719778 | | 1.39089119 | 15.7446863 | 0.00054747 | 0.03864082 |
| Tgif2 | -1.384807 | | 2.44497724 | 15.746438 | 0.00054716 | 0.03864082 |
| G630055G22Rik | 2.54491027 | | 0.98596499 | 15.6921821 | 0.0005568 | 0.0390255 |
| Lyz2 | 1.47539165 | | 6.15669538 | 15.6406092 | 0.00056614 | 0.03933435 |
| Ifi214 | 5.72698427 | | -0.4054229 | 15.9938959 | 0.00057518 | 0.0397141 |
| Nkain3 | 1.14200795 | | 3.1091937 | 15.4264146 | 0.00060678 | 0.04075703 |
| Stat5a | -1.1067588 | | 4.31134965 | 15.4318317 | 0.00060572 | 0.04075703 |
| Gm44717 | -5.6235337 | | -0.1986046 | 15.6493821 | 0.00064008 | 0.04222811 |
| H2-T24 | 1.25511674 | | 2.51988852 | 15.1900518 | 0.00065534 | 0.04260293 |
| Iqgap2 | -1.3368899 | | 4.15439976 | 15.116299 | 0.00067135 | 0.04313243 |
| Ch25h | -1.536826 | | 5.4872516 | 15.0991542 | 0.00067513 | 0.04313243 |
| Ifitm6 | 2.16749633 | | 1.42313186 | 14.992725 | 0.00069912 | 0.04408102 |
| Stat4 | 1.53844645 | | 3.13250809 | 14.897666 | 0.00072134 | 0.04504843 |
| Ccr2 | 2.38624767 | | 0.66813717 | 14.8172639 | 0.00074073 | 0.04587343 |
| Hpgd | 1.44279294 | | 3.50559679 | 14.7765795 | 0.00075075 | 0.0462368 |
| Spn | 3.34015814 | | 0.3766536 | 14.6897206 | 0.00077266 | 0.04686525 |
| Gm31522 | -1.875924 | | 2.06206712 | 14.690356 | 0.00077249 | 0.04686525 |
| Myo1g | 2.37950022 | | 1.18332453 | 14.6093081 | 0.00079355 | 0.04692309 |
| Il10ra | 2.18318675 | | 1.10264338 | 14.5676124 | 0.00080463 | 0.04732635 |
| Ly6k | -1.6320142 | | 3.10640967 | 14.5268771 | 0.00081562 | 0.0474701 |
| Per2 | -1.2140801 | | 3.17129027 | 14.4811871 | 0.00082813 | 0.04794588 |
| Rnase6 | 3.03176732 | | 0.59777288 | 14.4658633 | 0.00083237 | 0.04794588 |
| S100a8 | -2.2202975 | | 6.54378895 | 14.4370966 | 0.00084041 | 0.04818933 |
| Gimap7 | 5.42209392 | | -0.5300526 | 14.7490286 | 0.00085077 | 0.04818933 |
| Tnip3 | 2.8223202 | | 0.66816465 | 14.3515414 | 0.00086479 | 0.04818933 |
| H1fx | 1.21036688 | | 3.15732658 | 14.3698268 | 0.00085952 | 0.04818933 |
| E2f7 | -1.2706598 | | 2.73356016 | 14.4039831 | 0.00084975 | 0.04818933 |
| Dock2 | 1.2223177 | | 3.86212908 | 14.3938947 | 0.00085262 | 0.04818933 |
| Nckap1l | 1.97864748 | | 1.16576984 | 14.386977 | 0.0008546 | 0.04818933 |
| Phf11b | 1.26039237 | | 2.55860138 | 14.296087 | 0.00088101 | 0.04896869 |
| Fcmr | 1.77734971 | | 1.88534796 | 14.2795791 | 0.0008859 | 0.04911776 |
| Ctss | 1.59113682 | | 3.05766325 | 14.2454526 | 0.00089611 | 0.04955999 |
| **Granulocytes** | | | | | | |
| **gene** | **logFC** | | **logCPM** | **F** | **pval** | **padj** |
| Ccl21a | 6.60417642 | | 7.09691195 | 90.8475096 | 1.61E-21 | 2.32E-17 |
| Retnlg | -2.2407406 | | 10.8192584 | 78.7273696 | 7.33E-19 | 5.29E-15 |
| Areg | 5.7277875 | | 6.8358707 | 71.9449593 | 2.26E-17 | 8.17E-14 |
| Ifi27l2a | 2.28494848 | | 9.94333887 | 72.0835858 | 2.11E-17 | 8.17E-14 |
| S100a8 | -1.3901268 | | 13.669442 | 62.9384054 | 2.17E-15 | 6.26E-12 |
| S100a9 | -1.2335064 | | 14.1626796 | 55.1188837 | 1.15E-13 | 2.77E-10 |
| Ngp | -7.7608555 | | 7.34422033 | 52.7860104 | 3.77E-13 | 7.76E-10 |
| Cd74 | -1.8443533 | | 10.2240344 | 48.0881611 | 4.12E-12 | 7.42E-09 |
| Camp | -5.2839563 | | 7.40090963 | 46.8428383 | 7.77E-12 | 1.25E-08 |
| Gzmb | -4.5846641 | | 7.49811583 | 45.5233985 | 1.52E-11 | 2.20E-08 |
| Ccl2 | 2.27997807 | | 8.32772492 | 41.4310419 | 1.23E-10 | 1.61E-07 |
| Igfbp5 | 3.09161342 | | 7.06340854 | 39.0890774 | 4.08E-10 | 4.90E-07 |
| Thbs1 | -2.0554646 | | 9.08881462 | 38.1649959 | 6.54E-10 | 6.74E-07 |
| Gk | -3.6615101 | | 7.52905145 | 38.2633977 | 6.22E-10 | 6.74E-07 |
| Ptprj | -2.2885676 | | 8.67991996 | 37.5977438 | 8.75E-10 | 8.42E-07 |
| H2-Eb1 | -2.2747761 | | 8.64127588 | 36.6523655 | 1.42E-09 | 1.21E-06 |
| Fth1 | -1.1880033 | | 12.5061203 | 36.7061278 | 1.38E-09 | 1.21E-06 |
| Csf2rb | -2.911697 | | 7.92200095 | 36.370023 | 1.64E-09 | 1.32E-06 |
| Xylt1 | -1.6150058 | | 10.0348304 | 35.7492978 | 2.26E-09 | 1.71E-06 |
| Stx11 | -2.7074753 | | 7.87039722 | 32.4757058 | 1.21E-08 | 8.75E-06 |
| Irf7 | 2.42548591 | | 7.41349263 | 31.6208918 | 1.88E-08 | 1.29E-05 |
| Alcam | -1.5653628 | | 9.78998988 | 30.7656383 | 2.92E-08 | 1.92E-05 |
| Actg1 | -1.1832794 | | 11.6877581 | 30.4964854 | 3.36E-08 | 2.11E-05 |
| Antxr2 | -1.427232 | | 10.2032429 | 29.8070134 | 4.79E-08 | 2.88E-05 |
| Il1r2 | -1.4254582 | | 10.1509379 | 29.1341783 | 6.78E-08 | 3.91E-05 |
| Nfkb1 | -1.7784736 | | 9.02510893 | 28.8397859 | 7.89E-08 | 4.38E-05 |
| Dhrs9 | -2.9138304 | | 7.4677015 | 27.6411936 | 1.47E-07 | 7.83E-05 |
| Ifit2 | 2.63185416 | | 6.7599924 | 27.0696194 | 1.97E-07 | 0.00010146 |
| Rbm47 | -3.2340673 | | 7.20523754 | 26.9346224 | 2.11E-07 | 0.00010504 |
| Clec4e | -1.747078 | | 8.84665937 | 26.2385068 | 3.03E-07 | 0.00014557 |
| Plek | -1.3291741 | | 10.0695066 | 24.9659141 | 5.85E-07 | 0.00027234 |
| Retnla | 4.03459638 | | 5.78726242 | 24.8028293 | 6.37E-07 | 0.00028711 |
| Slpi | -1.379586 | | 9.85786378 | 24.7377993 | 6.59E-07 | 0.00028796 |
| Wfdc21 | -2.335957 | | 7.80968709 | 24.4951065 | 7.47E-07 | 0.00031698 |
| Tyrobp | -1.1235612 | | 11.0927735 | 23.8127544 | 1.06E-06 | 0.00043349 |
| Gzmc | -6.5461458 | | 6.33078824 | 23.7819348 | 1.08E-06 | 0.00043349 |
| Btbd9 | -1.5082385 | | 9.196559 | 23.7177962 | 1.12E-06 | 0.00043606 |
| Cxcl1 | -2.3009913 | | 7.71336947 | 23.4478917 | 1.29E-06 | 0.00048849 |
| H2-Ab1 | -1.8129303 | | 8.35968879 | 22.7354919 | 1.86E-06 | 0.00068932 |
| Fam71f2 | 3.80068645 | | 5.74554884 | 22.3834085 | 2.24E-06 | 0.00080722 |
| Ltf | -6.405848 | | 6.25983538 | 22.1693079 | 2.50E-06 | 0.00088038 |
| Stfa2l1 | -2.2961776 | | 7.64575846 | 21.8386502 | 2.97E-06 | 0.0010126 |
| Vsig4 | 4.76039268 | | 5.42110315 | 21.8092159 | 3.02E-06 | 0.0010126 |
| Ifrd1 | -1.2994904 | | 9.76423458 | 21.7235463 | 3.16E-06 | 0.00103475 |
| Hp | -1.5936281 | | 8.85726898 | 21.5285006 | 3.49E-06 | 0.00112001 |
| Mmp8 | -2.4294416 | | 7.46272844 | 21.4222787 | 3.69E-06 | 0.00115805 |
| Cd14 | -1.4486557 | | 9.15428048 | 21.3603462 | 3.81E-06 | 0.0011706 |
| Igf1r | -1.4100674 | | 9.16239502 | 20.6949058 | 5.40E-06 | 0.00162198 |
| Rgs1 | -1.6742191 | | 8.48216309 | 20.3799678 | 6.36E-06 | 0.00185624 |
| Diaph1 | -1.6451745 | | 8.4908155 | 20.3584178 | 6.43E-06 | 0.00185624 |
| Gda | -1.5254664 | | 8.80083387 | 19.8734261 | 8.29E-06 | 0.00234488 |
| H2-Aa | -1.4898952 | | 8.85696889 | 19.7947503 | 8.64E-06 | 0.00239638 |
| Mt2 | 1.91622126 | | 7.27958798 | 19.3315789 | 1.10E-05 | 0.00299391 |
| Cstdc4 | -2.5518601 | | 7.16013248 | 19.2971986 | 1.12E-05 | 0.00299391 |
| Cbr2 | 5.75214346 | | 5.09060277 | 19.2243925 | 1.16E-05 | 0.0030537 |
| Isg15 | 1.22185664 | | 9.47772622 | 18.9871761 | 1.32E-05 | 0.00339596 |
| Sgms2 | -1.1680927 | | 9.9601962 | 18.8971266 | 1.38E-05 | 0.00349757 |
| Pfkfb3 | -2.5319996 | | 7.11126539 | 18.8439918 | 1.42E-05 | 0.00353433 |
| Cd300lf | -2.1834195 | | 7.4232828 | 18.7722713 | 1.48E-05 | 0.00360752 |
| Arl4c | -4.0287467 | | 6.37618918 | 18.3644928 | 1.83E-05 | 0.00432121 |
| Vma21 | 3.03504306 | | 5.92228485 | 18.2148728 | 1.98E-05 | 0.00459878 |
| Itgam | -1.7746519 | | 7.95626091 | 17.4826373 | 2.90E-05 | 0.00644445 |
| Sqstm1 | -1.3495103 | | 8.95436426 | 17.4977672 | 2.88E-05 | 0.00644445 |
| Pdzrn3 | 4.37496204 | | 5.29879012 | 17.3743617 | 3.07E-05 | 0.00657998 |
| Adam8 | -1.8032831 | | 7.90255702 | 17.4130267 | 3.01E-05 | 0.00657998 |
| Tnfaip3 | -2.0466212 | | 7.5234593 | 17.3572713 | 3.10E-05 | 0.00657998 |
| Clec4d | -1.1363054 | | 9.80735525 | 16.8708033 | 4.01E-05 | 0.008377 |
| Lcn2 | -1.6344448 | | 8.09170948 | 16.7966235 | 4.17E-05 | 0.00858637 |
| Grina | -1.3707942 | | 8.83232847 | 16.7027751 | 4.38E-05 | 0.00889456 |
| Prss34 | 4.2905216 | | 5.27593225 | 16.6060185 | 4.61E-05 | 0.00913594 |
| Samsn1 | -1.2726008 | | 9.05546761 | 16.2740776 | 5.49E-05 | 0.01069847 |
| Chst11 | -1.2619114 | | 9.07134873 | 16.0616736 | 6.14E-05 | 0.01174936 |
| Dcn | -2.0522289 | | 7.30529648 | 16.0460237 | 6.19E-05 | 0.01174936 |
| Cd44 | -1.0253909 | | 10.1749143 | 15.7545937 | 7.22E-05 | 0.01342541 |
| Fnip2 | -1.7430824 | | 7.78075362 | 15.7443978 | 7.26E-05 | 0.01342541 |
| Sirpa | -2.0844127 | | 7.26717287 | 15.6443016 | 7.65E-05 | 0.01397566 |
| Gm17268 | 3.2508833 | | 5.63439761 | 15.609069 | 7.80E-05 | 0.01406043 |
| Pram1 | -5.9021847 | | 5.89126972 | 15.4618654 | 8.43E-05 | 0.01464935 |
| Rsad2 | 1.23307369 | | 8.73403453 | 15.2669486 | 9.34E-05 | 0.01604752 |
| Slc16a3 | -1.3799516 | | 8.57861264 | 15.1739817 | 9.82E-05 | 0.01665873 |
| Fxyd5 | -1.0339479 | | 9.99769064 | 15.0888363 | 0.00010268 | 0.01703732 |
| Bst2 | 1.19998784 | | 8.85424888 | 15.0876248 | 0.00010275 | 0.01703732 |
| Aqp1 | -1.0999345 | | 9.58330901 | 15.0453816 | 0.00010507 | 0.01722483 |
| Zeb2 | -1.0536261 | | 9.81500169 | 14.9136762 | 0.00011267 | 0.01826216 |
| Mgp | -2.1118929 | | 7.12186705 | 14.8274016 | 0.00011794 | 0.0189042 |
| Lgals3 | -1.5041878 | | 8.21400346 | 14.7633028 | 0.00012201 | 0.01934272 |
| Rlf | -2.2855169 | | 6.99030178 | 14.7106312 | 0.00012547 | 0.01967437 |
| Msrb1 | -1.0644562 | | 9.70172664 | 14.6311544 | 0.00013087 | 0.02030077 |
| Lmnb1 | -1.1825544 | | 9.12255594 | 14.495028 | 0.00014068 | 0.02158915 |
| Apbb1ip | -1.4836918 | | 8.14815709 | 14.4026345 | 0.00014775 | 0.02213493 |
| Ckm | -2.3874424 | | 6.82925563 | 14.3888124 | 0.00014883 | 0.02213493 |
| Dock10 | -1.6680365 | | 7.73987207 | 14.3544293 | 0.00015158 | 0.02231271 |
| Cyth4 | -2.1493927 | | 7.11431801 | 14.3114099 | 0.00015508 | 0.02246354 |
| Pi16 | -3.7352003 | | 6.19124852 | 14.3036999 | 0.00015572 | 0.02246354 |
| Serpinb2 | -5.7974662 | | 5.79825605 | 14.1410913 | 0.00016977 | 0.02392117 |
| Wfdc17 | -1.4893215 | | 8.11987022 | 14.1297221 | 0.00017079 | 0.02392117 |
| Il15 | -1.4438081 | | 8.18152963 | 14.0801514 | 0.00017535 | 0.02409199 |
| Ifitm1 | -1.0444735 | | 9.77644686 | 14.0101242 | 0.00018201 | 0.02476988 |
| Il6 | -1.9185229 | | 7.39826329 | 13.9419992 | 0.00018872 | 0.02544362 |
| Sorl1 | -1.1439572 | | 9.17717697 | 13.9193989 | 0.000191 | 0.02551286 |
| Csf2rb2 | -3.7237195 | | 6.16685858 | 13.8996775 | 0.00019302 | 0.02554536 |
| Sdc4 | -3.7041305 | | 6.14346567 | 13.8029139 | 0.00020321 | 0.02599765 |
| Kcnd2 | 5.17984435 | | 4.87303227 | 13.765982 | 0.00020725 | 0.02599765 |
| Mcemp1 | -1.6313641 | | 7.74004865 | 13.7896977 | 0.00020465 | 0.02599765 |
| Lama2 | 3.29329663 | | 5.43608566 | 13.7989457 | 0.00020364 | 0.02599765 |
| Cd300lb | -1.7380849 | | 7.52193805 | 13.7680067 | 0.00020702 | 0.02599765 |
| Gsn | -1.4376859 | | 8.21346788 | 13.6044268 | 0.00022586 | 0.02761232 |
| Rasa2 | -1.7228832 | | 7.54026387 | 13.6131223 | 0.00022482 | 0.02761232 |
| Chil3 | -5.6964337 | | 5.76074525 | 13.5037371 | 0.0002383 | 0.02888888 |
| Atg7 | -1.5964092 | | 7.72791455 | 13.4102722 | 0.00025047 | 0.0301111 |
| Rhoj | 1.09391884 | | 9.01026205 | 13.3900478 | 0.00025319 | 0.03018589 |
| Pla2g7 | -1.4609373 | | 8.06496393 | 13.3680507 | 0.00025617 | 0.03029158 |
| 4833407H14Rik | -2.4893431 | | 6.61939139 | 13.3307917 | 0.00026131 | 0.03064807 |
| Mrpl33 | -1.2841502 | | 8.57129935 | 13.2834653 | 0.00026799 | 0.03097961 |
| Pik3ap1 | -1.0521875 | | 9.45715334 | 13.280364 | 0.00026844 | 0.03097961 |
| Cers6 | -2.0089886 | | 7.09855543 | 13.2420274 | 0.00027398 | 0.03110778 |
| Alox5ap | -1.2918616 | | 8.54630712 | 13.213565 | 0.00027817 | 0.03110778 |
| Slc8a1 | 1.61004094 | | 7.08244657 | 13.2343758 | 0.0002751 | 0.03110778 |
| Pstpip2 | -1.8893797 | | 7.25078425 | 13.2147312 | 0.000278 | 0.03110778 |
| Snx18 | -1.5357669 | | 7.84768672 | 13.1852216 | 0.00028241 | 0.03133877 |
| Scrg1 | -5.6862191 | | 5.73023623 | 13.164414 | 0.00028556 | 0.03144671 |
| Ifi44 | 1.97915621 | | 6.43916818 | 12.8843916 | 0.00033161 | 0.03569986 |
| Skap2 | -1.4102503 | | 8.10817361 | 12.8867433 | 0.00033119 | 0.03569986 |
| Alox5 | -3.6122019 | | 6.10935903 | 12.8969516 | 0.00032939 | 0.03569986 |
| Nfe2l2 | -1.0917703 | | 9.19998493 | 12.8562006 | 0.00033664 | 0.03597314 |
| Klf10 | -1.5017972 | | 7.92714645 | 12.7874355 | 0.00034924 | 0.03704514 |
| Il18rap | -1.9164568 | | 7.14924585 | 12.730851 | 0.00035996 | 0.03781588 |
| Tpd52 | -1.0256813 | | 9.46290213 | 12.7215989 | 0.00036175 | 0.03781588 |
| Selplg | -1.4969056 | | 7.87268818 | 12.6136525 | 0.00038324 | 0.0397747 |
| Rrad | -2.9780056 | | 6.2735089 | 12.4969731 | 0.00040793 | 0.04144233 |
| Smad3 | -1.8285662 | | 7.29999803 | 12.5205752 | 0.00040281 | 0.04144233 |
| Cnnm2 | -2.0508361 | | 6.94797512 | 12.4311525 | 0.00042256 | 0.04262817 |
| Klra2 | -1.6540952 | | 7.47522281 | 12.3855837 | 0.00043299 | 0.04313158 |
| Fyb | -1.3770092 | | 8.1088548 | 12.3832819 | 0.00043353 | 0.04313158 |
| Gab2 | -1.0704477 | | 9.17919885 | 12.3507902 | 0.00044114 | 0.04358799 |
| Irs2 | -3.5794077 | | 6.04717298 | 12.2820383 | 0.00045769 | 0.04491541 |
| Padi2 | -3.5959839 | | 6.04265424 | 12.2574949 | 0.00046374 | 0.0452024 |
| Oasl2 | 1.43249405 | | 7.50353038 | 12.1343757 | 0.00049538 | 0.04796161 |
| Atp1a3 | -5.5622675 | | 5.65087745 | 12.0961655 | 0.00050563 | 0.04862796 |
| **Macrophages** | | | | | | |
| **gene** | **logFC** | | **logCPM** | **F** | **pval** | **padj** |
| Fn1 | 2.26916826 | | 6.60649164 | 108.740401 | 1.93E-25 | 3.61E-21 |
| Igkc | 2.46107464 | | 5.50583753 | 80.2407526 | 3.39E-19 | 3.17E-15 |
| Dcn | 1.50667659 | | 9.35554359 | 71.5257516 | 2.79E-17 | 1.74E-13 |
| Gsn | 1.48958413 | | 9.62241291 | 70.8479198 | 3.93E-17 | 1.84E-13 |
| Il10 | -2.1131032 | | 6.06715069 | 66.9918292 | 2.77E-16 | 1.04E-12 |
| Ccl5 | -1.8471353 | | 6.3119329 | 61.091595 | 5.52E-15 | 1.72E-11 |
| Vcam1 | -1.6047949 | | 6.81684579 | 59.0067493 | 1.59E-14 | 4.26E-11 |
| Hsph1 | -1.3839242 | | 8.33807201 | 56.2025175 | 6.61E-14 | 1.55E-10 |
| Hspb1 | -1.2963305 | | 9.89943758 | 54.5252528 | 1.55E-13 | 3.23E-10 |
| Dnaja1 | -1.2347009 | | 10.6095678 | 50.669178 | 1.10E-12 | 2.06E-09 |
| Irf7 | 1.51575804 | | 6.45164406 | 50.3137076 | 1.32E-12 | 2.06E-09 |
| Zbtb16 | -1.8073726 | | 5.90566133 | 50.3555005 | 1.29E-12 | 2.06E-09 |
| Dpt | 1.88992392 | | 5.40714012 | 50.1077665 | 1.47E-12 | 2.12E-09 |
| Oasl2 | 1.5963221 | | 6.09131627 | 49.3891894 | 2.12E-12 | 2.83E-09 |
| Bcl2a1d | -1.4948432 | | 6.81594505 | 48.8733967 | 2.75E-12 | 3.44E-09 |
| Lum | 1.79481375 | | 5.56948233 | 48.6318498 | 3.11E-12 | 3.65E-09 |
| Lgals3bp | 1.4133609 | | 6.71069254 | 47.1466275 | 6.64E-12 | 7.32E-09 |
| Ifi27l2a | 1.17484861 | | 11.3381352 | 46.8508492 | 7.72E-12 | 8.03E-09 |
| Got1 | -1.5632932 | | 6.25627883 | 45.6315383 | 1.44E-11 | 1.42E-08 |
| Il1b | -1.115632 | | 10.5206812 | 41.2670675 | 1.34E-10 | 1.25E-07 |
| Gm13842 | -7.0276244 | | 3.29530521 | 40.6256478 | 1.85E-10 | 1.63E-07 |
| Lyz1 | 1.33755107 | | 6.56488073 | 40.5626779 | 1.92E-10 | 1.63E-07 |
| Serping1 | 1.37510083 | | 6.23841389 | 40.1688059 | 2.34E-10 | 1.91E-07 |
| Clec3b | 1.55042656 | | 5.65826689 | 40.0299688 | 2.52E-10 | 1.96E-07 |
| Nts | 2.83579731 | | 3.94907147 | 39.7490589 | 2.90E-10 | 2.18E-07 |
| Gm20513 | -1.726115 | | 5.60166894 | 39.6733106 | 3.02E-10 | 2.18E-07 |
| Prg4 | 2.13677077 | | 4.66084691 | 38.875694 | 4.54E-10 | 2.93E-07 |
| S100a4 | 1.09899327 | | 9.56750362 | 38.9107171 | 4.46E-10 | 2.93E-07 |
| Smoc2 | 1.64432759 | | 5.38550416 | 38.8796962 | 4.53E-10 | 2.93E-07 |
| Hspa1b | -1.0986361 | | 9.50041037 | 38.773006 | 4.79E-10 | 2.99E-07 |
| Ifit3 | 1.58482694 | | 5.53929125 | 38.3469239 | 5.95E-10 | 3.60E-07 |
| Cd79a | 2.55678308 | | 4.11842642 | 38.077084 | 6.84E-10 | 3.88E-07 |
| Alox15 | 7.16425943 | | 2.58677195 | 38.1020621 | 6.75E-10 | 3.88E-07 |
| Edil3 | -2.0020691 | | 5.03274323 | 37.4010828 | 9.66E-10 | 5.33E-07 |
| Ccr2 | 1.37598698 | | 6.14567183 | 37.2502793 | 1.04E-09 | 5.59E-07 |
| Saa3 | 2.76056875 | | 3.85063683 | 35.7087716 | 2.30E-09 | 1.20E-06 |
| Cxcl13 | -1.9953708 | | 4.93695976 | 35.303246 | 2.83E-09 | 1.40E-06 |
| Crispld2 | 1.45777329 | | 5.70609309 | 35.338618 | 2.78E-09 | 1.40E-06 |
| Bcl2a1b | -1.0677785 | | 9.08472232 | 35.1526349 | 3.06E-09 | 1.47E-06 |
| Cxcl14 | 1.67607274 | | 5.22937323 | 34.8054611 | 3.66E-09 | 1.71E-06 |
| Pam | 1.19581732 | | 6.68477059 | 34.4906736 | 4.30E-09 | 1.97E-06 |
| Gpr34 | 1.48841678 | | 5.62533722 | 34.0510732 | 5.39E-09 | 2.40E-06 |
| Hspd1 | -1.0941179 | | 7.8563675 | 33.5975096 | 6.81E-09 | 2.97E-06 |
| Col1a2 | 1.4606398 | | 5.51679839 | 33.515252 | 7.10E-09 | 3.02E-06 |
| Bcl2a1a | -1.3778993 | | 6.05933202 | 32.8408236 | 1.00E-08 | 4.18E-06 |
| Clec4e | -1.3434652 | | 6.16014599 | 32.443864 | 1.23E-08 | 5.02E-06 |
| Gm14221 | -2.102094 | | 4.63385623 | 31.7345948 | 1.77E-08 | 6.92E-06 |
| Tagln | 1.23615596 | | 6.28643993 | 31.2913001 | 2.23E-08 | 8.52E-06 |
| Ogn | 1.47196297 | | 5.34801311 | 30.7666293 | 2.92E-08 | 1.09E-05 |
| Col3a1 | 1.27845745 | | 5.84889247 | 30.4253923 | 3.48E-08 | 1.28E-05 |
| Ear2 | 2.07547614 | | 4.31485664 | 30.1378413 | 4.04E-08 | 1.45E-05 |
| Htra3 | 1.66085372 | | 4.87657619 | 29.8299302 | 4.73E-08 | 1.67E-05 |
| Angptl4 | 2.72083226 | | 3.64360035 | 29.4149243 | 5.86E-08 | 2.03E-05 |
| Gas1 | 1.82626537 | | 4.56428372 | 29.3708719 | 6.00E-08 | 2.04E-05 |
| Col6a1 | 1.73775643 | | 4.73441805 | 29.2449757 | 6.40E-08 | 2.14E-05 |
| Fkbp5 | -1.4945854 | | 5.47616022 | 28.8723922 | 7.75E-08 | 2.50E-05 |
| Mir155hg | -1.20125 | | 6.29263928 | 28.7565245 | 8.23E-08 | 2.60E-05 |
| Timp1 | 1.7568054 | | 4.76853343 | 28.7364272 | 8.32E-08 | 2.60E-05 |
| Myh11 | 1.24547943 | | 5.98979707 | 28.2789866 | 1.05E-07 | 3.18E-05 |
| Zdhhc14 | -1.007235 | | 7.65058968 | 27.3593989 | 1.69E-07 | 4.96E-05 |
| Col6a2 | 1.75630593 | | 4.56130684 | 27.293922 | 1.75E-07 | 5.05E-05 |
| Ddx60 | 1.29902093 | | 5.6887684 | 27.0126366 | 2.03E-07 | 5.68E-05 |
| Mmp12 | 1.08993012 | | 6.5096126 | 26.857141 | 2.20E-07 | 5.96E-05 |
| Gfpt2 | 1.28501586 | | 5.58108954 | 26.7659931 | 2.30E-07 | 6.16E-05 |
| Tmem251 | -1.2690136 | | 5.91515597 | 26.4218049 | 2.75E-07 | 7.06E-05 |
| Myoc | 4.133433 | | 2.87786062 | 25.8975105 | 3.61E-07 | 9.14E-05 |
| Islr | 1.77908393 | | 4.42017955 | 25.7948365 | 3.81E-07 | 9.51E-05 |
| Ngf | 1.81887083 | | 4.37564821 | 25.6285843 | 4.15E-07 | 0.00010227 |
| Itga9 | -1.02773 | | 6.8743835 | 25.3364338 | 4.83E-07 | 0.00011744 |
| Rcan1 | -1.01158 | | 6.99213112 | 25.0826443 | 5.50E-07 | 0.00013056 |
| Bgn | 1.01168044 | | 6.56626101 | 24.7823904 | 6.43E-07 | 0.00014879 |
| Pcdh9 | 1.65639564 | | 4.55378623 | 24.5892074 | 7.11E-07 | 0.0001605 |
| Ahsa2 | -1.5930797 | | 4.99678426 | 24.5622506 | 7.21E-07 | 0.00016083 |
| Ifi213 | 1.68811905 | | 4.52439497 | 24.0646369 | 9.34E-07 | 0.00020104 |
| Sdc2 | 1.55186104 | | 4.73976768 | 23.9896663 | 9.71E-07 | 0.00020664 |
| Ppp1r14b | 1.06936375 | | 6.25778401 | 23.9337515 | 9.99E-07 | 0.00021034 |
| Mmp13 | -1.1880786 | | 5.98782572 | 23.816321 | 1.06E-06 | 0.00022108 |
| Rarres2 | 1.30719344 | | 5.2597482 | 23.3787838 | 1.33E-06 | 0.00026855 |
| Ccdc80 | 1.45934574 | | 4.84995741 | 23.3116581 | 1.38E-06 | 0.00027259 |
| Pcolce2 | 1.8282999 | | 4.20504692 | 23.2150123 | 1.45E-06 | 0.00028327 |
| Satb1 | 1.89780727 | | 4.13718873 | 22.9551783 | 1.66E-06 | 0.00031442 |
| Abi3bp | 1.82762786 | | 4.18064239 | 22.5042652 | 2.10E-06 | 0.00038211 |
| Enpp2 | 2.79609699 | | 3.25375317 | 22.4767987 | 2.13E-06 | 0.00038388 |
| Ptx3 | 2.22094675 | | 3.69546693 | 22.4038656 | 2.21E-06 | 0.00039494 |
| Tnfaip6 | 1.17958908 | | 5.50323 | 22.3092213 | 2.33E-06 | 0.00041097 |
| Ankrd33b | -1.0467932 | | 6.3241119 | 21.9182343 | 2.85E-06 | 0.00048546 |
| Ms4a4d | 2.14828126 | | 3.72764217 | 21.5741468 | 3.41E-06 | 0.00057042 |
| Il12b | -6.1603771 | | 2.61617138 | 21.4759465 | 3.59E-06 | 0.00059162 |
| Cadm1 | -1.0988105 | | 5.99319084 | 20.8753298 | 4.91E-06 | 0.00077952 |
| Ifit2 | 1.21267449 | | 5.32011222 | 20.6859015 | 5.42E-06 | 0.00084621 |
| C3 | 1.05294673 | | 5.83408198 | 20.4850088 | 6.02E-06 | 0.00093204 |
| Ccr7 | 1.68259052 | | 4.23627603 | 19.5929275 | 9.60E-06 | 0.00140459 |
| Runx3 | -1.3482255 | | 5.10580053 | 19.4857999 | 1.01E-05 | 0.00147407 |
| Ifi208 | 2.10895459 | | 3.63565757 | 19.3884964 | 1.07E-05 | 0.00152741 |
| Chst11 | -1.0007407 | | 6.16442534 | 19.3241341 | 1.10E-05 | 0.00156779 |
| Alas2 | -6.0055181 | | 2.48543862 | 19.0330837 | 1.29E-05 | 0.0017987 |
| Slc25a33 | -1.2373401 | | 5.36458193 | 18.9789911 | 1.32E-05 | 0.0018367 |
| Phldb1 | -1.083642 | | 5.78911906 | 18.8067994 | 1.45E-05 | 0.00199537 |
| Zbp1 | 1.19759219 | | 5.24018022 | 18.7275911 | 1.51E-05 | 0.00203508 |
| Acp5 | -1.3260746 | | 5.12301585 | 18.4943269 | 1.71E-05 | 0.0022513 |
| Ighd | 3.31427235 | | 2.702387 | 18.1731363 | 2.02E-05 | 0.00264597 |
| Gpr84 | -1.2071493 | | 5.3618188 | 18.0960486 | 2.10E-05 | 0.00273612 |
| AA467197 | 1.95921259 | | 3.73215808 | 18.0507029 | 2.15E-05 | 0.00278273 |
| Tnxb | 1.21503614 | | 5.02691991 | 17.9679991 | 2.25E-05 | 0.00288635 |
| Vcan | 1.49428952 | | 4.37558677 | 17.8131635 | 2.44E-05 | 0.00310964 |
| Meg3 | 1.18286542 | | 5.05929888 | 17.6994144 | 2.59E-05 | 0.00327888 |
| Mfap5 | 1.02955466 | | 5.58909057 | 17.5966521 | 2.73E-05 | 0.00341473 |
| Ifi44 | 1.61454899 | | 4.15000061 | 17.562649 | 2.78E-05 | 0.00342052 |
| Rcn3 | 1.01547079 | | 5.77932601 | 17.5552531 | 2.79E-05 | 0.00342052 |
| Spic | -1.1171846 | | 5.49479311 | 17.4752808 | 2.91E-05 | 0.00349976 |
| Abca8a | 1.20176582 | | 5.02382904 | 17.3073256 | 3.18E-05 | 0.00379867 |
| Ppp1r14a | 1.89348519 | | 3.73217043 | 17.197031 | 3.37E-05 | 0.00397501 |
| Cd248 | 2.10739714 | | 3.45710868 | 17.1665124 | 3.43E-05 | 0.0039892 |
| Ccl11 | 2.16128213 | | 3.40736249 | 17.1050331 | 3.54E-05 | 0.00409497 |
| Col15a1 | 1.03715153 | | 5.5100128 | 17.0321351 | 3.68E-05 | 0.00420328 |
| Tcf21 | 2.64404341 | | 3.03675133 | 17.0139077 | 3.71E-05 | 0.0042181 |
| Cxcl17 | -5.8111036 | | 2.38572884 | 17.0002209 | 3.74E-05 | 0.00422301 |
| Lhfp | 1.11095807 | | 5.24845128 | 16.8629573 | 4.02E-05 | 0.00451242 |
| Prelp | 1.37525024 | | 4.50951975 | 16.7805897 | 4.20E-05 | 0.00468449 |
| Pcolce | 1.17991482 | | 4.99162082 | 16.6032733 | 4.61E-05 | 0.00508284 |
| Spp1 | -1.3156147 | | 4.95770149 | 16.3634107 | 5.23E-05 | 0.00560337 |
| Pcsk6 | 1.26118246 | | 4.69337313 | 15.9634356 | 6.46E-05 | 0.00672825 |
| Col6a3 | 1.49494165 | | 4.1658554 | 15.8990518 | 6.69E-05 | 0.00691119 |
| Fbln1 | 1.38084781 | | 4.41870404 | 15.8917262 | 6.71E-05 | 0.00691119 |
| Myl9 | 1.08805651 | | 5.2898879 | 15.7626858 | 7.19E-05 | 0.00711033 |
| Cxcl3 | -1.4467442 | | 4.64722182 | 15.7749517 | 7.14E-05 | 0.00711033 |
| Slfn1 | 2.18026022 | | 3.28699867 | 15.6849798 | 7.49E-05 | 0.00734579 |
| Adgrd1 | 2.036173 | | 3.41020073 | 15.5169425 | 8.18E-05 | 0.00794514 |
| Sox9 | 2.42685746 | | 3.07536396 | 15.5183416 | 8.18E-05 | 0.00794514 |
| Sfrp1 | 2.40831291 | | 3.08330867 | 15.4469626 | 8.49E-05 | 0.00816018 |
| Mmp2 | 1.44163914 | | 4.26195317 | 15.4288488 | 8.57E-05 | 0.00819672 |
| Ifit3b | 1.82169656 | | 3.6816365 | 15.4134928 | 8.64E-05 | 0.00822164 |
| Prtn3 | 4.40395004 | | 2.03648408 | 15.3245862 | 9.06E-05 | 0.00853105 |
| Lrg1 | 2.85561723 | | 2.74006322 | 15.2323116 | 9.51E-05 | 0.00881755 |
| Fbrs | -1.1910241 | | 5.06685733 | 15.1988891 | 9.68E-05 | 0.0088945 |
| Gpx3 | 1.025497 | | 5.34060963 | 15.0613907 | 0.00010416 | 0.00951965 |
| Siglec1 | 1.72832132 | | 3.79537651 | 14.9500426 | 0.00011049 | 0.01000056 |
| Dclk1 | 1.90337534 | | 3.50013357 | 14.9048473 | 0.00011317 | 0.01014461 |
| 4732471J01Rik | -1.5414211 | | 4.21495856 | 14.8576814 | 0.00011603 | 0.01027541 |
| Cygb | 1.13264473 | | 4.94076359 | 14.7257892 | 0.00012444 | 0.01084386 |
| Map2k3os | -1.3464677 | | 4.58732578 | 14.5672613 | 0.00013535 | 0.01163287 |
| Ms4a4c | 1.28934935 | | 4.50973862 | 14.4018633 | 0.00014777 | 0.01236009 |
| Cytl1 | 2.39747906 | | 3.00171629 | 14.2767174 | 0.00015793 | 0.0129777 |
| Oas3 | 1.80789537 | | 3.60383027 | 14.2016134 | 0.00016436 | 0.01333057 |
| Ankrd1 | -1.0876222 | | 5.18342543 | 14.2089557 | 0.00016372 | 0.01333057 |
| Gask1b | 1.20201416 | | 4.69436169 | 14.1919927 | 0.0001652 | 0.01334114 |
| Spink2 | 3.58687643 | | 2.3103384 | 14.1329733 | 0.00017046 | 0.01370711 |
| Ly6d | 2.30089514 | | 3.06732995 | 14.1211769 | 0.00017153 | 0.01373437 |
| Phf11a | 1.72705193 | | 3.68331355 | 14.1076357 | 0.00017277 | 0.01377471 |
| B3galt1 | 2.23755012 | | 3.12055861 | 14.0858415 | 0.00017479 | 0.01387617 |
| Cnnm2 | -1.1915113 | | 4.88012232 | 13.9636672 | 0.00018652 | 0.0146216 |
| C1qtnf7 | 2.18060554 | | 3.13815899 | 13.8119561 | 0.00020219 | 0.0156541 |
| Col1a1 | 1.09332255 | | 4.91624832 | 13.812371 | 0.00020215 | 0.0156541 |
| Adamts12 | 2.30743165 | | 3.05240575 | 13.8037991 | 0.00020307 | 0.0156575 |
| Zmynd15 | -1.3021498 | | 4.60586427 | 13.6342839 | 0.00022225 | 0.01687127 |
| Glis3 | -1.6705697 | | 3.93256407 | 13.5767029 | 0.00022917 | 0.01717501 |
| Gm16685 | 1.14918013 | | 4.828049 | 13.5185696 | 0.00023638 | 0.01757461 |
| Nrxn1 | -2.5040309 | | 3.07743222 | 13.4971707 | 0.00023909 | 0.01770587 |
| Rian | 1.7426933 | | 3.56061858 | 13.3698705 | 0.00025587 | 0.0188002 |
| Iglc1 | 3.92003325 | | 2.18782925 | 13.3549119 | 0.00025792 | 0.0188767 |
| Col5a2 | 1.35088429 | | 4.20560618 | 13.3201098 | 0.00026275 | 0.01908117 |
| Lsamp | 1.49638007 | | 3.94035662 | 13.3204236 | 0.00026271 | 0.01908117 |
| Trim12a | 1.08364619 | | 4.93714882 | 13.2835393 | 0.00026793 | 0.0193818 |
| Ifi206 | 1.26839203 | | 4.41086919 | 13.1763451 | 0.00028369 | 0.02029483 |
| Chil3 | -3.9763465 | | 2.38511478 | 13.1121857 | 0.00029357 | 0.0208349 |
| Fgf12 | 5.16007445 | | 1.82554622 | 13.0213173 | 0.00030817 | 0.02162468 |
| A530013C23Rik | -1.2918769 | | 4.58723708 | 12.9654774 | 0.00031749 | 0.02211352 |
| Myh7 | -2.9937311 | | 2.76890504 | 12.8724503 | 0.00033367 | 0.02294147 |
| Gm15708 | -2.4421639 | | 3.06619494 | 12.8071972 | 0.00034551 | 0.02362557 |
| Erlec1 | 1.1164501 | | 4.78225608 | 12.6271001 | 0.00038043 | 0.025825 |
| Qpct | 1.34539483 | | 4.16343468 | 12.3906911 | 0.00043174 | 0.02858302 |
| Aff3 | 1.10436916 | | 4.74027403 | 12.3667746 | 0.0004373 | 0.02884949 |
| S100a2 | 5.0676644 | | 1.77283131 | 12.2529488 | 0.00046479 | 0.03004616 |
| Dpep1 | 1.06503185 | | 4.7836886 | 12.2620139 | 0.00046254 | 0.03004616 |
| Olfm1 | 1.21437539 | | 4.49142466 | 12.231057 | 0.00047028 | 0.03027877 |
| Olfr655 | -2.1027644 | | 3.25389086 | 12.1394934 | 0.00049393 | 0.03147735 |
| Cd5l | 5.25837878 | | 1.62334264 | 12.0385299 | 0.00052141 | 0.03269055 |
| 4930526L06Rik | -2.1221723 | | 3.30258634 | 12.0120176 | 0.00052888 | 0.03281169 |
| Mzb1 | 5.11746529 | | 1.6973464 | 11.9516824 | 0.00054628 | 0.03355791 |
| Serpinb1a | 1.19065025 | | 4.46315753 | 11.9383468 | 0.00055021 | 0.03362716 |
| Dmac2l | 1.97276226 | | 3.1878439 | 11.9008972 | 0.00056138 | 0.03414926 |
| Gm32004 | 4.07540079 | | 1.86453083 | 11.8405154 | 0.00057987 | 0.03493412 |
| Igfbp6 | 1.07389249 | | 4.65899396 | 11.6767364 | 0.0006332 | 0.03770915 |
| Ttpal | -1.1098288 | | 4.80857228 | 11.6411944 | 0.00064541 | 0.03826723 |
| Hspbap1 | -1.1371807 | | 4.74459414 | 11.5584209 | 0.00067478 | 0.03975699 |
| Slc15a2 | -1.2802779 | | 4.35010722 | 11.4327971 | 0.00072196 | 0.04227065 |
| Ccl17 | -1.0904273 | | 4.92043444 | 11.4119085 | 0.00073012 | 0.04261532 |
| Pdgfrl | 2.58175034 | | 2.6175801 | 11.3489156 | 0.0007553 | 0.04381211 |
| Jag1 | -1.3802526 | | 4.16678325 | 11.320621 | 0.00076689 | 0.04407529 |
| Igf2bp2 | -1.3053154 | | 4.27953452 | 11.3253866 | 0.00076493 | 0.04407529 |
| Mrm2 | -1.5781109 | | 3.8319966 | 11.2399313 | 0.00080096 | 0.04561317 |
| F7 | 4.99704847 | | 1.63646999 | 11.0498949 | 0.00088735 | 0.04962788 |
| **Monocytes** | | | | | | |
| **gene** | **logFC** | | **logCPM** | **F** | **pval** | **padj** |
| S100a9 | 2.01453031 | | 7.9543598 | 48.9139424 | 2.70E-12 | 4.43E-08 |
| Ifit1 | 2.26648736 | | 6.50713424 | 43.9206063 | 3.45E-11 | 2.83E-07 |
| Ifit3 | 2.92489249 | | 5.51217424 | 41.6132447 | 1.12E-10 | 6.13E-07 |
| Oasl1 | 2.99924177 | | 5.39072807 | 40.3198099 | 2.17E-10 | 8.90E-07 |
| Rsad2 | 1.96167824 | | 7.02803585 | 39.0373974 | 4.18E-10 | 1.37E-06 |
| Ifit2 | 2.2651183 | | 6.11733031 | 38.4518087 | 5.65E-10 | 1.54E-06 |
| Oasl2 | 2.07863477 | | 6.30673728 | 35.7892609 | 2.21E-09 | 5.18E-06 |
| Ifi206 | 3.70157593 | | 4.83629512 | 33.7960376 | 6.15E-09 | 1.26E-05 |
| Areg | -2.1983713 | | 5.44968788 | 33.5076873 | 7.13E-09 | 1.30E-05 |
| Prkg1 | 2.17393437 | | 5.89589533 | 32.6388444 | 1.11E-08 | 1.83E-05 |
| Thbs1 | -1.3682522 | | 9.86688659 | 32.0837849 | 1.48E-08 | 2.21E-05 |
| A330040F15Rik | 2.42499624 | | 5.36115142 | 29.3956844 | 5.92E-08 | 8.10E-05 |
| Isg15 | 1.47449627 | | 8.18207875 | 28.8994654 | 7.65E-08 | 9.66E-05 |
| Oas3 | 1.99852696 | | 5.85846204 | 28.4211078 | 9.79E-08 | 0.00011477 |
| Irf7 | 1.51774108 | | 7.49475675 | 27.1919248 | 1.85E-07 | 0.00020214 |
| Nrg1 | -1.4771253 | | 7.54819377 | 27.0301671 | 2.01E-07 | 0.00020604 |
| Rtp4 | 1.63818485 | | 6.44125462 | 25.066309 | 5.55E-07 | 0.00053605 |
| Phf11b | 1.70310997 | | 6.12256571 | 24.2720548 | 8.38E-07 | 0.00072413 |
| Retnlg | 6.25816973 | | 3.97084004 | 24.3586191 | 8.02E-07 | 0.00072413 |
| Lgals3bp | 1.6419672 | | 6.09255488 | 22.3547339 | 2.27E-06 | 0.00186343 |
| Cebpb | 1.11830324 | | 9.99987708 | 21.898839 | 2.88E-06 | 0.00225022 |
| Phf11d | 1.82538283 | | 5.53511551 | 20.7939595 | 5.12E-06 | 0.00382153 |
| Ifi27l2a | 1.0238506 | | 11.1486108 | 20.5695047 | 5.76E-06 | 0.00410985 |
| Slfn4 | 3.14423046 | | 4.43156145 | 20.1688683 | 7.10E-06 | 0.00485556 |
| Gpc6 | 2.57049084 | | 4.68570901 | 19.2331227 | 1.16E-05 | 0.00760513 |
| Ifi44 | 2.38817253 | | 4.80443605 | 18.9170041 | 1.37E-05 | 0.00862961 |
| Usp18 | 2.2917298 | | 4.86885662 | 18.7836586 | 1.47E-05 | 0.00891153 |
| Igfbp5 | 2.24580132 | | 4.87861974 | 18.3431126 | 1.85E-05 | 0.01045347 |
| Hpgd | -1.2407341 | | 7.16421952 | 18.4015163 | 1.79E-05 | 0.01045347 |
| Saa3 | -1.9190647 | | 4.91123964 | 18.2154061 | 1.98E-05 | 0.01080549 |
| Zbp1 | 1.28549878 | | 6.9931784 | 18.0766759 | 2.12E-05 | 0.01124692 |
| S100a8 | 1.20382048 | | 7.70250858 | 17.9868445 | 2.23E-05 | 0.01142174 |
| Hsph1 | -1.1988897 | | 7.37263174 | 17.910977 | 2.32E-05 | 0.01152589 |
| Parp14 | 1.13445275 | | 7.78107002 | 16.5161834 | 4.83E-05 | 0.0233069 |
| Hspa1b | -1.0631572 | | 8.32739833 | 16.1495559 | 5.86E-05 | 0.02747219 |
| Lyz1 | 1.60233605 | | 5.42278647 | 15.8987047 | 6.69E-05 | 0.0304919 |
| Pde7b | 1.15110624 | | 7.3728811 | 15.7932446 | 7.07E-05 | 0.03054225 |
| Xaf1 | 1.33991952 | | 6.15427946 | 15.8302911 | 6.94E-05 | 0.03054225 |
| Cxcl3 | -2.1755989 | | 4.39184104 | 15.5944258 | 7.86E-05 | 0.03305676 |
| Gbp5 | 1.9860265 | | 4.88096303 | 15.3706428 | 8.84E-05 | 0.03628031 |
| Lgals9 | 1.2082972 | | 6.70696077 | 15.1107957 | 0.00010148 | 0.04061487 |
| Retnla | 3.18148744 | | 4.02998608 | 14.7673844 | 0.00012174 | 0.04756088 |
| Trim30a | 1.07781057 | | 7.69457769 | 14.7137117 | 0.00012525 | 0.04779596 |
| **Pericytes** | | | | | | |
| **gene** | **logFC** | | **logCPM** | **F** | **pval** | **padj** |
| Ifit2 | 2.49990822 | | 7.39253384 | 130.636632 | 3.16E-30 | 5.62E-26 |
| Irf7 | 2.29710833 | | 7.523626 | 116.035101 | 4.90E-27 | 4.35E-23 |
| Ifi27l2a | 2.33020765 | | 7.20840495 | 109.129969 | 1.59E-25 | 9.39E-22 |
| Ifit3 | 2.63862708 | | 6.45790044 | 106.634399 | 5.58E-25 | 2.48E-21 |
| Zbtb16 | -2.6514695 | | 7.15318746 | 95.0581806 | 1.91E-22 | 6.79E-19 |
| Cxcl2 | -2.2970989 | | 7.44522909 | 89.0015724 | 4.06E-21 | 1.20E-17 |
| Ifit1 | 1.83412053 | | 8.17202964 | 88.3134776 | 5.74E-21 | 1.46E-17 |
| Cxcl1 | -1.679528 | | 9.30184721 | 82.4075325 | 1.13E-19 | 2.52E-16 |
| Ifi44 | 2.13950988 | | 6.7127668 | 81.2716942 | 2.01E-19 | 3.98E-16 |
| Il6 | -1.8555068 | | 7.57301059 | 65.6669357 | 5.42E-16 | 9.64E-13 |
| Gzma | 4.75904514 | | 4.40350987 | 65.0666366 | 7.35E-16 | 1.19E-12 |
| Rsad2 | 1.54803028 | | 8.21605405 | 64.7439209 | 8.66E-16 | 1.28E-12 |
| Ednrb | -1.8360167 | | 7.50601071 | 63.9725848 | 1.28E-15 | 1.75E-12 |
| Ccl2 | -1.7640356 | | 7.92292227 | 63.099314 | 1.99E-15 | 2.53E-12 |
| Sele | -1.8353456 | | 7.17882951 | 60.5644159 | 7.22E-15 | 8.55E-12 |
| Isg15 | 1.24766658 | | 9.70019795 | 53.8413209 | 2.20E-13 | 2.44E-10 |
| Bst2 | 1.1928297 | | 9.29774172 | 46.6441915 | 8.58E-12 | 8.97E-09 |
| Hsph1 | -1.2113945 | | 9.18568543 | 45.9864089 | 1.20E-11 | 1.18E-08 |
| Ccl5 | 3.06686418 | | 4.58086662 | 44.0724776 | 3.19E-11 | 2.98E-08 |
| Birc3 | -1.5370229 | | 7.29057022 | 43.7600854 | 3.74E-11 | 3.32E-08 |
| Olfr1033 | -1.3959139 | | 7.97357862 | 42.753836 | 6.25E-11 | 5.29E-08 |
| Ccl11 | -4.9601762 | | 4.69633468 | 42.2038811 | 8.28E-11 | 6.69E-08 |
| Nrxn1 | -1.4146249 | | 7.61703909 | 41.9609476 | 9.37E-11 | 7.24E-08 |
| Crispld2 | -1.8010679 | | 6.46936292 | 41.2318087 | 1.36E-10 | 1.01E-07 |
| Gbp3 | 1.53703075 | | 6.50847308 | 40.8180329 | 1.68E-10 | 1.19E-07 |
| Abi1 | -1.1759896 | | 8.94813659 | 39.9874781 | 2.57E-10 | 1.76E-07 |
| Mt2 | -1.3737944 | | 7.29638143 | 34.8856668 | 3.51E-09 | 2.23E-06 |
| Ifit3b | 2.49612727 | | 4.72976578 | 34.9395327 | 3.42E-09 | 2.23E-06 |
| Il34 | -1.5433821 | | 6.58801481 | 34.6214991 | 4.02E-09 | 2.46E-06 |
| Oasl2 | 1.48244603 | | 6.18740865 | 34.1582155 | 5.10E-09 | 3.02E-06 |
| Hspe1 | -1.0987033 | | 8.61747824 | 33.8075747 | 6.11E-09 | 3.39E-06 |
| Adamts4 | -1.0634521 | | 9.0144896 | 33.6331158 | 6.68E-09 | 3.60E-06 |
| Plcl1 | -1.1388223 | | 8.32070965 | 33.244067 | 8.16E-09 | 4.27E-06 |
| Dhrs3 | 1.37316401 | | 6.36505623 | 32.1274437 | 1.45E-08 | 7.18E-06 |
| Grm7 | -1.2295296 | | 7.48061524 | 31.2171396 | 2.32E-08 | 1.10E-05 |
| Icam1 | -1.0377921 | | 8.73702552 | 30.7701995 | 2.91E-08 | 1.33E-05 |
| Gm17268 | -2.4266965 | | 5.22618678 | 30.6467035 | 3.11E-08 | 1.38E-05 |
| Hspd1 | -1.0503794 | | 8.36574283 | 29.3582468 | 6.04E-08 | 2.62E-05 |
| Nfkb1 | -1.0598815 | | 8.44035331 | 29.0119017 | 7.22E-08 | 3.05E-05 |
| P4ha1 | -1.1300313 | | 7.87389052 | 28.9460206 | 7.47E-08 | 3.09E-05 |
| Tnfaip3 | -1.4058912 | | 6.60083633 | 28.8164874 | 7.98E-08 | 3.22E-05 |
| Gm4951 | 1.64846522 | | 5.52127511 | 28.2792057 | 1.05E-07 | 4.07E-05 |
| Btnl9 | 1.02040429 | | 8.18508899 | 28.1862786 | 1.11E-07 | 4.09E-05 |
| Phf11d | 1.25183571 | | 6.52393948 | 27.7172876 | 1.41E-07 | 4.91E-05 |
| Slfn2 | 1.28744885 | | 6.35588803 | 27.2411479 | 1.80E-07 | 6.15E-05 |
| Meg3 | -1.2422099 | | 7.10312003 | 27.1254346 | 1.91E-07 | 6.41E-05 |
| Rnd1 | -1.0286388 | | 8.28756843 | 27.0784002 | 1.96E-07 | 6.45E-05 |
| Slc10a6 | -1.5530328 | | 6.08151024 | 26.5188945 | 2.62E-07 | 8.30E-05 |
| Ifi213 | 2.26402475 | | 4.52970467 | 26.4058213 | 2.77E-07 | 8.65E-05 |
| Gm12840 | -1.1880876 | | 7.2857759 | 26.309471 | 2.92E-07 | 8.93E-05 |
| Clasp2 | -1.1637796 | | 7.24964172 | 25.484817 | 4.47E-07 | 0.00013296 |
| Ankrd33b | -1.202502 | | 7.02078529 | 25.476338 | 4.49E-07 | 0.00013296 |
| Cacybp | -1.1666903 | | 7.15911717 | 25.4294622 | 4.60E-07 | 0.00013369 |
| Stx11 | -1.6032171 | | 5.86783026 | 25.0111471 | 5.71E-07 | 0.00016116 |
| Neurl3 | -1.2636541 | | 6.67329101 | 24.9039226 | 6.04E-07 | 0.00016771 |
| Apln | 2.02763212 | | 4.71236749 | 24.6680494 | 6.83E-07 | 0.00018662 |
| Gbp7 | 1.03222184 | | 7.39153263 | 24.5395161 | 7.30E-07 | 0.00019546 |
| Il1r1 | -1.1902144 | | 6.89866822 | 24.0513537 | 9.40E-07 | 0.00023864 |
| Procr | -1.3790762 | | 6.29829135 | 23.9426784 | 9.95E-07 | 0.00024894 |
| Birc2 | -1.5561204 | | 5.87525242 | 23.5450762 | 1.22E-06 | 0.00029766 |
| Samd4 | -1.0071595 | | 7.97316679 | 23.4660786 | 1.27E-06 | 0.00030294 |
| Lgr5 | -3.2017842 | | 4.43071267 | 23.2798016 | 1.40E-06 | 0.00031975 |
| Tnip1 | -1.387131 | | 6.17303983 | 23.3046165 | 1.39E-06 | 0.00031975 |
| Lin7a | -1.1340759 | | 6.99977171 | 22.9098054 | 1.70E-06 | 0.00038267 |
| Tmem33 | -1.235199 | | 6.54602483 | 22.696504 | 1.90E-06 | 0.00042223 |
| Hspa4l | -1.5938866 | | 5.6960746 | 22.5746129 | 2.03E-06 | 0.00043064 |
| Zhx2 | -1.2350725 | | 6.57755273 | 22.5648291 | 2.04E-06 | 0.00043064 |
| Kctd12b | 1.20316736 | | 6.24105259 | 22.6098403 | 1.99E-06 | 0.00043064 |
| Timp4 | -1.0811076 | | 7.25181015 | 22.4045823 | 2.21E-06 | 0.00046259 |
| Kalrn | -1.026444 | | 7.56838637 | 22.2219787 | 2.43E-06 | 0.0005028 |
| Ubash3b | -1.0547388 | | 7.34356542 | 21.9917475 | 2.74E-06 | 0.00056033 |
| Sgms2 | -2.8783876 | | 4.54492694 | 21.8753206 | 2.91E-06 | 0.00058199 |
| Dennd2a | -1.2797463 | | 6.31496436 | 21.8840611 | 2.90E-06 | 0.00058199 |
| Usp18 | 1.52675413 | | 5.29757779 | 21.623334 | 3.32E-06 | 0.00064907 |
| Rab20 | -1.4061699 | | 5.97391966 | 21.3574197 | 3.82E-06 | 0.00073748 |
| Alkal2 | -1.3445665 | | 6.12131074 | 21.1508316 | 4.25E-06 | 0.00080511 |
| Pde8b | -1.1059421 | | 6.79532169 | 21.090855 | 4.39E-06 | 0.00082072 |
| Ror1 | -1.0957831 | | 6.94775824 | 21.0263751 | 4.54E-06 | 0.00083996 |
| Cfap69 | -1.7048902 | | 5.41877668 | 20.9885945 | 4.63E-06 | 0.00084785 |
| Nrep | 1.66002102 | | 5.02125935 | 20.6355309 | 5.56E-06 | 0.00099883 |
| Eps8l2 | 1.76395696 | | 4.8686499 | 20.3729243 | 6.38E-06 | 0.00112298 |
| Slc15a2 | -2.3812387 | | 4.73761431 | 20.3900966 | 6.33E-06 | 0.00112298 |
| Dpt | 1.97086212 | | 4.54021777 | 20.3513804 | 6.45E-06 | 0.00112456 |
| Rel | -1.0557985 | | 6.96389629 | 20.226115 | 6.89E-06 | 0.00116632 |
| Ism1 | 1.75968458 | | 4.7847219 | 20.0097073 | 7.72E-06 | 0.00129368 |
| Xaf1 | 1.00695104 | | 6.86050761 | 19.9878491 | 7.81E-06 | 0.00129632 |
| Ucp2 | 1.2137544 | | 5.91772622 | 19.9444115 | 7.98E-06 | 0.00131382 |
| Lgals3bp | 1.21017122 | | 5.92232036 | 19.7517073 | 8.83E-06 | 0.00140123 |
| Ahsa1 | -1.1513824 | | 6.52893133 | 19.7763471 | 8.72E-06 | 0.00140123 |
| Ifi206 | 3.47215103 | | 3.39483426 | 19.7087593 | 9.03E-06 | 0.00140792 |
| Egfr | -1.5807529 | | 5.46659394 | 19.098754 | 1.24E-05 | 0.0018719 |
| Agrn | 1.2018971 | | 5.81850568 | 18.5679443 | 1.64E-05 | 0.00241106 |
| Ankrd1 | -1.1368652 | | 6.39427331 | 18.3741763 | 1.82E-05 | 0.00261624 |
| Fancc | -1.749015 | | 5.17439793 | 18.3308619 | 1.86E-05 | 0.00262203 |
| Rian | -1.7828624 | | 5.1362261 | 18.1781127 | 2.01E-05 | 0.00279711 |
| Tifa | -1.4587601 | | 5.5481901 | 17.6085891 | 2.72E-05 | 0.00360719 |
| Ifi208 | 1.96856591 | | 4.27888376 | 16.6796215 | 4.43E-05 | 0.00570472 |
| Prkca | -1.1064351 | | 6.38050863 | 16.5459549 | 4.75E-05 | 0.00607713 |
| 5330438D12Rik | -2.2875209 | | 4.5318576 | 16.3521834 | 5.27E-05 | 0.00649727 |
| Carmil1 | -1.4517476 | | 5.49432872 | 16.3270344 | 5.34E-05 | 0.00653864 |
| Gas1 | -1.1350673 | | 6.17753971 | 16.2960907 | 5.42E-05 | 0.00660074 |
| Lrrc4c | -1.2040055 | | 5.88061825 | 15.6468872 | 7.64E-05 | 0.00881716 |
| Ighm | 2.11335148 | | 4.04476451 | 15.6134361 | 7.78E-05 | 0.00885943 |
| Cyp26b1 | 1.49631112 | | 4.83972416 | 15.322097 | 9.07E-05 | 0.01007719 |
| Pla1a | -1.3636983 | | 5.50225924 | 15.1297833 | 0.00010046 | 0.01095184 |
| Map3k6 | -1.64729 | | 5.05184415 | 14.9562872 | 0.00011013 | 0.01165402 |
| Pibf1 | -1.1226076 | | 6.03464185 | 14.9554318 | 0.00011018 | 0.01165402 |
| Nfkbie | -1.691588 | | 4.94269288 | 14.7558856 | 0.00012247 | 0.01265298 |
| Cmpk2 | 1.12054478 | | 5.63760547 | 14.6109296 | 0.00013226 | 0.01358516 |
| 1110046J04Rik | -1.6609461 | | 4.96853615 | 14.2040419 | 0.00016415 | 0.01620528 |
| Slc24a2 | 2.12923841 | | 3.87930126 | 14.1121471 | 0.00017237 | 0.01673734 |
| Oasl1 | 1.07675754 | | 5.69960094 | 13.9560195 | 0.00018728 | 0.01794091 |
| Ddx60 | 1.21651235 | | 5.30699876 | 13.9509541 | 0.00018779 | 0.01794091 |
| Runx1 | -1.3683343 | | 5.40873114 | 13.7805224 | 0.00020561 | 0.01936817 |
| Lsmem1 | -1.455502 | | 5.20017875 | 13.7485525 | 0.00020914 | 0.01955716 |
| Ssbp2 | -1.0862287 | | 5.97632927 | 13.6944977 | 0.00021525 | 0.01992146 |
| Plagl1 | -1.6754475 | | 4.86915412 | 13.4855176 | 0.00024059 | 0.02159216 |
| P4ha2 | -1.3594345 | | 5.34699456 | 13.3551133 | 0.0002579 | 0.0228008 |
| Hist3h2ba | 2.57593161 | | 3.45622173 | 13.2727517 | 0.00026948 | 0.02370651 |
| Ace2 | -1.4327491 | | 5.17052057 | 13.1915336 | 0.00028141 | 0.02463415 |
| Keap1 | 1.2347068 | | 5.17721026 | 13.101243 | 0.0002953 | 0.02559787 |
| Astn2 | -2.1041097 | | 4.38965046 | 12.9708795 | 0.00031659 | 0.02730963 |
| Col14a1 | 3.48653521 | | 2.98882036 | 12.8914996 | 0.0003303 | 0.02821915 |
| Ahsa2 | -1.0444952 | | 6.03338289 | 12.7382266 | 0.00035849 | 0.02990808 |
| Nosip | 1.13634658 | | 5.37648762 | 12.6801448 | 0.0003698 | 0.03070724 |
| Snapc1 | -1.4025139 | | 5.19322489 | 12.6527486 | 0.00037526 | 0.03101549 |
| Zbp1 | 1.85684134 | | 4.04534319 | 12.3779608 | 0.0004347 | 0.03495322 |
| B430010I23Rik | -1.7811875 | | 4.6417091 | 12.1997789 | 0.00047824 | 0.03762525 |
| Tnfaip8l1 | -1.2589417 | | 5.36349261 | 11.9881166 | 0.00053572 | 0.04050981 |
| Trim34a | 1.31266834 | | 4.87180941 | 11.902027 | 0.00056105 | 0.04189047 |
| Inpp1 | 1.07646108 | | 5.42255259 | 11.8914295 | 0.00056425 | 0.04195317 |
| Hspe1-rs1 | -3.4505406 | | 3.53784454 | 11.7561419 | 0.00060677 | 0.04437174 |
| Nthl1 | -5.2730478 | | 3.18357182 | 11.7480127 | 0.00060943 | 0.04438328 |
| Angptl4 | 1.19958015 | | 5.09128851 | 11.7155213 | 0.00062016 | 0.04498048 |
| Alpk1 | -1.2968354 | | 5.27159262 | 11.589578 | 0.00066359 | 0.04679368 |
| Ikzf4 | -1.1967358 | | 5.44210141 | 11.5267112 | 0.00068641 | 0.04783319 |
| Schwann cells | | | | | | |
| **gene** | **logFC** | | **logCPM** | **F** | **pval** | **padj** |
| S100a8 | 5.29685299 | | 6.62805149 | 62.0148899 | 3.47E-15 | 5.11E-11 |
| Selp | -7.0075982 | | 6.70892352 | 35.032024 | 3.26E-09 | 2.41E-05 |
| Rsad2 | 1.9896451 | | 7.42823546 | 24.8814719 | 6.11E-07 | 0.00300638 |
| Igfbp5 | -4.3659169 | | 6.55807128 | 23.5741055 | 1.21E-06 | 0.00444408 |
| Angptl4 | 2.66080047 | | 6.46376127 | 22.9984194 | 1.63E-06 | 0.00479527 |
| Fmo1 | 2.07915661 | | 7.11016601 | 22.467592 | 2.14E-06 | 0.00526687 |
| Cyp4b1 | 2.04784415 | | 6.96167612 | 20.4563965 | 6.11E-06 | 0.01288144 |
| Zbtb16 | -2.9318147 | | 6.88585648 | 20.1867461 | 7.04E-06 | 0.01297642 |
| Mgp | -1.6317291 | | 9.26139821 | 19.5639613 | 9.75E-06 | 0.01437743 |
| Runx1 | -2.9036416 | | 6.83293085 | 19.5827288 | 9.65E-06 | 0.01437743 |
| Maff | -2.8504717 | | 6.79185009 | 18.6579945 | 1.57E-05 | 0.01925692 |
| Ifit1 | 1.63912746 | | 7.65435404 | 18.8092104 | 1.45E-05 | 0.01925692 |
| Emp1 | -1.5888473 | | 9.20621948 | 18.2714476 | 1.92E-05 | 0.02021626 |
| Iqgap2 | -1.6758166 | | 9.05596627 | 18.328423 | 1.86E-05 | 0.02021626 |
| Ccl21a | -3.3831439 | | 6.47401698 | 17.8410693 | 2.41E-05 | 0.0236527 |
| Cxcl9 | 1.67737731 | | 7.46252939 | 17.5599048 | 2.79E-05 | 0.02570549 |
| Ptprj | -1.8216788 | | 7.80102815 | 17.257574 | 3.27E-05 | 0.02836312 |
| S100a9 | 2.17943109 | | 6.44574417 | 16.3154316 | 5.37E-05 | 0.0440046 |
| Lgals3 | -1.692182 | | 7.90420151 | 16.1613058 | 5.82E-05 | 0.04522072 |
| Usp18 | 3.63980084 | | 5.41789131 | 15.9314458 | 6.58E-05 | 0.04850293 |
| **Smooth muscle cells** | | | | | | |
| **gene** | **logFC** | | **logCPM** | **F** | **pval** | **padj** |
| Lyz2 | 3.55000249 | | 7.58428148 | 208.29164 | 3.80E-47 | 6.56E-43 |
| Plac8 | 4.62798568 | | 5.3493148 | 126.526231 | 2.50E-29 | 2.16E-25 |
| Ifi27l2a | 2.41125379 | | 7.51353147 | 114.735327 | 9.44E-27 | 5.43E-23 |
| S100a9 | -6.2131339 | | 5.46026327 | 105.31027 | 1.09E-24 | 4.70E-21 |
| Rsad2 | 2.31503195 | | 6.9678789 | 95.4032026 | 1.61E-22 | 5.55E-19 |
| Ifit3 | 2.74969982 | | 5.89338636 | 87.7384542 | 7.69E-21 | 2.21E-17 |
| Ifit1 | 2.00817597 | | 7.70779937 | 85.8622319 | 1.98E-20 | 4.89E-17 |
| Ifit2 | 2.38605587 | | 6.12476593 | 79.4694738 | 5.01E-19 | 1.08E-15 |
| S100a4 | 2.07276803 | | 6.54988333 | 66.8071222 | 3.04E-16 | 5.84E-13 |
| Irf7 | 2.02378155 | | 6.47832994 | 65.2523251 | 6.70E-16 | 1.16E-12 |
| Nfkb1 | -1.5516241 | | 8.75461445 | 58.7573363 | 1.81E-14 | 2.84E-11 |
| Procr | -1.7314372 | | 7.35021044 | 55.460156 | 9.65E-14 | 1.28E-10 |
| Ifi44 | 2.09268279 | | 5.88485538 | 55.5347298 | 9.29E-14 | 1.28E-10 |
| Mt2 | -1.4601292 | | 8.8125509 | 52.7692701 | 3.79E-13 | 4.68E-10 |
| Gm20186 | -1.9967678 | | 6.34034227 | 51.6117155 | 6.83E-13 | 7.87E-10 |
| Elovl5 | -1.4334644 | | 8.22573439 | 47.9358468 | 4.44E-12 | 4.80E-09 |
| S100a8 | -3.6393264 | | 4.79235058 | 44.8549693 | 2.14E-11 | 2.17E-08 |
| 1110046J04Rik | -1.8107643 | | 6.385372 | 44.0943515 | 3.15E-11 | 3.02E-08 |
| Slfn2 | 1.74688316 | | 6.0407135 | 43.4012673 | 4.49E-11 | 4.08E-08 |
| Zbtb16 | -1.7963966 | | 6.41136689 | 42.6671586 | 6.53E-11 | 5.64E-08 |
| Zhx2 | -1.4660701 | | 7.40315506 | 41.7670041 | 1.04E-10 | 8.51E-08 |
| Lgals3bp | 2.12247054 | | 5.29362794 | 41.5298482 | 1.17E-10 | 9.17E-08 |
| Il1b | 2.3252052 | | 5.05156084 | 41.1930676 | 1.39E-10 | 9.99E-08 |
| Ms4a6c | 5.3077577 | | 3.65289944 | 41.263605 | 1.34E-10 | 9.99E-08 |
| Ccl4 | -3.8174552 | | 4.58510533 | 40.6938139 | 1.79E-10 | 1.24E-07 |
| Ly6a | 1.2063308 | | 9.56533313 | 40.4178549 | 2.06E-10 | 1.37E-07 |
| Hsph1 | -1.3684224 | | 7.65464233 | 40.0832398 | 2.45E-10 | 1.51E-07 |
| Tyrobp | 1.94201734 | | 5.51324265 | 40.1118802 | 2.41E-10 | 1.51E-07 |
| Ucp2 | 1.97715216 | | 5.46057 | 39.6700528 | 3.03E-10 | 1.80E-07 |
| Bst2 | 1.2442984 | | 8.19689208 | 37.5297039 | 9.05E-10 | 5.21E-07 |
| Cxcl1 | -1.2066567 | | 8.73772892 | 35.9544578 | 2.03E-09 | 1.13E-06 |
| Neat1 | -1.159632 | | 9.09422432 | 35.571054 | 2.47E-09 | 1.33E-06 |
| H2-Q7 | 1.49416385 | | 6.25909953 | 34.8096719 | 3.65E-09 | 1.91E-06 |
| Alox5ap | 3.44283761 | | 3.94434848 | 34.0491961 | 5.40E-09 | 2.74E-06 |
| Cd74 | 1.25011119 | | 7.52421749 | 33.6076127 | 6.77E-09 | 3.25E-06 |
| Cybb | 3.50215649 | | 3.93032837 | 33.6061682 | 6.78E-09 | 3.25E-06 |
| Adamts9 | -1.0998342 | | 9.56121138 | 33.3756045 | 7.63E-09 | 3.56E-06 |
| Dock2 | 2.39362946 | | 4.63997678 | 33.2947322 | 7.95E-09 | 3.62E-06 |
| Trmt61b | -1.1650906 | | 8.22012557 | 32.3543375 | 1.29E-08 | 5.71E-06 |
| Ifi213 | 4.25340222 | | 3.58330092 | 30.3948067 | 3.54E-08 | 1.53E-05 |
| Plagl1 | -1.3199007 | | 6.86954737 | 30.3301951 | 3.66E-08 | 1.54E-05 |
| Chil3 | 6.51926495 | | 3.09957945 | 30.1901898 | 3.93E-08 | 1.58E-05 |
| Slc6a6 | 1.11369983 | | 8.18431365 | 30.2088977 | 3.89E-08 | 1.58E-05 |
| Ifitm6 | 4.00373758 | | 3.57144475 | 29.796017 | 4.82E-08 | 1.89E-05 |
| Cpm | -1.6837712 | | 5.7349285 | 29.5164042 | 5.56E-08 | 2.14E-05 |
| Ifit3b | 2.58535345 | | 4.24701192 | 28.5486272 | 9.17E-08 | 3.44E-05 |
| Sema3g | 1.31356654 | | 6.43934708 | 28.3245098 | 1.03E-07 | 3.78E-05 |
| Gbp7 | 1.26543499 | | 6.50308779 | 27.5041067 | 1.57E-07 | 5.66E-05 |
| Samsn1 | 2.65837091 | | 4.12228095 | 27.3799579 | 1.68E-07 | 5.79E-05 |
| Gbp3 | 1.58305486 | | 5.4573166 | 27.2702826 | 1.77E-07 | 5.89E-05 |
| Cd52 | 1.67738663 | | 5.29593606 | 27.1887111 | 1.85E-07 | 6.03E-05 |
| Mpeg1 | 4.84800434 | | 3.28167362 | 26.76972 | 2.30E-07 | 7.35E-05 |
| Ptpn2 | -1.2836924 | | 6.68214545 | 26.5228509 | 2.61E-07 | 8.05E-05 |
| 1200007C13Rik | -1.964193 | | 5.149806 | 26.3545598 | 2.85E-07 | 8.63E-05 |
| Pde10a | -1.4395657 | | 6.05692694 | 25.9928519 | 3.44E-07 | 0.00010232 |
| Ankrd13c | -1.1869047 | | 7.01187761 | 25.5590622 | 4.30E-07 | 0.00012592 |
| Gdf15 | 1.23380154 | | 6.61779162 | 25.5116912 | 4.41E-07 | 0.0001269 |
| Ccr2 | 6.24684162 | | 2.97666219 | 25.2738696 | 4.99E-07 | 0.00013789 |
| Phf11d | 1.29132477 | | 6.11726858 | 25.257263 | 5.03E-07 | 0.00013789 |
| Lgals3 | 1.88599888 | | 4.83890005 | 24.5749383 | 7.16E-07 | 0.00018749 |
| Lsp1 | 2.40358394 | | 4.19888571 | 24.327886 | 8.14E-07 | 0.0002074 |
| Fas | -1.1076432 | | 7.35191953 | 24.3228883 | 8.17E-07 | 0.0002074 |
| 9930111J21Rik2 | 1.15873835 | | 6.6293212 | 24.0809823 | 9.26E-07 | 0.00023174 |
| Stmn2 | 1.01316271 | | 7.93796234 | 24.0508142 | 9.40E-07 | 0.00023204 |
| Usp37 | -1.0236956 | | 7.91138847 | 24.0191859 | 9.56E-07 | 0.00023255 |
| Fcer1g | 1.43802203 | | 5.57649254 | 23.5347807 | 1.23E-06 | 0.00029089 |
| Ccl3 | -6.0867348 | | 3.57711865 | 23.257558 | 1.42E-06 | 0.0003227 |
| Coro1a | 1.9792066 | | 4.59341867 | 22.8776317 | 1.73E-06 | 0.0003838 |
| Phf11b | 2.85176358 | | 3.79237448 | 22.6976421 | 1.90E-06 | 0.00041537 |
| Rflnb | 1.02804664 | | 7.38047035 | 22.6137375 | 1.98E-06 | 0.00042848 |
| Ighm | 2.00874844 | | 4.49868158 | 22.4569213 | 2.15E-06 | 0.00045917 |
| Chordc1 | -1.2745876 | | 6.16230149 | 22.2301739 | 2.42E-06 | 0.00049824 |
| Lgals9 | 1.17239099 | | 6.3220361 | 22.2330013 | 2.42E-06 | 0.00049824 |
| Abtb2 | -1.0115312 | | 7.6009503 | 21.8813275 | 2.91E-06 | 0.00057691 |
| Ss18l1 | -1.2165775 | | 6.37697543 | 21.776251 | 3.07E-06 | 0.00059568 |
| Mgst1 | 1.52354512 | | 5.21297509 | 21.6576137 | 3.27E-06 | 0.00061974 |
| Gm4951 | 1.78761376 | | 4.72670272 | 21.6713433 | 3.24E-06 | 0.00061974 |
| Cytip | 2.09483401 | | 4.35472538 | 21.2304257 | 4.08E-06 | 0.00074174 |
| Ctss | 2.18916275 | | 4.25257286 | 21.2505446 | 4.04E-06 | 0.00074174 |
| Alpl | 1.15887942 | | 6.26930994 | 21.195085 | 4.16E-06 | 0.00074767 |
| Nrip3 | -1.2478005 | | 6.10722224 | 20.9110254 | 4.82E-06 | 0.00085817 |
| Sntb2 | -1.0072548 | | 7.40480886 | 20.5732594 | 5.75E-06 | 0.00101321 |
| Igkc | 1.15004887 | | 6.07254789 | 20.2918326 | 6.66E-06 | 0.00115019 |
| Rtp4 | 1.1511446 | | 6.1180086 | 20.2012136 | 6.98E-06 | 0.00118231 |
| Ntrk2 | -1.0113663 | | 7.21234808 | 20.1100714 | 7.32E-06 | 0.00121937 |
| Nfkbie | -1.4378484 | | 5.57678381 | 20.105019 | 7.34E-06 | 0.00121937 |
| Lst1 | 2.26198342 | | 4.08834543 | 20.067088 | 7.49E-06 | 0.00123194 |
| Carmil1 | -1.7650742 | | 4.97190019 | 19.8209954 | 8.52E-06 | 0.00134969 |
| Ear2 | 5.75632917 | | 2.87159169 | 19.8385836 | 8.44E-06 | 0.00134969 |
| Snrnp48 | -1.3496109 | | 5.71472919 | 19.7120627 | 9.02E-06 | 0.00141584 |
| Ube2m | 1.04312685 | | 6.66724354 | 19.425521 | 1.05E-05 | 0.00161551 |
| Laptm5 | 1.91283133 | | 4.41925744 | 19.3564689 | 1.09E-05 | 0.00166015 |
| Selp | -5.8570019 | | 3.40754412 | 19.3202733 | 1.11E-05 | 0.00167707 |
| Cck | -5.920836 | | 3.32238875 | 18.9393563 | 1.35E-05 | 0.00196851 |
| Sec24a | -1.3140603 | | 5.68914365 | 18.6140025 | 1.60E-05 | 0.00228765 |
| Ifi211 | 1.58093838 | | 4.84507687 | 18.5027398 | 1.70E-05 | 0.00240524 |
| Pxn | -1.1034724 | | 6.4027909 | 18.4213157 | 1.77E-05 | 0.00248979 |
| Cmpk2 | 1.63967177 | | 4.70429645 | 18.3771266 | 1.81E-05 | 0.00252764 |
| Foxs1 | -1.0639167 | | 6.61519968 | 18.3084714 | 1.88E-05 | 0.0025994 |
| Mfsd6 | 1.40601318 | | 5.17028527 | 18.0972912 | 2.10E-05 | 0.00288107 |
| Arhgdib | 1.11907401 | | 5.96661568 | 17.7642566 | 2.50E-05 | 0.00335203 |
| Stk35 | -1.3000733 | | 5.64716211 | 17.630343 | 2.69E-05 | 0.00354154 |
| Ifi47 | 1.32682139 | | 5.2710255 | 17.4031113 | 3.03E-05 | 0.00387276 |
| Relb | -1.0609014 | | 6.45400501 | 17.2455278 | 3.29E-05 | 0.00417657 |
| Oasl2 | 1.1676095 | | 5.66944477 | 17.1975215 | 3.37E-05 | 0.00425216 |
| A730049H05Rik | -1.2381299 | | 5.78999299 | 17.1688905 | 3.42E-05 | 0.00428543 |
| Gm12185 | 1.30743242 | | 5.27496282 | 17.0465855 | 3.65E-05 | 0.00450512 |
| H2-Aa | 1.1439662 | | 5.77305441 | 17.0135479 | 3.72E-05 | 0.00452413 |
| Impact | -1.0546916 | | 6.40050424 | 16.9286041 | 3.89E-05 | 0.00466071 |
| Ptprc | 1.66405491 | | 4.57511738 | 16.6379792 | 4.53E-05 | 0.00528509 |
| Sphk1 | -1.3861082 | | 5.37069442 | 16.5628137 | 4.71E-05 | 0.00538945 |
| Tmeff2 | -1.1697691 | | 5.84448547 | 16.3758311 | 5.20E-05 | 0.00583208 |
| Blnk | 5.445001 | | 2.74651931 | 16.3067765 | 5.39E-05 | 0.00600944 |
| Gzmb | -5.6193239 | | 3.26198325 | 16.1865117 | 5.75E-05 | 0.00636212 |
| E330020D12Rik | 3.22733021 | | 3.2977407 | 16.1450697 | 5.87E-05 | 0.0064614 |
| Map2 | -1.0248481 | | 6.34735996 | 16.0561515 | 6.16E-05 | 0.00668675 |
| Bank1 | 2.02646699 | | 4.04574918 | 16.0353341 | 6.22E-05 | 0.0067184 |
| Stip1 | -1.0204988 | | 6.37682905 | 15.6725243 | 7.54E-05 | 0.00789127 |
| Cers6 | -1.0871278 | | 6.12451386 | 15.6396521 | 7.67E-05 | 0.00798123 |
| Xaf1 | 1.05323318 | | 5.9108446 | 15.6129055 | 7.78E-05 | 0.00799853 |
| Gzma | -2.8503153 | | 3.80463805 | 15.4572471 | 8.45E-05 | 0.00853246 |
| Batf | -1.1637223 | | 5.77380173 | 15.4310505 | 8.57E-05 | 0.0085348 |
| Slfn3 | 1.75743075 | | 4.31977963 | 15.3452449 | 8.96E-05 | 0.00884643 |
| Gm15987 | 2.60816116 | | 3.54966547 | 15.2687849 | 9.33E-05 | 0.00915941 |
| Arhgap15 | 1.19218666 | | 5.38887809 | 15.2569861 | 9.39E-05 | 0.00916472 |
| Ifi207 | 1.40767299 | | 4.88965614 | 15.0918071 | 0.0001025 | 0.00978131 |
| Tfcp2 | -1.201034 | | 5.61199109 | 14.9948479 | 0.0001079 | 0.01007418 |
| Fyb | 1.83439226 | | 4.18951851 | 14.984202 | 0.00010851 | 0.01007669 |
| Ms4a1 | 2.55376298 | | 3.56018674 | 14.8933775 | 0.00011386 | 0.01046104 |
| Sgms2 | -1.4491217 | | 5.05813659 | 14.8437661 | 0.0001169 | 0.01062672 |
| Cd44 | 1.32554422 | | 5.03647644 | 14.7943436 | 0.00012 | 0.01084968 |
| Syk | 2.2711685 | | 3.75253422 | 14.7840574 | 0.00012066 | 0.01084968 |
| Spi1 | 2.28115645 | | 3.73477509 | 14.5977902 | 0.00013319 | 0.01164485 |
| Ckm | -1.1119558 | | 5.76996249 | 14.6021965 | 0.00013288 | 0.01164485 |
| Tmc5 | -1.6276641 | | 4.74567275 | 14.593472 | 0.00013349 | 0.01164485 |
| Emilin2 | 3.56409077 | | 3.00435269 | 14.0711019 | 0.00017617 | 0.014421 |
| Tnfaip3 | -1.0877721 | | 5.85791183 | 14.0214371 | 0.00018088 | 0.014737 |
| Kcnn3 | -1.5916949 | | 4.74994257 | 13.9999398 | 0.00018296 | 0.01483643 |
| Edn1 | 1.2316357 | | 5.12280753 | 13.9400237 | 0.00018889 | 0.0152452 |
| Slc2a3 | -1.1589806 | | 5.57093165 | 13.8502291 | 0.00019813 | 0.01584302 |
| Man2b1 | 1.86713671 | | 4.02699099 | 13.796798 | 0.00020384 | 0.01614201 |
| Rgs1 | -3.6591849 | | 3.43359947 | 13.7210982 | 0.00021222 | 0.01658612 |
| Ednrb | -1.1467667 | | 5.57437533 | 13.5687717 | 0.00023016 | 0.01774661 |
| Psmb9 | 1.07682709 | | 5.55810298 | 13.4735134 | 0.00024214 | 0.01850526 |
| Vav3 | 1.07736473 | | 5.55399839 | 13.4292093 | 0.00024792 | 0.0188639 |
| Zfp667 | -1.7548017 | | 4.45068441 | 13.2590568 | 0.00027146 | 0.02032869 |
| Usp3 | -1.1143803 | | 5.61662334 | 13.2646459 | 0.00027066 | 0.02032869 |
| Ahsa2 | -1.1236665 | | 5.57451446 | 13.2742905 | 0.00026927 | 0.02032869 |
| Zfp61 | -1.1581973 | | 5.46792188 | 13.2463626 | 0.00027331 | 0.02034734 |
| Sec23b | -1.098277 | | 5.6770581 | 13.2253346 | 0.00027639 | 0.02040097 |
| Pcsk2 | -5.4351751 | | 3.01530165 | 13.1841946 | 0.00028252 | 0.02076492 |
| Retnlg | -5.350423 | | 3.07720813 | 13.1320811 | 0.00029049 | 0.02117025 |
| Tmem252 | -2.7182794 | | 3.69695568 | 13.1011505 | 0.00029533 | 0.02134249 |
| Syt7 | 1.32444385 | | 4.83771713 | 12.9879747 | 0.00031372 | 0.02242445 |
| Stn1 | -1.0432131 | | 5.80479791 | 12.9688609 | 0.00031694 | 0.02252727 |
| Slc4a4 | -2.6734314 | | 3.72939966 | 12.9314157 | 0.00032334 | 0.02270204 |
| Tnfsf10 | 1.4368094 | | 4.59198978 | 12.8946499 | 0.00032975 | 0.02305854 |
| Elovl6 | -1.9124353 | | 4.22977656 | 12.7191258 | 0.00036218 | 0.02512274 |
| Sp140 | 1.97214551 | | 3.83593196 | 12.6776154 | 0.00037031 | 0.02558381 |
| Mpzl1 | 1.10736754 | | 5.36014723 | 12.617465 | 0.00038241 | 0.0261069 |
| Hcls1 | 2.65059671 | | 3.32887992 | 12.5901528 | 0.00038804 | 0.02638682 |
| Ccl5 | -2.1164755 | | 4.01296921 | 12.5534061 | 0.00039575 | 0.02680516 |
| Ms4a4c | 3.03212325 | | 3.12293949 | 12.523289 | 0.00040217 | 0.02702863 |
| Sox18 | 1.05054307 | | 5.4915416 | 12.414998 | 0.00042617 | 0.02822636 |
| Oasl1 | 1.33206314 | | 4.72841186 | 12.4134268 | 0.00042653 | 0.02822636 |
| Mfap4 | -3.5362159 | | 3.34360705 | 12.0364773 | 0.00052201 | 0.0331478 |
| Ly6d | 1.76086235 | | 3.99837667 | 12.0255993 | 0.00052507 | 0.03321965 |
| Gm16685 | -2.4060232 | | 3.77212605 | 11.9449305 | 0.00054829 | 0.0340651 |
| Ifi204 | 1.36458296 | | 4.61617948 | 11.9000198 | 0.00056167 | 0.0347711 |
| Zdbf2 | -1.3799629 | | 4.82947526 | 11.8806746 | 0.00056753 | 0.03488399 |
| Apod | -2.6124185 | | 3.67532041 | 11.8829297 | 0.00056684 | 0.03488399 |
| Napsa | 2.57649796 | | 3.31943748 | 11.8704132 | 0.00057067 | 0.03492153 |
| Kctd12b | 1.18817122 | | 5.0018225 | 11.8654597 | 0.00057219 | 0.03492153 |
| Ly6c2 | 3.34714465 | | 2.92973996 | 11.7779722 | 0.00059971 | 0.03647239 |
| Ntrk3 | -1.217844 | | 5.16052918 | 11.7634161 | 0.00060442 | 0.03662969 |
| St8sia4 | 1.29093656 | | 4.72759054 | 11.6652516 | 0.00063715 | 0.03827954 |
| Zbp1 | 2.93152798 | | 3.10862341 | 11.6619299 | 0.00063829 | 0.03827954 |
| Fancc | -1.2037063 | | 5.15043908 | 11.4383469 | 0.00071984 | 0.04228917 |
| Mta1 | 1.12995332 | | 5.10278374 | 11.4075048 | 0.00073188 | 0.04285116 |
| Hp | 3.40466776 | | 2.84948798 | 11.3228191 | 0.00076602 | 0.04439833 |
| Idi1 | -1.230618 | | 5.06937517 | 11.3107806 | 0.000771 | 0.04453761 |
| Ikzf1 | 2.88813072 | | 3.10467388 | 11.2866413 | 0.00078109 | 0.04496995 |
| Smim3 | -1.4299299 | | 4.67560835 | 11.2147585 | 0.00081193 | 0.04613029 |
| Il34 | -1.11829 | | 5.3363023 | 11.1939003 | 0.0008211 | 0.04649869 |
| A230072C01Rik | -1.380203 | | 4.75123136 | 11.0918089 | 0.00086755 | 0.04794596 |
| Lcp1 | 1.63497076 | | 4.07736964 | 11.0763839 | 0.0008748 | 0.0479667 |
| Mvb12a | 1.03717817 | | 5.3298517 | 11.0621069 | 0.00088156 | 0.04818443 |
| Lncppara | -1.2183727 | | 5.06418642 | 11.0188957 | 0.00090234 | 0.04916495 |
| Gprasp2 | -5.1746011 | | 2.89502448 | 10.9707677 | 0.00092608 | 0.04983367 |
| **T cells** | | | | | | |
| **gene** | **logFC** | | **logCPM** | **F** | **pval** | **padj** |
| Cxcl2 | -3.3570175 | | 7.90335419 | 63.4170241 | 1.70E-15 | 2.07E-11 |
| H2-Eb1 | 3.53584656 | | 7.61900048 | 62.4696967 | 2.75E-15 | 2.07E-11 |
| H2-Ab1 | 3.23933808 | | 7.53917102 | 54.8814631 | 1.30E-13 | 6.50E-10 |
| Cd74 | 2.57749905 | | 9.10987525 | 45.6560795 | 1.42E-11 | 5.35E-08 |
| H2-Aa | 2.68651971 | | 7.31712261 | 39.7738259 | 2.87E-10 | 8.63E-07 |
| Ccl3 | -2.7563635 | | 6.61167558 | 35.4212234 | 2.67E-09 | 6.69E-06 |
| Ctnna3 | -4.0360875 | | 5.88771894 | 32.4380579 | 1.24E-08 | 2.66E-05 |
| Hdc | -3.5406491 | | 5.915183 | 31.0194914 | 2.57E-08 | 4.82E-05 |
| Acta2 | -2.3642528 | | 6.7662932 | 26.9895519 | 2.05E-07 | 0.00034286 |
| Adamts4 | -2.9907744 | | 6.02933841 | 26.4916165 | 2.66E-07 | 0.00039923 |
| Birc5 | 6.27988456 | | 5.11641628 | 26.2976117 | 2.94E-07 | 0.00040126 |
| Fabp3 | 1.99390229 | | 7.48860455 | 24.2030616 | 8.69E-07 | 0.00108914 |
| Gadd45b | -1.6883942 | | 7.78129201 | 19.5317783 | 9.91E-06 | 0.01146353 |
| Pnpla7 | 3.36648552 | | 5.35531346 | 18.9347918 | 1.35E-05 | 0.01394294 |
| Slc6a6 | 1.73790448 | | 7.38514218 | 18.884615 | 1.39E-05 | 0.01394294 |
| Acod1 | -5.7090663 | | 4.81590164 | 18.3458083 | 1.85E-05 | 0.01733926 |
| Ccl4 | -1.5114342 | | 8.42664325 | 17.7693352 | 2.50E-05 | 0.02208822 |
| Pln | 1.61978046 | | 7.9420153 | 16.9405948 | 3.86E-05 | 0.03225997 |
| Styk1 | -2.4589474 | | 5.66665754 | 16.0283597 | 6.25E-05 | 0.04944119 |

**Figure 1 Clustering and cell markers**


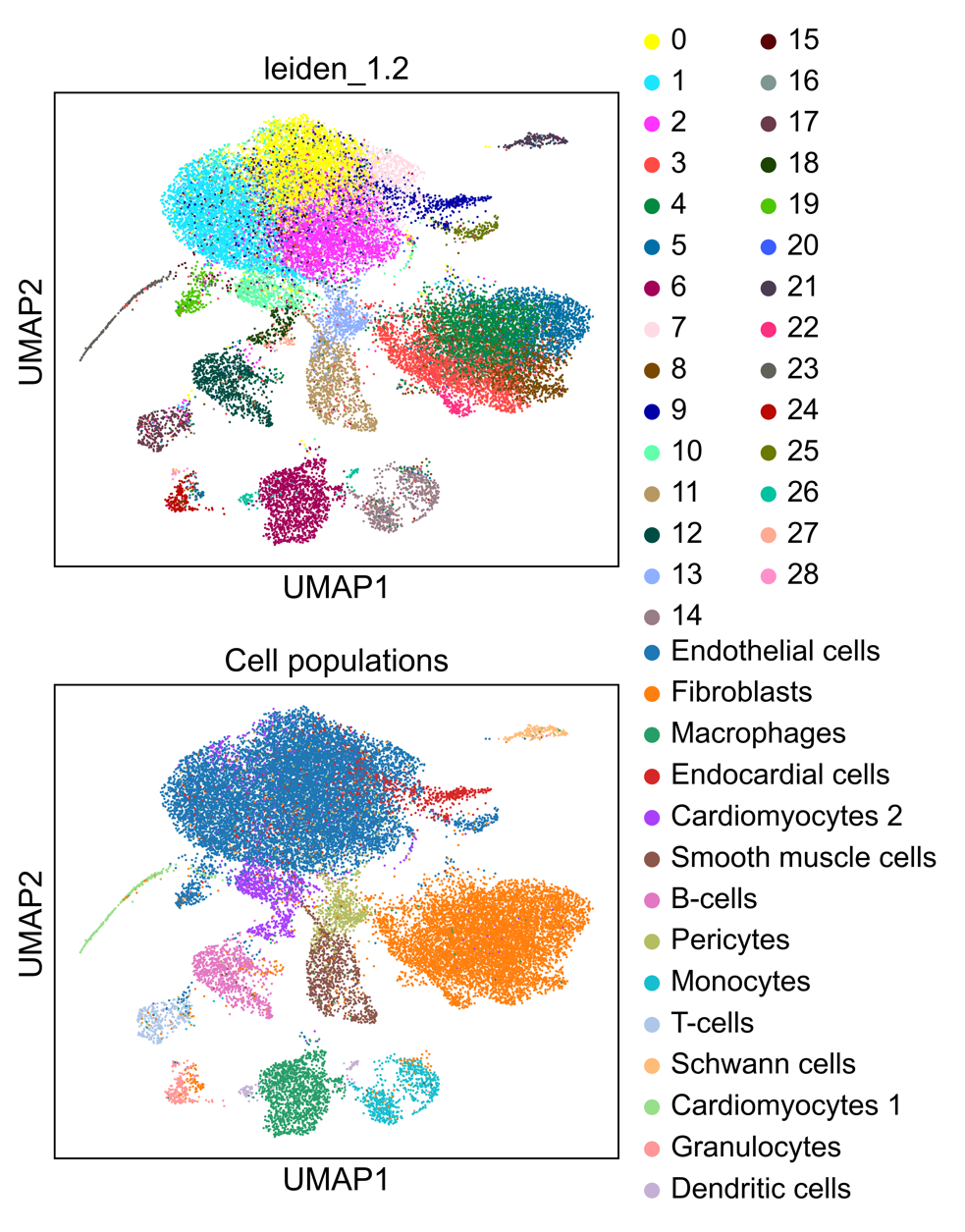


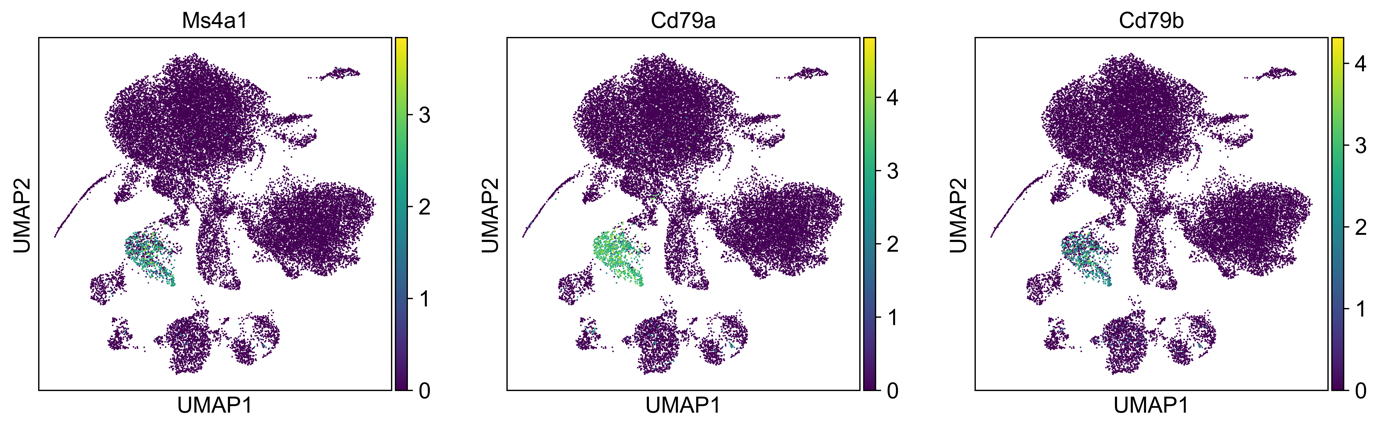


**B cells**


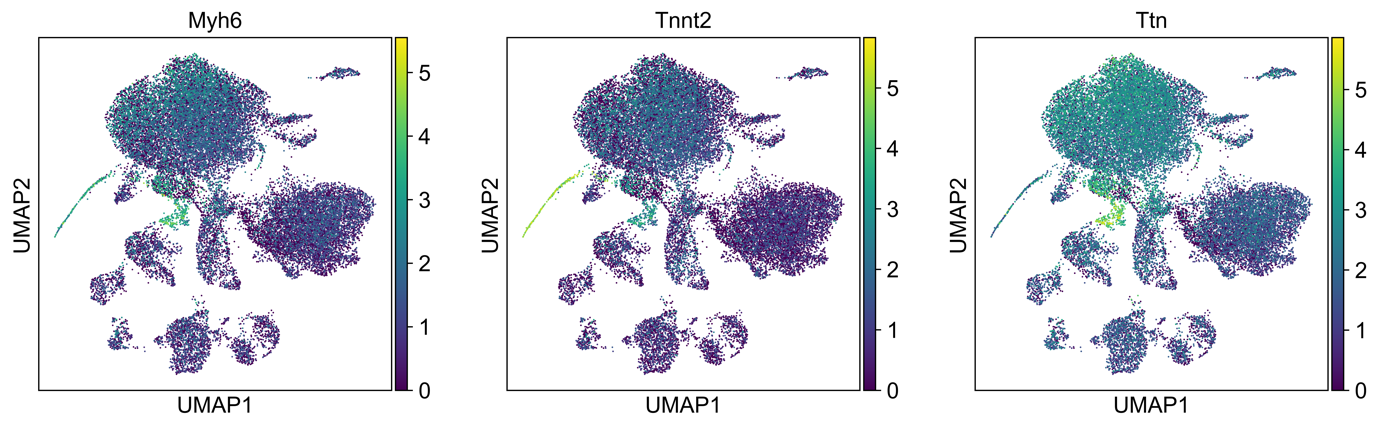


**Endocardial cells**

**Dendritic cells**

**Cardiomyocytes**


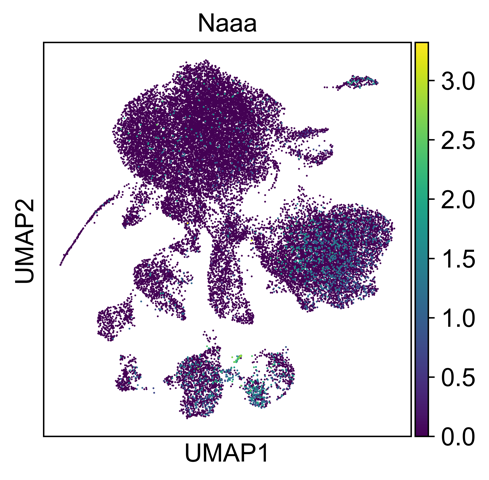

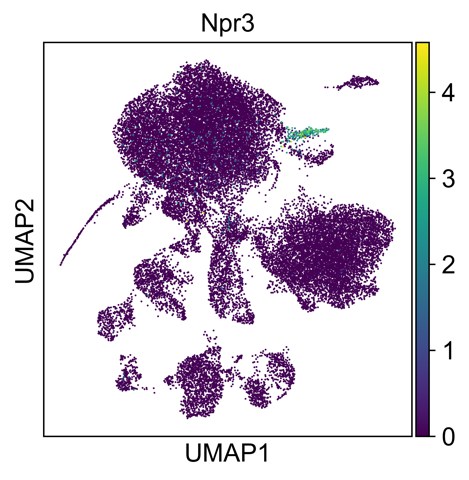


**Endothelial cells**


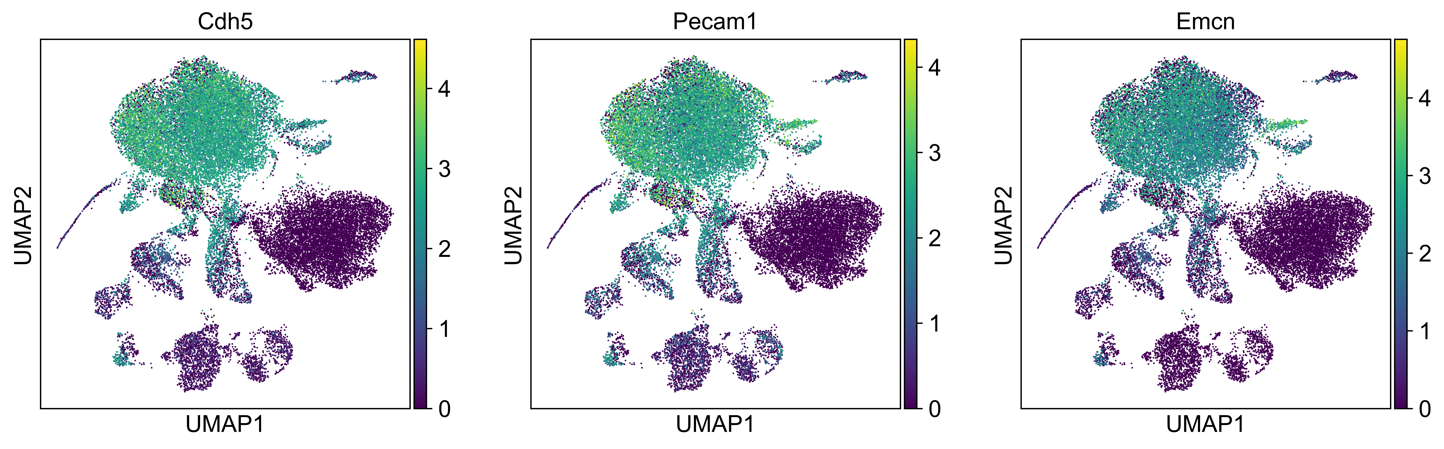


**Fibroblasts**


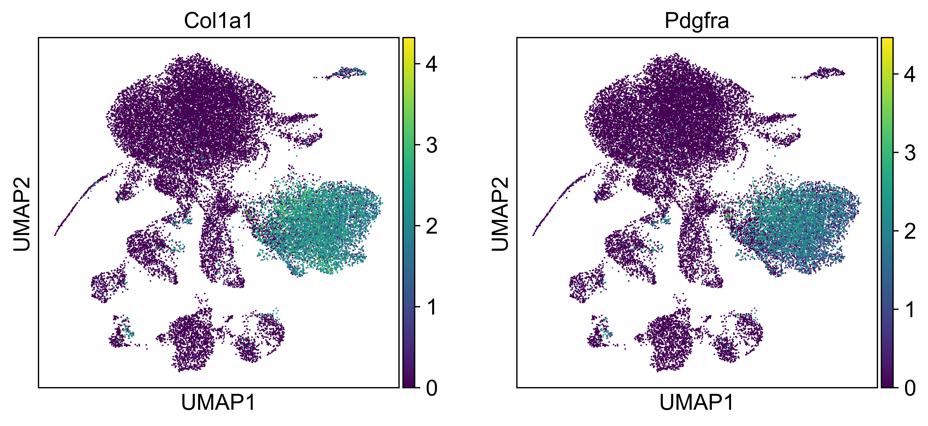


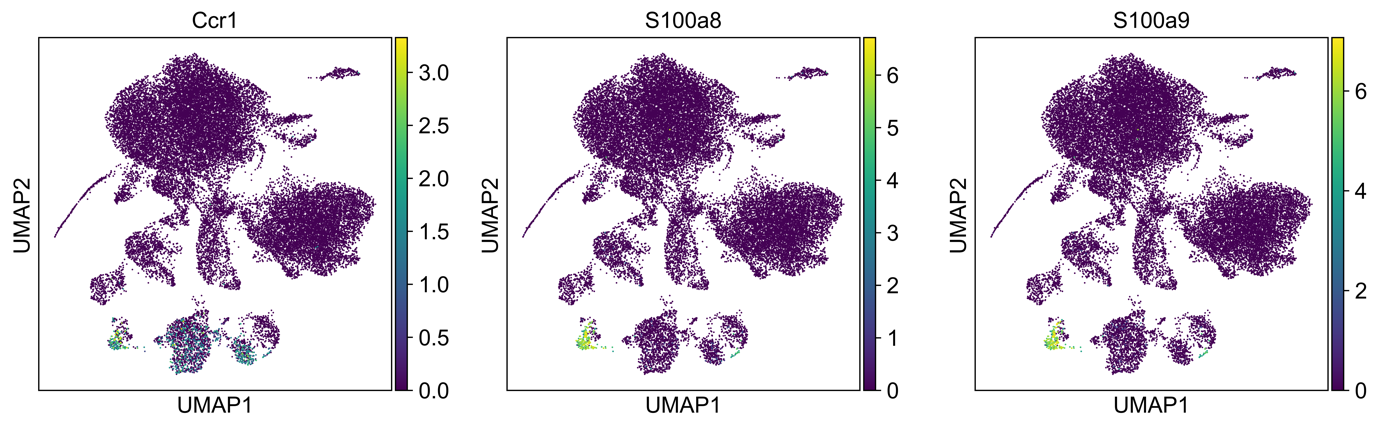


**Granulocytes**

**Macrophages**


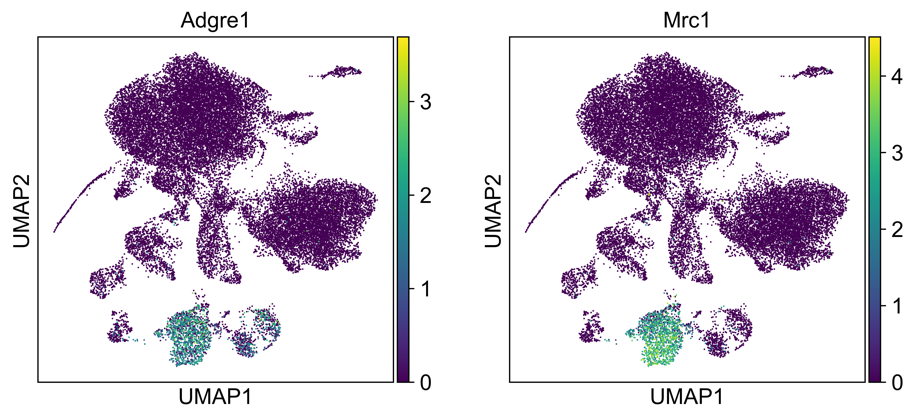


**Monocytes**


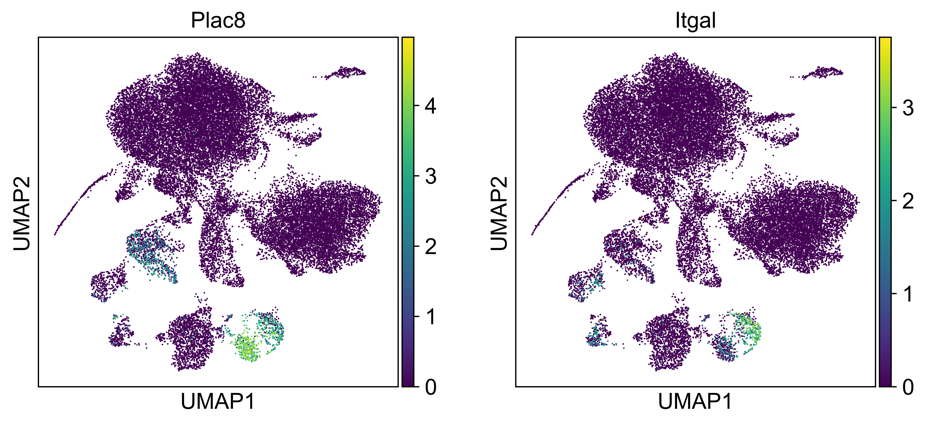


**Pericytes**


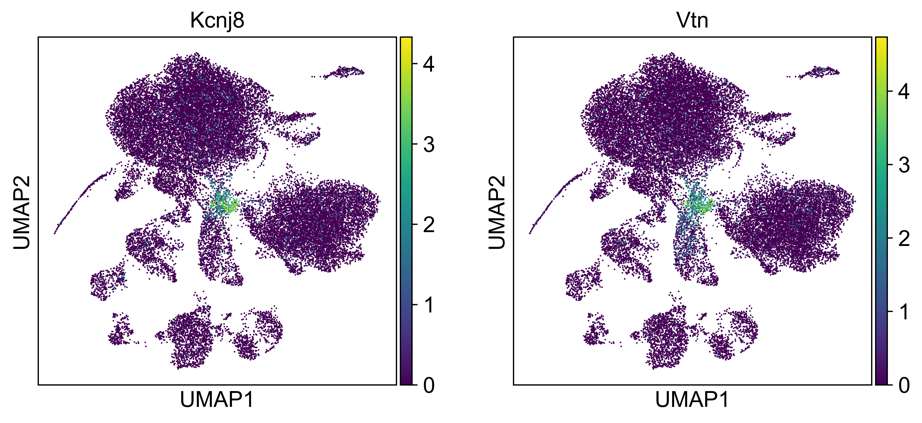


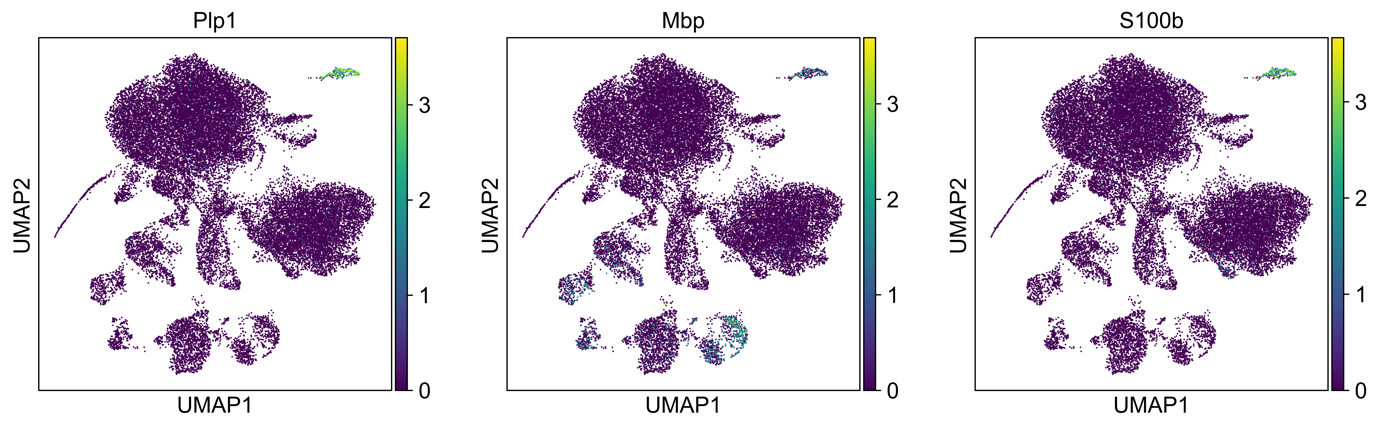


**Schwann cells**

**Smooth muscle cells**


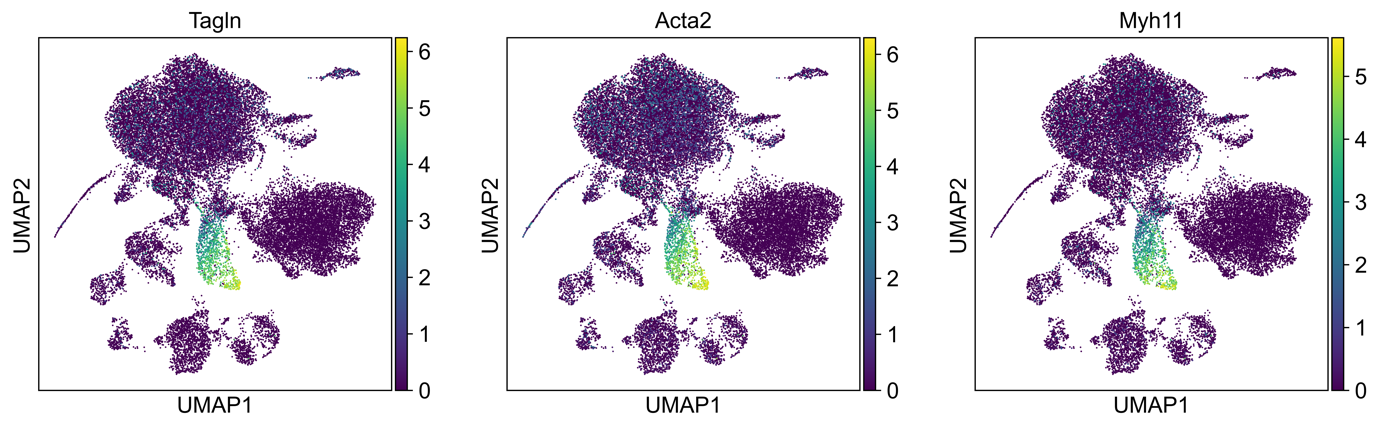


**T cells**


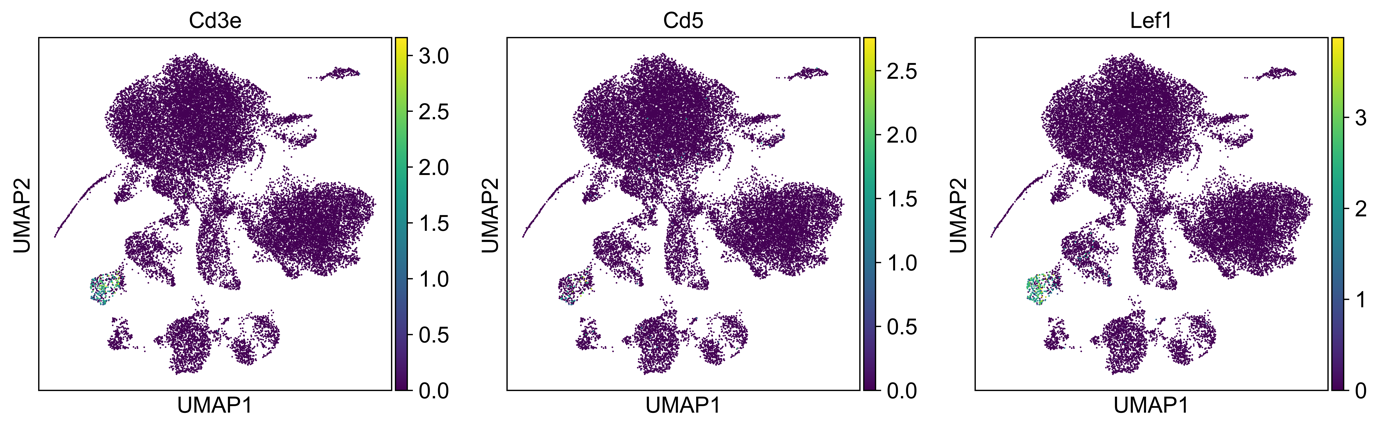


**Figure 2 A priori power analysis for snRNA-seq and proteomics analysis**

**(A)**

snRNA-seq Power Analysis

**
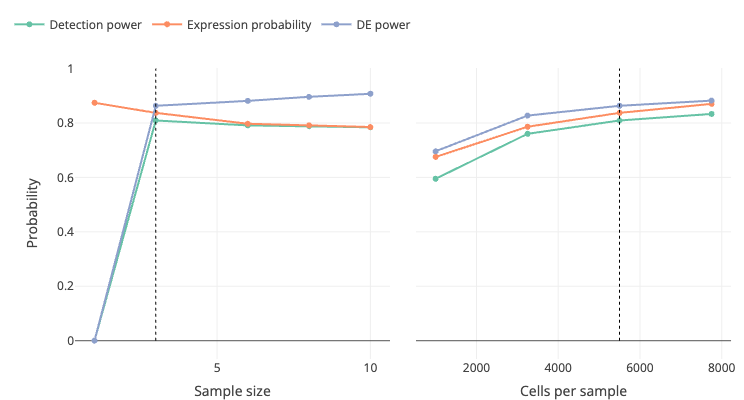
**

**(B)**

**
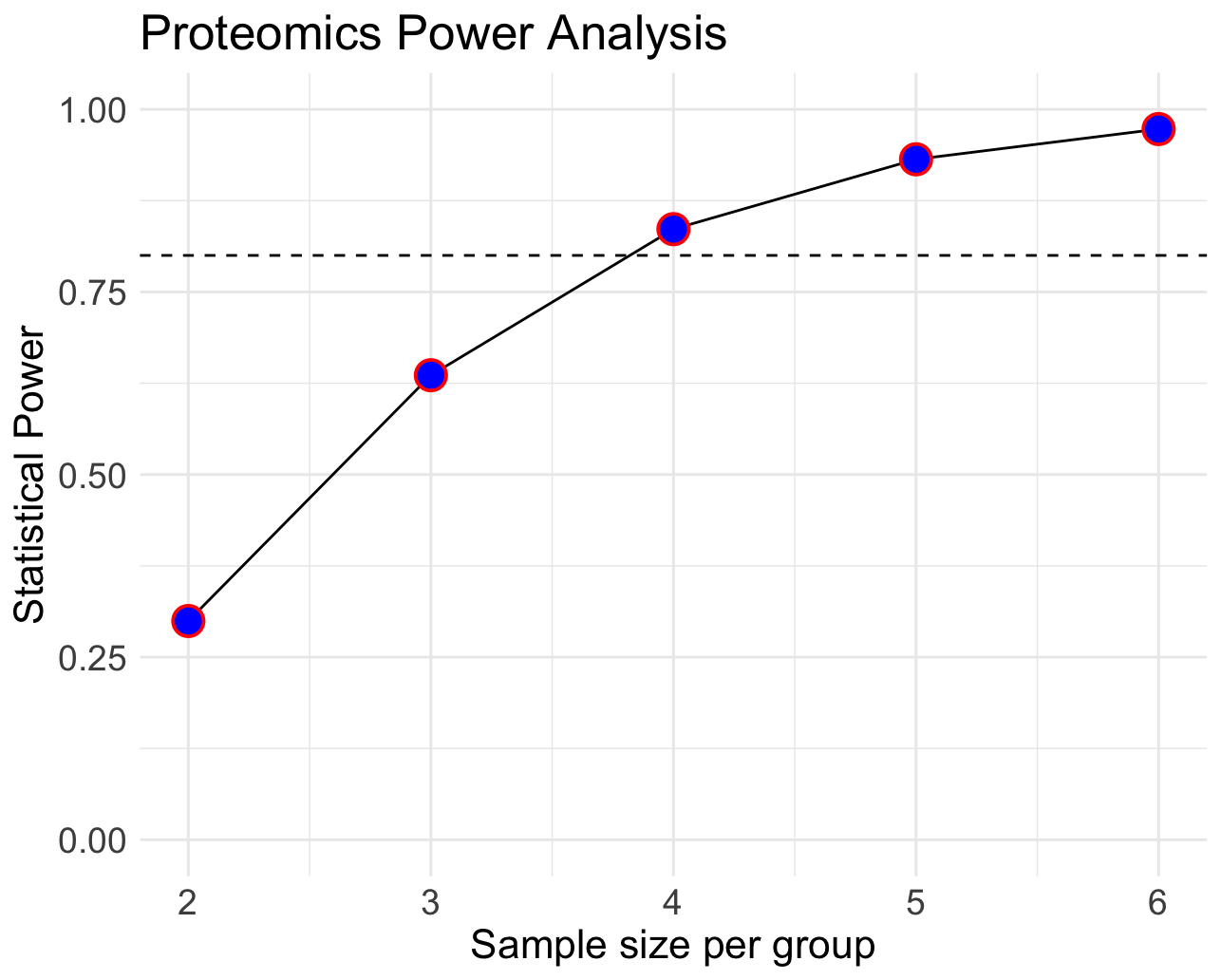
**

**Reference:**

1. McLellan MA, Skelly DA, Dona MSI, Squiers GT, Farrugia GE, Gaynor TL, Cohen CD, Pandey R, Diep H, Vinh A *et al*: **High-Resolution Transcriptomic Profiling of the Heart During Chronic Stress Reveals Cellular Drivers of Cardiac Fibrosis and Hypertrophy**. *Circulation* 2020, **142**(15):1448-1463.

2. Wolock SL, Lopez R, Klein AM: **Scrublet: Computational Identification of Cell Doublets in Single-Cell Transcriptomic Data**. *Cell Syst* 2019, **8**(4):281-291 e289.

3. Yang S, Corbett SE, Koga Y, Wang Z, Johnson WE, Yajima M, Campbell JD: **Decontamination of ambient RNA in single-cell RNA-seq with DecontX**. *Genome Biol* 2020, **21**(1):57.

4. Traag VA, Waltman L, van Eck NJ: **From Louvain to Leiden: guaranteeing well-connected communities**. *Sci Rep* 2019, **9**(1):5233.

5. Dona MS, Hsu I, Rathnayake TS, Farrugia GE, Gaynor TL, Kharbanda M, Skelly DA, Pinto AR: **CLARA: A web portal for interactive exploration of the cardiovascular cellular landscape in health and disease**. *bioRxiv* 2021:2021.2007. 2018.452862.

6. Skelly DA, Squiers GT, McLellan MA, Bolisetty MT, Robson P, Rosenthal NA, Pinto AR: **Single-Cell Transcriptional Profiling Reveals Cellular Diversity and Intercommunication in the Mouse Heart**. *Cell Rep* 2018, **22**(3):600-610.

7. Revelo XS, Parthiban P, Chen C, Barrow F, Fredrickson G, Wang H, Yucel D, Herman A, van Berlo JH: **Cardiac Resident Macrophages Prevent Fibrosis and Stimulate Angiogenesis**. *Circ Res* 2021, **129**(12):1086-1101.

8. Robinson MD, McCarthy DJ, Smyth GK: **edgeR: a Bioconductor package for differential expression analysis of digital gene expression data**. *Bioinformatics* 2010, **26**(1):139-140.

9. Wolf FA, Angerer P, Theis FJ: **SCANPY: large-scale single-cell gene expression data analysis**. *Genome Biol* 2018, **19**(1):15.

10. Phipson B, Sim CB, Porrello ER, Hewitt AW, Powell J, Oshlack A: **propeller: testing for differences in cell type proportions in single cell data**. *Bioinformatics* 2022, **38**(20):4720-4726.

11. Kaspi A, Ziemann M: **mitch: multi-contrast pathway enrichment for multi-omics and single-cell profiling data**. *BMC Genomics* 2020, **21**(1):447.

12. Liberzon A, Birger C, Thorvaldsdottir H, Ghandi M, Mesirov JP, Tamayo P: **The Molecular Signatures Database (MSigDB) hallmark gene set collection**. *Cell Syst* 2015, **1**(6):417-425.

13. Yu F, Teo GC, Kong AT, Frohlich K, Li GX, Demichev V, Nesvizhskii AI: **Analysis of DIA proteomics data using MSFragger-DIA and FragPipe computational platform**. *Nat Commun* 2023, **14**(1):4154.

14. Kong AT, Leprevost FV, Avtonomov DM, Mellacheruvu D, Nesvizhskii AI: **MSFragger: ultrafast and comprehensive peptide identification in mass spectrometry-based proteomics**. *Nat Methods* 2017, **14**(5):513-520.

15. Demichev V, Messner CB, Vernardis SI, Lilley KS, Ralser M: **DIA-NN: neural networks and interference correction enable deep proteome coverage in high throughput**. *Nat Methods* 2020, **17**(1):41-44.

16. Ritchie ME, Phipson B, Wu D, Hu Y, Law CW, Shi W, Smyth GK: **limma powers differential expression analyses for RNA-sequencing and microarray studies**. *Nucleic Acids Res* 2015, **43**(7):e47.
